# Synthesis of Headful Packaging Phages Through Yeast Transformation-Associated Recombination

**DOI:** 10.3390/v17010045

**Published:** 2024-12-31

**Authors:** Cheng Lu, Lan He, Yangyijun Guo, Tingting Wang, Yanrui Ye, Zhanglin Lin

**Affiliations:** 1 School of Biology and Biological Engineering, South China University of Technology, Guangzhou 510006, China; ada1224@foxmail.com (C.L.); helan1994@163.com (L.H.); 201620133950@mail.scut.edu.cn (Y.G.); wangtt@scut.edu.cn (T.W.); 2 School of Biomedicine, Guangdong University of Technology, Guangzhou 510006, China

**Keywords:** headful packaging, synthetic phage, yeast transformation-associated recombination, *Pseudomonas aeruginosa* phage

## Abstract

De novo synthesis of phage genomes enables flexible genome modification and simplification. This study explores the synthetic genome assembly of *Pseudomonas* phage vB_PaeS_SCUT-S4 (S4), a 42,932 bp headful packaging phage, which encapsidates a terminally redundant, double-stranded DNA genome exceeding unit length. We demonstrate that using the yeast TAR approach, the S4 genome can be assembled and rebooted from a unit-length genome plus a minimal 60 bp terminal redundant sequence. Furthermore, we show that S4 can be synthesized from arbitrary starting nucleotides and modified with a red fluorescent protein as a reporter. Additionally, we successfully designed and assembled synthetic S4 phages with reduced genomes, knocking out up to 10 of the 24 hypothetical genes simultaneously, with a combined length of 2883 bp, representing 6.7% of the unit-length genome. This work highlights the potential for engineering simplified, customizable headful packaging phage genomes, providing a foundation for future studies of these phages for potential clinical applications.

## 1. Introduction

Bacteriophages (phages) are viruses that specifically infect bacteria, replicating at the expense of their bacterial hosts [1,2]. In recent years, the use of phages as potential antimicrobial agents has regained substantial attention due to its promise in treating infections caused by antibiotic-resistant bacteria [3,4,5]. Phages possess either double- or single-stranded DNA or RNA genomes. The majority of phages are classified within the order Caudovirales (double-stranded DNA [dsDNA] tailed phages) [6]. Most phages used in phage therapy belong to the families *Myoviridae*, *Siphoviridae*, or *Podoviridae* within this order [7]. dsDNA phages are generally divided into two classes based on how they cleave and package their concatemeric dsDNA [8]: (i) Cleavage at specific sites, such as cos sites (e.g., phage λ) or the 3′ end of a direct terminal repeat (DTR) (e.g., phage T7), which allows them to package unit-length genomes with precise terminal sequences; and (ii) Initiation of cleavage at a specific packaging site (e.g., phage P1, P22) or at random sites (e.g., phage T4), followed by packaging of a terminally redundant, circularly permuted dsDNA that exceeds a unit-length genome until the phage head is full (headful packaging). Since the redundant sequences vary, the genome’s starting and ending positions differ among progeny phages [8,9].

Naturally isolated phages may have inherent limitations, including narrow host ranges, the rapid emergence of phage-resistant bacteria [10,11], and potential risks like the spread of antibiotic resistance [12]. However, these challenges can be addressed through genetic engineering. For example, recombining the genome of the wild-type Enterobacteria phage T3 with plasmid libraries containing mutated versions of the tail fiber gene has enabled the creation of T3 mutants that suppress the emergence of phage-resistant bacteria [13]. On the other hand, the total synthesis of phage genomes allows for the flexible introduction of mutations, deletions, and new genetic traits at virtually any genomic locus, eliminating the need for extensive screening to remove wild-type phages [14,15].

The DNA packaging capacity of dsDNA phage is typically limited, constraining the addition of extra genetic material [16]. However, a significant portion of genes in phage genomes encode hypothetical proteins with unknown functions [17]. Removing these genes creates additional space for genetic engineering [18] and reduces potential risks, making it particularly advantageous for therapeutic applications [19]. Furthermore, these deletions can aid in elucidating the roles and functions of these unknown genes. Therefore, the ability to synthesize complete phage genomes provides an invaluable tool for such efforts.

The synthesis of phage genomes involves two main steps: genome assembly and rebooting [20,21,22,23,24]. During the genome assembly step, smaller DNA fragments are assembled into the full-length phage genome using methods such as Gibson assembly or yeast transformation-associated recombination (TAR) [15,20,21,23]. In the rebooting step, the assembled phage genome is reactivated in a suitable surrogate host cell. *E. coli* or *E. coli* cell-free systems have been used for phages infecting Gram-negative bacteria, and L-form *Listeria* or NEST transformation for phages infecting Gram-positive bacteria [20,21,23,24,25].

Gibson assembly enables the in vitro formation of circularized synthetic DNA that mimics the circular genome replication intermediates of phages, making it broadly applicable for the synthesis of various phage types such as cos, DTR, and headful packaging phages [20,22,26,27]. However, the efficiency of Gibson assembly decreases as the number and size of DNA fragments increase [28,29]. On the other hand, the yeast TAR approach leverages the efficient recombination machinery of *Saccharomyces cerevisiae* to reliably assemble entire phage genomes into a yeast artificial chromosome (YAC) vector from multiple DNA fragments [21]. To date, several dsDNA DTR phages have been successfully synthesized using yeast TAR [19,21,30]. However, progress with headful packaging phages has been limited, likely at least in part due to the imprecise terminal redundant sequences in their genomes [8,9,24], which complicates the assembly of an exact sequence into the YAC vector.

*Pseudomonas* phages are a promising option for the future treatment of infections caused by multidrug-resistant *Pseudomonas aeruginosa* (*P. aeruginosa*) [3,4,14,15]. We have focused on headful packaging *Pseudomonas* phages, which account for approximately one-third of the sequenced *Pseudomonas* phages in the GenBank database (Table S1). In this study, we isolated the headful packaging phage vB_PaeS_SCUT-S4 (S4) from *P. aeruginosa* and explored the synthesis of its genome using the yeast TAR method, as consistent synthesis could not be achieved using Gibson assembly. We successfully synthesized phage S4 by assembling a unit-length genome with a terminal redundant sequence as short as 60 bp. Additionally, we demonstrated that headful packaging phages can be synthesized with arbitrarily designated starting nucleotides. These allowed us to construct four synthetic phages with reduced genomes (S4-Δgp39–43, S4-Δgp44–48, S4-Δgp50–55, and S4-Δgp39–48), simultaneously knocking out up to 10 of the 24 hypothetical genes. These findings open new possibilities for developing customized phage-based therapeutics through engineering headful packaging phages [31].

## 2. Materials and Methods

### 2.1. Strains, Culture Conditions and Plasmids

*P. aeruginosa* ATCC 9027 and *S. cerevisiae* ATCC MYA3666 were purchased from the American Type Culture Collection (Manassas, VA, USA). *E. coli* NEB 10-beta was purchased from New England Biolabs (Beijing) Ltd. (Beijing, China). Phage vB_PaeS_SCUT-S4 (GenBank accession number: MK165658.1) was isolated during this study. Bacterial strains and phages were routinely cultured at 37 °C in LB broth. *S. cerevisiae* ATCC MYA3666 was cultured in a YPD medium, while yeast transformants were grown in a synthetic dropout medium lacking histidine (SD-His) (FunGenome Technology, Beijing, China) at 30 °C. The YAC vectors pRSII313 and pYEP-II (GenBank accession number: PQ666752) were utilized for the TAR assembly of the phage genomes. The vector pRSII313, which contains a yeast CEN/ARS element and a HIS3 marker (CEN6/ARS4-HIS3), was purchased from Addgene (Watertown, MA, USA) (#35449). The vector pYEP-II, which incorporates the CEN6/ARS4-HIS3 element from pRSII313 into the bacterial artificial chromosome (BAC) vector pBeloBAC11, was previously constructed in our laboratory.

### 2.2. Phage Isolation and Morphological Characterization

Phage S4 was isolated from a small pond in Guangzhou, China, using *P. aeruginosa* ATCC 9027 as the host, following the enrichment method as previously described [32]. Briefly, 500 mL of aqueous samples were collected and centrifuged, and the concentrated supernatant was filtered through a 0.45 μm filter before being incubated with *P. aeruginosa* ATCC 9027 for 24 h at 37 °C. After incubation, the cell debris from *P. aeruginosa* was removed through centrifugation and filtration. The resulting supernatant was then plated on double-layer agar plates to detect phage plaques, and candidate plaques were purified through three rounds of plating.

### 2.3. Transmission Electron Microscopy

Phage S4 was morphologically characterized using transmission electron microscopy. In brief, purified phage S4 was spotted on a 400-mesh carbon-coated grid and negatively stained with 2% phosphotungstic acid (pH 6.5) [33]. Observations were conducted using a Hitachi transmission electron microscope operating at 80 kV.

### 2.4. Isolation of the Phage S4 Genomic DNA

The genomic DNA of phage S4 was isolated using a previously described method [32] with minor modifications. Specifically, a 100 mL culture of exponentially growing *P. aeruginosa* ATCC 9027 cells were infected with phage S4 at a multiplicity of infection (MOI) of approximately 0.1 and incubated until lysis occurred. The lysate was extracted with chloroform, and the supernatant was treated with DNase I and RNase A, each at a final concentration of 10 μg/mL, to eliminate host nucleic acids. Phage particles were precipitated using PEG 8000 and subsequently centrifuged. The phage pellet was then resuspended in SM buffer (100 mM NaCl, 8 mM MgSO_4_, and 50 mM Tris-HCl, pH 7.5) and treated with chloroform. The supernatant was incubated with DNase I and RNase A, each at a final concentration of 10 μg/mL, followed by treatment with SDS and proteinase K. After incubation in a cetyltrimethylammonium bromide (CTAB)/NaCl solution (16.7% *w*/*v* CTAB, 1.1 M NaCl), the aqueous phase was extracted through a series of treatments with chloroform, phenol/chloroform/isoamyl alcohol (25:24:1), and chloroform, ensuring gentle mixing to minimize DNA shearing. Finally, the phage DNA in the aqueous phase was precipitated with isopropanol, washed with 70% (*v*/*v*) ethanol, and dissolved in ddH_2_O.

### 2.5. Genome Sequencing, Assembly and Annotation

The genomic DNA was sequenced by Personal Biotechnology Co., Ltd. (Shanghai, China) on the Illumina MiSeq PE250 sequencing platform. Library preparation was performed with the KAPA Hyper Prep Kit (KAPA Biosystems, Wilmington, MA, USA), and sequencing utilized the MiSeq Reagent Kit v2 (Illumina, San Diego, CA, USA). Genome assembly and annotation were carried out as previously described [32]. Concisely, sequencing reads were filtered and assembled using ABYSS 2.0.2 [34], and the contigs were manually assembled based on overlaps of more than 40 bp to obtain the final scaffold. The sequence coverage was visualized using Geneious 10.2.3. The packaging mechanisms and the *pac* site were predicted using PhageTerm, which is available on the public Galaxy server (http://galaxy.pasteur.fr/, accessed on 23 December 2024) [9]. Genome annotation was performed with RAST (http://rast.nmpdr.org/, accessed on 23 December 2024) [35], Glimmer (http://ccb.jhu.edu/software/glimmer/index.shtml, accessed on 23 December 2024) [36], and GeneMarkS (http://opal.biology.gatech.edu/GeneMark/genemarks.cgi, accessed on 23 December 2024) [37]. tRNA genes were predicted using tRNA-Scan (http://lowelab.ucsc.edu/tRNAscan-SE/, accessed on 23 December 2024) [38] and ARAGORN (http://mbio-serv2.mbioekol.lu.se/ARAGORN/, accessed on 23 December 2024) [39].

### 2.6. Preparation of PCR Products for Yeast TAR Assembly of Phage Genomes

Overlapping DNA fragments with 60 bp homologies were utilized for TAR cloning. The vector pYEP-II was linearized by digestion with SphI and HindIII, or alternatively, by PCR using primers that included 30 bp overlaps with the phage genome fragments. The vector pRSII313 was also linearized via PCR with primers containing 30 bp overlaps with the phage genome fragments. Phage genome fragments were amplified from the purified genomic DNA of phage S4. The red fluorescent protein (RFP) expression cassette (GenBank accession number: PQ677678), consisting of a monomeric RFP mCherry gene regulated by a strong P*tac* promoter, was amplified from pT008 (an unpublished RFP-expressing plasmid from our laboratory). All DNA fragments were PCR-amplified using KOD FX DNA Polymerase (TOYOBO, Shanghai, China). Details regarding the primers used for fragment amplification and the PCR products are listed in Table S2. PCR products were verified by 1% agarose gel electrophoresis, with fragment sizes compared against a 1 kb extend DNA ladder (New England Biolabs, Beijing, China). Target DNA bands were excised, and DNA was purified using a HiPure Gel Pure DNA Micro kit (Magen, Guangzhou, China).

### 2.7. Yeast Transformation

Yeast-competent cells were prepared using the EZ Yeast Transformation II Kit (Zymo Research Corp., Irvine, CA, USA) according to the manufacturer’s instructions. For transformation, 200 ng of each DNA fragment and 100 ng of linearized vector were mixed with 100 μL of yeast-competent cells and 500 μL of EZ3 solution. To minimize DNA shearing, reagents were added slowly and mixed gently. The mixture was incubated at 30 °C for 45 min and then concentrated to 200 μL by centrifugation at 500× *g* for 5 min. The resulting pellet was resuspended, plated onto SD-His plates, and incubated at 30 °C for 2–4 days. Positive yeast colonies were screened by colony PCR, as previously described [40]. In short, yeast transformants were patched on SD-His plates and transferred into 20 µL of 25 mM NaOH, then incubated at 95 °C for 30 min to lyse the yeast cells. The yeast lysates were subjected to PCR to verify the correct junctions between adjacent fragments. The primers used for junction verification are listed in Table S3.

### 2.8. Extraction of Yeast-Cloned Synthetic Phage Genomes

The extraction of yeast-cloned synthetic phage genomes was performed according to a previously described protocol [40] with minor modifications. Verified yeast transformants were cultured in 10 mL SD-His liquid medium for 36–48 h at 30 °C with shaking at 220 rpm and then collected by centrifugation at 500× *g* for 10 min. The cells were resuspended in 500 µL of P1 buffer (Tiangen, Beijing, China), supplemented with 20 µL of zymolyase-20T (MP Bio, Eschwege, Germany; 10 mg/mL) and 2 µL of β-mercaptoethanol (Sigma Aldrich, St. Louis, MO, USA). The mixture was incubated at 37 °C for 2–3 h. 60 µL of 2% (*w*/*v*) SDS was added, and the solution was incubated for an additional 15 min at 70 °C. Subsequently, 60 µL of 5 M potassium acetate was added, and the solution was incubated on ice for 5 min. The DNA in the supernatant was precipitated with isopropanol, washed with 70% (*v*/*v*) ethanol, and finally dissolved in 40 µL of ddH_2_O.

### 2.9. Rebooting of Phages

To reboot phage S4 or the synthetic phages, 10 μL of extracted DNA was electroporated into 100 µL of *E. coli* NEB 10-beta in 2-mm gap electroporation cuvettes (Molecular Bio Products, San Diego, CA, USA) and a Gene Pulser Xcell electroporation system (BioRad, Shanghai, China). The electroporation conditions were set to 2500 V, 25 µF, and 200 Ω for *E. coli* cells. After electroporation, the cells were incubated at 37 °C for 2 h in 1 mL SOC medium. Rebooted phages were released by adding 10 µL of chloroform to the cells, followed by centrifugation at 12,000× *g* for 1 min. To test the lytic activity of the phages, approximately 1.1 mL of harvested supernatant from chloroform-treated cells were mixed with 300 µL of overnight cultures of *P. aeruginosa* ATCC 9027 and 5 mL of molten LB soft agar (LB + 0.6% agar) tempered to 50 °C. This mixture was then poured onto LB plates and incubated overnight at 37 °C. The all-*E. coli* TXTL system (Arbor Biosciences, Ann Arbor, MI, USA) was utilized as the cell-free system for rebooting phage S4. *E. coli* NEB 10-beta served as the one-time propagation host of the phages.

### 2.10. Determination of Phage Titer

Phages were serially diluted in SM buffer and mixed with 300 µL of overnight cultures of *P. aeruginosa* ATCC 9027 and 5 mL of molten LB soft agar tempered to 50 °C. The mixture was poured onto LB plates and incubated at 37 °C for 12–24 h. After incubation, phage plaques were counted, and the plaque-forming units (PFU) per mL were calculated.

### 2.11. Verification of Packaged Synthetic Phage Genomes

The RFP cassette and the junctions between the first and the last DNA fragments were PCR-verified using the extracted genomic DNA from the synthetic phages and appropriate primers (Table S3). The PCR products were confirmed by Sanger sequencing. Additionally, the genomic DNA of the synthetic phages was analyzed by digestion with restriction endonucleases SnaBI or EcoRI as specified in the manufacturer’s instructions. The genomic DNA of the synthetic phages S4-a1, S4-b1, S4-a1-RFP, and S4-b1-RFP was digested with SnaBI, while the genomic DNA of the other synthetic phages was digested with EcoRI. The genomic DNA of the wild-type phage S4 served as a control and was digested with both SnaBI and EcoRI.

### 2.12. Measurement of Host Killing Curves and RFP Fluorescence in P. aeruginosa Cells

To monitor host killing caused by infection with wild-type and synthetic S4 phages, as well as RFP expression in phage-infected *P. aeruginosa* cells, overnight cultures of *P. aeruginosa* ATCC 9027 cells were diluted 1:100 into LB medium and grown for 2 h at 37 °C until they reached log phase (OD_600_ 0.5, ~3 × 10^8^ cells/mL). Approximately 3 × 10^7^ *P. aeruginosa* cells were then inoculated with phage (3 × 10^7^ PFU) at MOI of ~1 in 96-well microtiter plates. The optical density at 600 nm (OD_600_) and the relative fluorescent unit (RFU) were measured every 10 min for 24 h at 37 °C, 240 rpm using a Tecan infinite M200 PRO microplate reader (Zürich, Switzerland).

An equal volume of LB medium was used as the blank control, and OD_600_ and RFU values obtained with the blank were subtracted from the experimental values. All experiments were performed in triplicate.

## 3. Results

### 3.1. Isolation and Characterization of the P. aeruginosa Phage vB_PaeS_SCUT-S4

**Figure 1:**
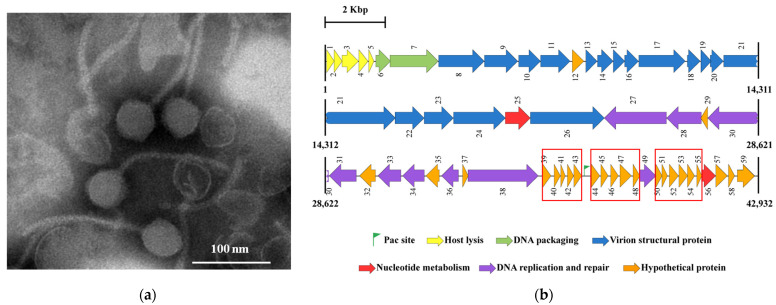
Characterization of the *P. aeruginosa* phage S4. (**a**) Transmission electron microscopy image of phage S4. The scale bar represents 100 nm. (**b**) Genetic map of the phage S4 genome. Predicted ORFs are indicated by arrows and color-coded according to their predicted functions. The predicted *pac* site is marked with a flag. The regions of the three clusters of genes encoding hypothetical proteins are marked with red square frames.

The *P. aeruginosa* phage vB_PaeS_SCUT-S4 (S4) was isolated from aquatic environmental samples using *P. aeruginosa* ATCC 9027 as the host strain. Phage S4 formed clear plaques on double-layer agar plates inoculated with *P. aeruginosa* ATCC 9027, indicating its lytic nature. Transmission electron microscopy (TEM) revealed that phage S4 has an icosahedral head measuring 59.6 ± 2.1 nm in diameter and a long, non-contractile tail measuring 196.0 ± 4.1 nm (Figure 1a).

Phage S4 was further characterized through whole genome sequencing. The sequencing data revealed that the phage S4 genome (GenBank accession number: MK165658.1) consists of a 42,932 bp linear double-stranded DNA with a GC content of 53.8%. It encodes 59 predicted open reading frames (ORFs), covering approximately 95% of the genome (Figure 1b). Of these, 35 ORFs have assigned functions, while the remaining 24 encode hypothetical proteins (Table S4). Genome sequencing, combined with TEM analysis, indicates that phage S4 belongs to the *Siphoviridae* family within the *Caudovirales* order [41]. PhageTerm [9] analysis suggested that the S4 genome has circularly permuted termini and a predicted *pac* site at nucleotide position 37,176 (Figure 1b), indicating that it utilizes a P1-type headful packaging mechanism [9]. No lysogeny-associated genes (e.g., those encoding integrase, excisionase, or repressor) were detected, confirming that phage S4 is a lytic phage.

### 3.2. Synthesis of P. aeruginosa Phage S4 Through Yeast TAR Assembly

We first evaluated the rebooting of purified genomic DNA of native phage S4 in *E. coli* NEB 10-beta and a cell-free system (all-*E. coli* TXTL system). Plaque assays showed that the phage S4 genomic DNA successfully rebooted into functional phages in *E. coli* NEB 10-beta cells (Figure S1a), even though phage S4 cannot naturally infect this *E. coli* strain (Figure S1b). This indicates that *E. coli* was successfully used as a surrogate host for phage S4 to replicate and package its genome, producing intact phage S4 particles. However, the rebooting attempt in the *E. coli* TXTL cell-free system was unsuccessful.

**Figure 2:**
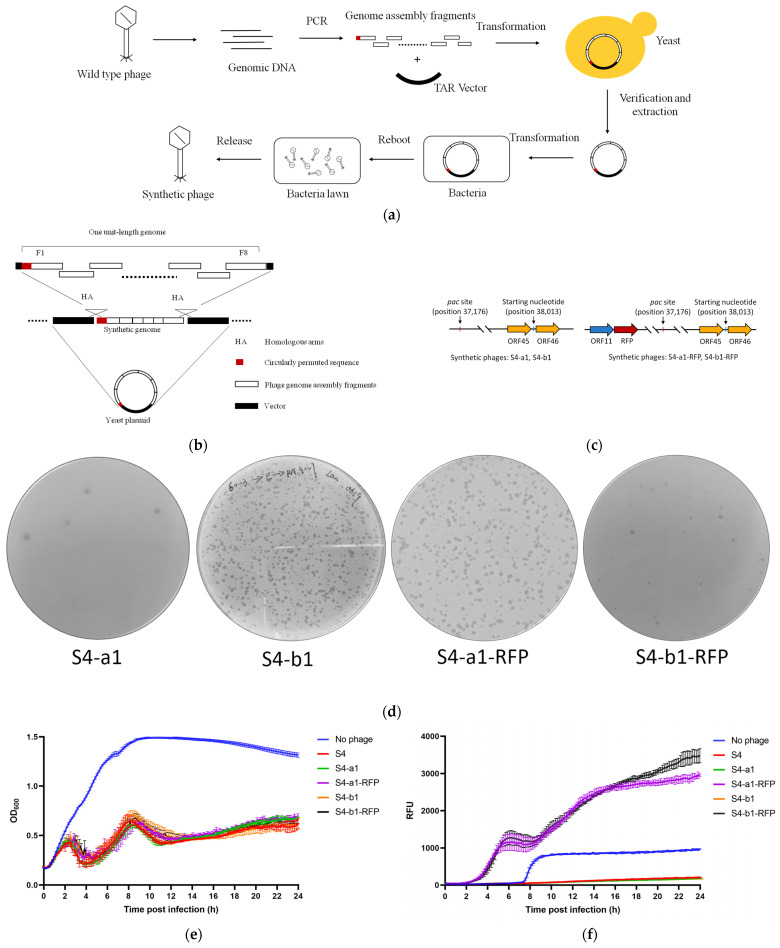
Synthesis and functional characterization of the headful packaging phage S4 through yeast TAR method. (**a**) Workflow for headful packaging phage assembling and rebooting through the yeast TAR approach. Phage genome fragments are amplified by PCR and assembled into the yeast TAR vector within yeast cells. Correctly assembled phage genomes are extracted from yeast clones and transformed into bacteria. Rebooted phages are verified by plaque formation on a bacterial lawn. (**b**) Detailed strategy for phage genome assembly. The fragments and a 60 bp terminal redundant sequence of the headful packaging phage are assembled into the linearized yeast TAR vector, generating a yeast plasmid that carries the synthetic phage genome. (**c**) Schematic of genome initiation point for synthetic phages S4-a1, S4-b1, S4-a1-RFP, and S4-b1-RFP, highlighting the RFP cassette incorporated in S4-a1-RFP and S4-b1-RFP. The predicted *pac* site and the designated starting nucleotide are marked based on the S4 genome sequence (not to scale). ORF sizes and distances are also not to scale. (**d**) Plaque morphology observed for synthetic phages S4-a1, S4-b1, S4-a1-RFP, and S4-b1-RFP on *P. aeruginosa* ATCC 9027. (**e**) Host killing curves of *P. aeruginosa* ATCC 9027 upon infection with wild-type and synthetic S4 phages. (**f**) RFP expression in *P. aeruginosa* ATCC 9027 cells infected by phages. Data represent means and standard deviations from triplicate experiments, each performed in duplicate.

We then attempted to synthesize the unit-length genome (42,932 bp) of phage S4 using the Gibson assembly method [20] but were unable to consistently recover intact phages. Consequently, we switched to the yeast TAR method. The S4 genome was divided into eight fragments (F1 through F8), ranging from 4.8 to 6.7 kb in length. These fragments were amplified by PCR from purified phage S4 genomic DNA, with adjacent fragments containing 60 bp homology arms (Figure 2a,b). The first and last fragments (F1 and F8) were amplified with added homology arms to facilitate vector integration (Table S2). Since the physical ends of the phage S4 genome are not fixed, we arbitrarily designated nucleotide position 38,013—located between two unknown ORFs (ORF45 and ORF46) and downstream of the predicted *pac* site—as the starting nucleotide of the unit-length genome (Figure 2c). The unit-length genome of phage S4 was successfully assembled into both the commercial *E. coli*-yeast shuttle YAC vector pRSII313 [42] and a custom vector, pYEP-II, using yeast TAR cloning (Figure 2a,b). pYEP-II was constructed by inserting the CEN6-ARS4-HIS3 sequence from pRSII313 into the bacterial BAC vector pBeloBAC11 (see Methods and Figure S2), providing it with the capacity to clone the S4 genome in *E. coli*. Colony PCR showed that 87.5% (14/16) of yeast clones contained correctly assembled pRSII313-S4-a0 plasmids and 37.5% (6/16) contained correctly assembled pYEP-II-S4-b0 plasmids (Figures S3 and S4). To reboot the synthesized phage genome, we extracted the plasmids from yeast cells and transformed them into *E. coli* NEB 10-beta. The bacterial cells were treated with chloroform, and the supernatants were subjected to plaque assay. However, no plaques were observed on double-layer agar plates in two independent replicates.

Since headful packaging phages contain terminally redundant double-stranded DNA longer than the unit-length genome, we hypothesized that this redundant sequence is essential for successful synthesis. Although the exact length and composition of the terminally redundant sequence in the S4 genome are unknown, we added a 60 bp terminal redundant sequence to the 5′ end of fragment F1, overlapping with the last 60 bp of fragment F8 (Figure 2a–c and Table S2). The DNA fragments were assembled into the yeast TAR vectors pRSII313 and pYEP-II, generating plasmids pRSII313-S4-a1 and pYEP-II-S4-b1 with assembly success rates of 50% (4/8) and 71.4% (5/7), respectively (Figure S5 and Table 1). The plasmids were then extracted from yeast and transformed into *E. coli* NEB 10-beta for rebooting. Plaque assays confirmed that the synthetic S4 genomes produced functional phages (referred to as S4-a1 and S4-b1; see Table 1 and Figure 2d). These results demonstrate that functional headful packaging phage S4 can be synthesized with a one-unit genome plus a 60 bp terminally redundant sequence despite the natural length of the terminal redundancy being unknown.

Next, we used RFP as a reporter to engineer custom headful packaging phages. The RFP cassette (RFP gene under the control of a strong P*tac* promoter) was inserted downstream of ORF11, which encodes the major capsid protein in the S4 genome (Figure 2c and Table 1). To achieve this, F3 was further divided into two overlapping fragments (Table S2) and assembled with the RFP cassette and the remaining fragments in yeast, resulting in the plasmids pRSII313-S4-a1-RFP and pYEP-II-S4-b1-RFP, with assembly success rates exceeding 60% (Figures S6 and S7, and Table 1). Plaque assays confirmed that all RFP-incorporated phages were successfully rebooted (designated as S4-a1-RFP and S4-b1-RFP, respectively; Figure 2d). Additionally, we observed that more than 30% of the extracted synthetic phage genomes could be rebooted into functional phages, consistent with a previous study on DTR phage synthesis [21].

The genomic DNA of the synthetic phages, both with and without the RFP insertion, was isolated for verification. The incorporation of the RFP cassette was first confirmed by PCR, with the PCR products from synthetic phages with RFP showing a size approximately 800 bp larger than that of the wild-type fragment (Figure S8a), consistent with the size of the RFP cassette (783 bp). These results were further validated by Sanger sequencing. To ensure that the synthetic phage genomes did not contain vector sequences, we amplified the junctions between the first and last DNA fragments (spanning the intergenic region between ORF45/46, see Figure 2c) from the genomic DNA of the synthetic phages and subjected them to Sanger sequencing. The junction sequences of all synthetic phages matched those of the wild-type phage S4 (Figure S9), confirming that the successful rebooting of the synthetic phage genomes occurred independently of vector sequences.

Restriction fragment length polymorphism (RFLP) analysis was performed to further verify the incorporation of the RFP cassette and to determine whether any other regions of the genome were altered. The genomic DNA of the synthetic phages S4-a1 and S4-b1 exhibited identical restriction enzyme digestion patterns to that of the wild-type phage S4. In contrast, the synthetic phages S4-a1-RFP and S4-b1-RFP displayed a larger DNA band compared to the wild-type, further confirming the successful incorporation of the RFP cassette (Figure S8b).

The effect of the synthetic phages on *P. aeruginosa* ATCC 9027 planktonic cultures, as well as the RFP expression in phage-infected host bacteria, was then assessed. The growth curves of *P. aeruginosa* ATCC 9027 treated with the synthetic phages were comparable to those treated with the wild-type phage S4 (Figure 2e). Additionally, cultures exposed to the synthetic phages containing the RFP cassette exhibited significant fluorescence after 3 h of co-cultivation with *P. aeruginosa* ATCC 9027 (Figure 2f). The increase in fluorescence observed at 8 h may be attributed to fluorescent metabolites, such as pyoverdine, whose emission spectrum partially overlaps with RFP [43].

### 3.3. Synthesis of P. aeruginosa Phage S4 with Arbitrarily Designated Starting Nucleotides

**Figure 3:**
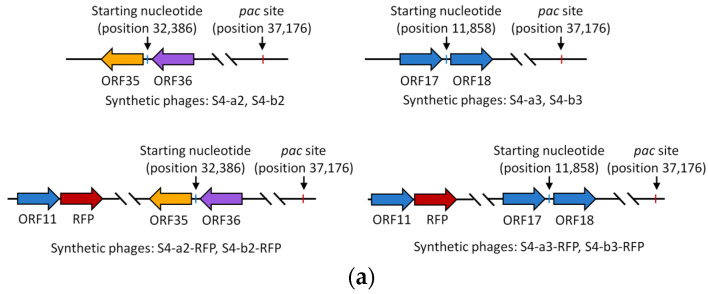

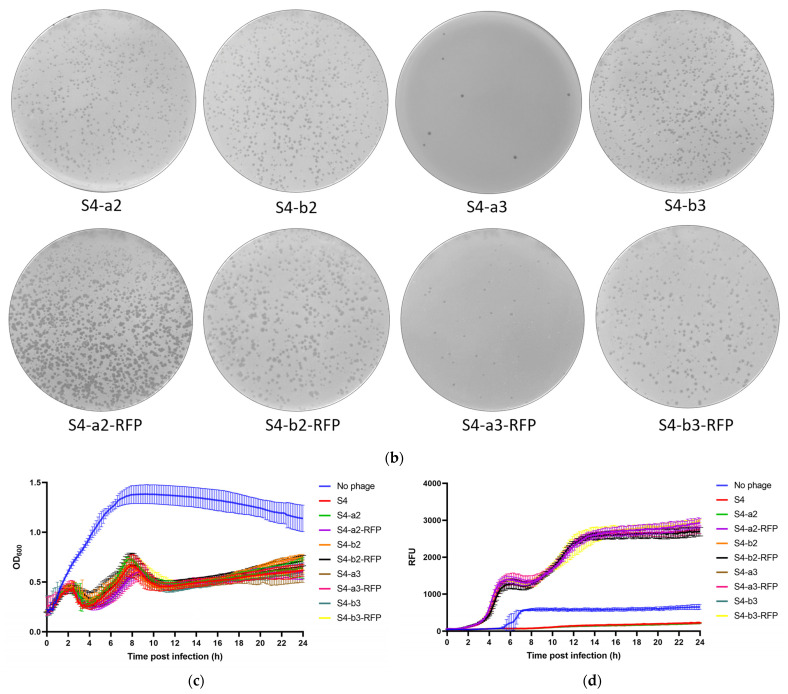
Synthesis of the headful packaging phage S4 with various arbitrarily designated starting nucleotides. (**a**) Schematic of genome initiation point for synthetic phages with arbitrarily designated starting nucleotide. (**b**) Plaque morphology observed for synthetic phages S4-a2, S4-b2, S4-a3, S4-b3, S4-a2-RFP, S4-b2-RFP, S4-a3-RFP and S4-b3-RFP on *P. aeruginosa* ATCC 9027. (**c**) Host killing curves of *P. aeruginosa* ATCC 9027 upon infection with wild-type and synthetic S4 phages. (**d**) RFP expression in *P. aeruginosa* ATCC 9027 cells infected by phages. Data represent means and standard deviations from triplicate experiments, each performed in duplicate.

Given that the redundant sequences in headful packaging phage genomes are not fixed [8,9], we further hypothesized that these phages could be synthesized from arbitrarily designated starting nucleotides. To test this hypothesis, we assembled two additional versions of the phage S4 genome, each with different starting positions: one starting at position 32,386, located just upstream of the *pac* site (between ORF35 and ORF36), and the other starts at position 11,858, further from the *pac* site (between ORF17 and ORF18) (Figure 3a and Table 1). Furthermore, we introduced an RFP cassette downstream of ORF11 in the synthetic genomes (Table 1). Each genome was divided into eight or ten fragments (for RFP incorporation), with the 60 bp terminally redundant sequence introduced at the 3′ end of the last fragment (F8). These fragments were then assembled into pRSII313 and pYEP-II vectors in yeast, yielding assembly success rates ranging from 25% to 87.5% (Figures S10–S15 and Table 1). The synthetic phage genomes were subsequently isolated from yeast and rebooted in *E. coli* NEB 10-beta. Plaque assay confirmed that all synthetic S4 genomes produced functional phages (Figure 3b and Table 1). PCR analysis verified the incorporation of the RFP cassette (Figure S16a), and sequencing confirmed that the junctions between the first and last fragments matched the wild-type phage S4 (Figure S16b). The synthetic phages displayed expected restriction enzyme digestion patterns (Figure S17) and exhibited similar host-killing dynamics as the wild-type S4 phage (Figure 3c). Additionally, the RFP-incorporated phages demonstrated strong fluorescence (Figure 3d). These results confirm that headful packaging phages can be synthesized using the yeast TAR approach, with genomes assembled at arbitrarily designated starting nucleotides.

### 3.4. Synthesis of P. aeruginosa Phage S4 with Reduced Genome

**Figure 4:**
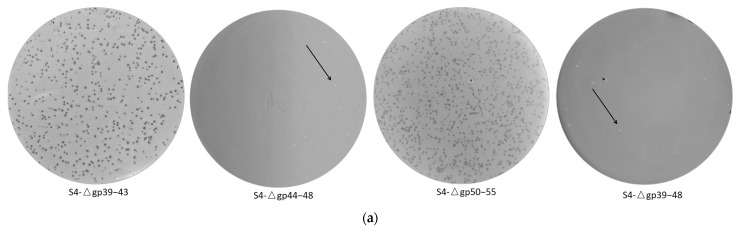

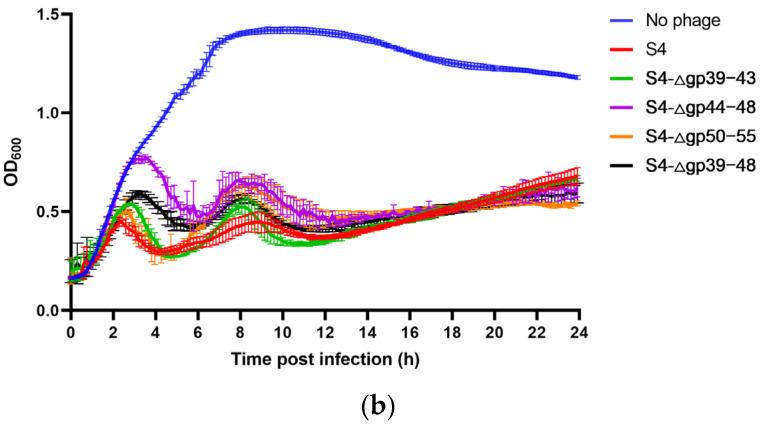
Synthesis of *P. aeruginosa* phage S4 with a reduced genome. (**a**) Plaque morphology observed for synthetic phages S4-Δgp39–43, S4-Δgp44–48, S4-Δgp50–55, and S4-Δgp39–48 on *P. aeruginosa* ATCC 9027. (**b**) Host killing curves of *P. aeruginosa* ATCC 9027 upon infection with wild-type and synthetic S4 phages. Data represent means and standard deviations from triplicate experiments, each performed in duplicate.

Then, we evaluated the feasibility of constructing synthetic phage S4 with reduced genomes by removing genes encoding hypothetical proteins. In the annotated S4 genome, three clusters of genes encoding hypothetical proteins are separated by the *pac* site and the gene encoding Vsr endonuclease (ORF49): the first cluster is located between ORF39 and ORF43, the second between ORF44 and ORF48, and the third between ORF50 and ORF55, as highlighted by the red square frames in Figure 1b. Based on this, we constructed three synthetic phages with reduced genomes using the yeast TAR approach: S4-Δgp39–43, S4-Δgp44–48, and S4-Δgp50–55. To streamline the synthesis, we exclusively used pRSII313, as it yielded results similar to those from pYEP-II in previous experiments in this study. Each genome was divided into eight or nine fragments, with a 60 bp terminally redundant sequence added at the 3′ end of the last fragment (F8). The assembly success rates were 2/16, 1/16, and 2/16, respectively (Figures S18–S20 and Table 1). After isolating the synthetic phage genomes from yeast, we rebooted them in *E. coli* NEB 10-beta. Plaque assay indicated that all synthetic S4 genomes produced functional phages (Figure 4a and Table 1). PCR verification of the resulting plaques confirmed successful knockouts in the phage genome (Figure S21a), and the synthetic phages exhibited the expected restriction enzyme digestion patterns (Figure S21b). These results suggest that the 16 genes encoding hypothetical proteins in the phage S4 genome are non-essential for its replication and viability.

Following the successful construction of the three synthetic phages, we aimed to further simplify the phage S4 genome by simultaneously removing the first two clusters of genes encoding hypothetical proteins (ORF39-ORF43 and ORF44-ORF48) and, additionally, all three clusters (ORF39-ORF43, ORF44-ORF48, and ORF50-ORF55). To achieve this, we designed and assembled two additional yeast plasmids: pRSII313-S4-Δgp39–48 and pRSII313-S4-Δgp39–55. The assembly success rates were 2/16 and 3/8, respectively (Figures S22 and S23 and Table 1). The synthetic phage genomes were isolated from yeast and rebooted in *E. coli* NEB 10-beta. Plaque assay confirmed that phage S4-Δgp39–48 was successfully obtained (Figure 4a and Table 1). PCR verification of the resulting plaques confirmed the expected gene knockouts in the phage genome (Figure S24), and the synthetic phage exhibited the expected restriction enzyme digestion patterns (Figure S21b). However, when the synthetic phage genome with the three gene clusters removed was electroporated into *E. coli* NEB 10-beta, no plaques were observed. These results demonstrate that the genes in the ORF39-ORF48 are not essential for phage S4 survival and replication, and the ten unknown functional genes can be removed simultaneously to produce a synthetic phage S4-Δgp39–48 with a simplified genome featuring a knockout of 2883 bp.

To better understand the impact of the knockouts on phage antibacterial performance, the synthetic phages obtained were characterized and compared to the wild-type phage S4. All synthetic phages exhibited host-killing curves similar to that of the wild-type phage S4 (Figure 4b). This finding indicates that the genome knockouts did not compromise the antibacterial efficacy of the synthetic phages.

## 4. Discussion

In this study, using the newly isolated *Pseudomonas* phage S4 as a model, we demonstrated that functional headful packaging phages can be synthesized by yeast TAR cloning using only one-unit genome plus a terminally redundant sequence as short as 60 bp. This synthesis is independent of the length and position of the natural terminally redundant sequence. The discovery that only a 60-bp homologous arm is required for the release of the headful-packaged phage genome from the vector is noteworthy, as the next smallest reported sequence is 500 bp [23]. While genome simplification has been successfully reported in a range of organisms [44,45,46], simplifying phage genomes remains a challenge due to their compact genome structure, which contains a large number of unknown genes in phage genomes, and the instinct dependency of phage on host bacteria [44,47,48]. Our work demonstrates that total phage genome synthesis of phage offers a useful tool for knocking out clusters of unknown functional genes and facilitating further study of phage genome architecture and function. We would like to note that we observed two distinct peaks in the host killing curves for phage S4 [49]. This phenomenon may be dependent on either the host cell or the phage itself. We plan to investigate this hypothesis further in future studies.

We would like to point out that genome rebooting remains a significant challenge in phage synthesis. While we successfully rebooted the S4 genome in *E. coli* NEB 10-beta, our attempts to reboot the assembled genome of *P. aeruginosa* phage vB_PaeM_SCUT-S1 (S1) [32] in various *E. coli* strains, including NEB 10-beta, as well as in the cell-free TXTL system, failed to produce functional phages. These attempts included four yeast plasmids with terminal redundancies of 60 bp and 160 bp: pRSII313-S1-60, pYEP-II-S1-60, pRSII313-S1-160, and pYEP-II-S1-160, with assembly success rates of 7/16, 3/12, 8/16 and 2/10, respectively. We noticed, however, that when purified phage S1 DNA could be rebooted in its natural host, *P. aeruginosa* PAO1, the process yielded only a few plaques, indicating a low rebooting efficiency. Taken together, this underscores the need to expand the range of strains available for efficient phage genome rebooting.

The failure to delete all 16 unknown functional genes simultaneously suggests the presence of a synthetic lethal pair or pairs [44,50] between the genes in ORF44-ORF48 and ORF50-ORF55, which may collectively influence replication-related functions of phage S4. While the individual deletion of either ORF44-ORF48 or ORF50-ORF55 does not impede phage S4 replication, their simultaneous deletion may disrupt essential replication or propagation processes, resulting in the failure to reboot S4-Δgp39–55. The successful rebooting of the synthetic phage S4-Δgp39–48, contrasted with the inability to reboot S4-Δgp39–55, demonstrates that ORF39-ORF48 is not essential for phage S4 survival and replication in the presence of ORF50-ORF55.

To explore the functional significance of these deleted hypothetical genes, we conducted a BLASTP analysis of the 16 hypothetical proteins. The results indicated that their closest homologs are also annotated as hypothetical proteins (Table S5), highlighting the limited functional annotation available for these proteins. Additionally, using the online tools PromoterHunter [51] and ARNold [52], we analyzed the 16 hypothetical protein-coding genes to predict the presence of individual promoters and terminators (Table S5). The analysis revealed that six of the 16 ORFs (ORFs 39, 43, 44, 46, 48, and 55) possess both promoters and terminators, while five (ORFs 41, 42, 50, 51, and 54) lack these regulatory elements entirely. The remaining ORFs contain either a promoter or a terminator, but not both. These findings suggest that some of these genes may be expressed in tandem; however, further experimental validation is needed to confirm this hypothesis. To investigate these proteins further, we performed structural similarity comparisons with known proteins using AlphaFold3 [53] to predict protein functions (Table S6). The results indicate that these hypothetical proteins may play roles in facilitating phage infection and replication, potentially by modulating host immune responses or regulating host metabolism.

In summary, we have demonstrated that headful packaging phages can be synthesized, simplified, and engineered through the yeast TAR approach. With an expanding strain toolbox becoming available for rebooting, the potential of these phages in phage therapy will be increasingly explored.

## 5. Patents

Zhanglin Lin et al. A method for synthesizing Headful packaging bacteriophages. China, Patent No. CN202110398743.9, 17 August 2021.

## Figures and Tables

**Table 1:** Information on the synthetic phages.

Synthetic Phage	Assembled Yeast Plasmid	Designated First Nucleotide for the Synthesis of the Phage Genome	RFP	Assembly Success Rate	Number of Plaques
S4-a1	pRSII313-S4-a1	38,013	− ^1^	4/8	8
S4-b1	pYEP-II-S4-b1	38,013	−	5/7	>300
S4-a1-RFP	pRSII313-S4-a1-RFP	38,013	+ ^2^	5/8	>300
S4-b1-RFP	pYEP-II-S4-b1-RFP	38,013	+	12/16	55
S4-a2	pRSII313-S4-a2	32,386	−	5/8	>300
S4-b2	pYEP-II-S4-b2	32,386	−	8/8	>300
S4-a2-RFP	pRSII313-S4-a2-RFP	32,386	+	5/8	>300
S4-b2-RFP	pYEP-II-S4-b2-RFP	32,386	+	7/8	>300
S4-a3	pRSII313-S4-a3	11,858	−	3/8	45
S4-b3	pYEP-II-S4-b3	11,858	−	2/8	>300
S4-a3-RFP	pRSII313-S4-a3-RFP	11,858	−	5/8	50
S4-b3-RFP	pYEP-II-S4-b3-RFP	11,858	−	5/8	>300
S4-Δgp39–43	pRSII313-S4-Δgp39–43	32,386	−	2/16	>300
S4-Δgp44–48	pRSII313-S4-Δgp44–48	32,386	−	1/16	20
S4-Δgp50–55	pRSII313-S4-Δgp50–55	32,386	−	2/16	>300
S4-Δgp39–48	pRSII313-S4-Δgp39–48	32,386	−	2/16	11

^1^: without RFP; ^2^: with RFP.

## Data Availability

The data presented in this study are available in the article.

## Supplementary Materials

The following supporting information can be downloaded at: https://www.mdpi.com/article/10.3390/v17010045/s1, Figure S1. Rebooting of phage S4 genomic DNA. (a) Rebooting of phage S4 genomic DNA in *E. coli* NEB 10-beta. 200 ng of phage S4 genomic DNA was electroporated into *E. coli* NEB 10-beta cells for rebooting. The supernatants of the chloroform-treated *E. coli* were plated on a double layer agar plate with *P. aeruginosa* ATCC 9027 to observe plaque formation. (b) Infection of bacteria by phage S4. 10 μL of phage S4 lysates (107~109 PFU/mL) were spotted onto *P. aeruginosa* ATCC 9027 and *E. coli* NEB 10-beta, followed by overnight incubation at 37 °C to assess phage infectivity. Figure S2. Gene map of vector pYEP-II. Figure S3. Colony PCR verification of the plasmid pRSII313-S4-a0 assembled through yeast TAR. Lane “M” indicates DL 2000 DNA marker; lane numbers correspond to yeast transformant colonies. Figure S4. Colony PCR verification of the plasmid pYEP-II-S4-b0 assembled through yeast TAR. Lane “M” indicates DL 1000 DNA marker; lane numbers correspond to yeast transformant colonies. Figure S5. Colony PCR verification of the plasmids. (a) pRSII313-S4-a1 and (b) pYEP-II-S4-b1 assembled through yeast TAR. Lane “M” indicates DL 1000 DNA marker; lane numbers correspond to yeast transformant colonies. Figure S6. Colony PCR verification of the plasmid pRSII313-S4-a1-RFP assembled through yeast TAR. Lane “M” indicates DL 2000 DNA marker; lane numbers correspond to yeast transformant colonies. Figure S7. Colony PCR verification of the plasmid pYEP-II-S4-b1-RFP assembled through yeast TAR. Lane “M” indicates DL 2000 DNA marker; lane numbers correspond to yeast transformant colonies. Figure S8. Verification of the synthetic phages. (a) PCR verification of the RFP cassette. Lane “M” indicates DL 2000 DNA marker; and lane “P” shows the PCR products amplified from the phage S4 genomic DNA; Lane numbers correspond to PCR products amplified from the genomic DNA of either wild-type phage S4 or synthetic phages: 1: S4-a1, 2: S4-a1-RFP, 3: S4-b1, 4: S4-b1-RFP. (b) Restriction endonucleases digestion of phage DNA. SnaBI digestion of genomic DNA from the synthetic phages. Lane “M” contains the 1kb Extend DNA ladder, and lane “P” shows the digestion of wild-type phage S4 genomic DNA. Lane numbers correspond to the digestion products of synthetic phages: 1: S4-a1, 2: S4-a1-RFP, 3: S4-b1, 4: S4-b1-RFP. Figure S9. Sequencing results of junction PCR products between the first and last fragments in the synthetic phage genomes. Figure S10. Colony PCR verification of the plasmids (a) pRSII313-S4-a2 and (b) pYEP-II-S4-b2 assembled through yeast TAR. Lane “M” indicates DL 2000 DNA marker; lane numbers correspond to yeast transformant colonies. Figure S11. Colony PCR verification of the plasmid pRSII313-S4-a2-RFP assembled through yeast TAR. Lane “M” indicates DL 2000 DNA marker; lane numbers correspond to yeast transformant colonies. Figure S12. Colony PCR verification of the plasmid pYEP-II-S4-b2-RFP assembled through yeast TAR. Lane “M” indicates DL 2000 DNA marker; lane numbers correspond to yeast transformant colonies. Figure S13. Colony PCR verification of the plasmids (a) pRSII313-S4-a3 and (b) pYEP-II-S4-b3 assembled through yeast TAR. “M” indicates DL 2000 DNA marker; lane numbers correspond to yeast transformant colonies. Figure S14. Colony PCR verification of the plasmid pRSII313-S4-a3-RFP assembled through yeast TAR. “M” indicates DL 2000 DNA marker; lane numbers correspond to yeast transformant colonies. Figure S15. Colony PCR verification of the plasmid pYEP-II-S4-b3-RFP assembled through yeast TAR. “M” indicates DL 2000 DNA marker; lane numbers correspond to yeast transformant colonies. Figure S16. PCR verification and Sequencing analysis of the RFP-incorporated phages. (a) PCR verification the RFP expression cassette. Lane “M” indicates the DL 2000 DNA marker, and lane “P” shows the PCR products amplified from the phage S4 genomic DNA. Lane numbers correspond to PCR products amplified from the genomic DNA of either wild-type phage S4 or synthetic phages: 1: S4-a2, 2: S4-a2-RFP, 3: S4-b2, 4: S4-b2-RFP, 5: S4-a3, 6: S4-a3-RFP, 7: S4-b3, 8: S4-b3-RFP. (b) Sequencing results of the junction PCR products between the first and last fragments in the synthetic phage genomes. Figure S17. Restriction endonucleases digestion of phage DNA. EcoRI restriction digestion of phage DNA. Lane “M” contains the 1kb Extend DNA ladder, and lane “P” shows the template DNA from wild-type phage S4. Lane numbers correspond to template DNA from synthetic phages: 1: S4-a2, 2: S4-a2-RFP, 3: S4-b2, 4: S4-b2-RFP, 5: S4-a3, 6: S4-a3-RFP, 7: S4-b3, 8: S4-b3-RFP. The sizes of the DNA fragments are indicated. Figure S18. Colony PCR verification of the plasmid pRSII313-S4-Δgp39–43 assembled through yeast TAR. Lane “M” indicates DL 1000 DNA marker; lane numbers correspond to yeast transformant colonies. Figure S19. Colony PCR verification of the plasmid pRSII313-S4-Δgp44–48 assembled through yeast TAR. Lane “M” indicates DL 2000 DNA marker; lane numbers correspond to yeast transformant colonies. Figure S20. Colony PCR verification of the plasmid pRSII313-S4-Δgp50–55 assembled through yeast TAR. Lane “M” indicates DL 1000 DNA marker; lane numbers correspond to yeast transformant colonies. Figure S21. Verification of the synthetic phages with reduced genomes. (a) PCR verification of the synthetic phages with reduced genomes. Lane “M” indicates the DL 2000 DNA marker. Lane “S4” shows PCR products amplified from wild-type phage S4 genomic DNA. (a) Lane “P1” shows PCR products amplified from the yeast plasmid pRSII313-S4-Δgp39–43; lane numbers correspond to PCR products amplified from the genomic DNA of the synthetic phage S4-Δgp39–43. (b) Lane “P2” shows PCR products amplified from the yeast plasmid pRSII313-S4-Δgp44–48; lane numbers correspond to PCR products amplified from the genomic DNA of the synthetic phage -S4-Δgp44–48. (c) Lane “P3” shows PCR products amplified from the yeast plasmid pRSII313-S4-Δgp50–55; lane numbers correspond to PCR products amplified from the genomic DNA of the synthetic phage S4-Δgp50–55. (b) Restriction endonucleases digestion of phage DNA. EcoRI restriction digestion of phage DNA. Lane “M” contains the 1kb Extend DNA ladder. Lane “P” shows template DNA from wild-type phage S4. Lane numbers correspond to template DNA from synthetic phages:1: S4-Δgp39–43, 2: S4-Δgp44–48, 3: S4-Δgp50–55, 4: S4-Δgp39–48. The sizes of the DNA fragments are indicated. Figure S22. Colony PCR verification of the plasmid pRSII313-S4-Δgp39–48 assembled through yeast TAR. Lane “M” indicates DL 2000 DNA marker; lane numbers correspond to yeast transformant colonies. Figure S23. Colony PCR verification of the plasmid pRSII313-S4-Δgp39–55 assembled through yeast TAR. Lane “M” indicates DL 2000 DNA marker; lane numbers correspond to yeast transformant colonies. Figure S24. PCR verification of the synthetic phages with reduced genomes. Lane “M1” indicates the DL 2000 DNA marker, and lane “M2” indicates the DL 5000 DNA marker. Lane “S4” shows PCR products amplified from wild-type phage S4 genomic DNA. Lane “P” contains PCR products amplified from the yeast plasmid pRSII313-S4-Δgp39–48. Lane numbers correspond to PCR products amplified from the genomic DNA of the synthetic phage S4-Δgp39–48. Table S1: Summary of *Pseudomonas* phages based on DNA packaging modes. Table S2: Primers uesd for yeast TAR cloning. Table S3: Primers uesd for verification. Table S4: Genomic annotation of phage vB_Pae_SCUT-S4. Table S5: Predicted regulatory elements and the closest BLAST matches of the 16 hypothetical proteins. Table S6: Structural and functional predictions of the 16 hypothetical proteins using AlphaFold3.
