# Supplementary material for: Synthesis of Headful Packaging Phages Through Yeast Transformation-Associated Recombination
Source: Viruses. 2024 Dec 31;17(1):45. doi: 10.3390/v17010045 (PMC11769102; doi:10.3390/v17010045)
Supplement: Supplementary file 1 [file viruses-17-00045-s001.zip › Supplementary Figures.pdf]

Figure S1

a

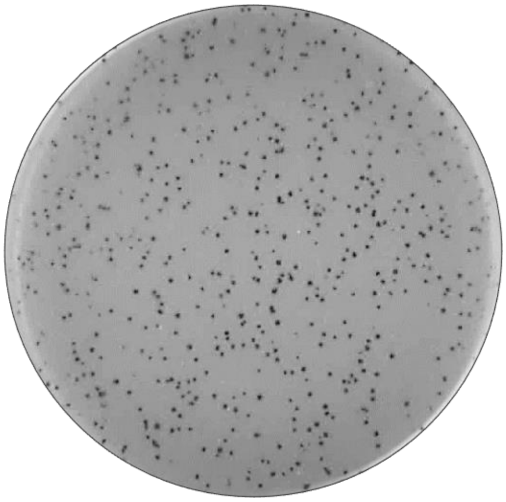

b

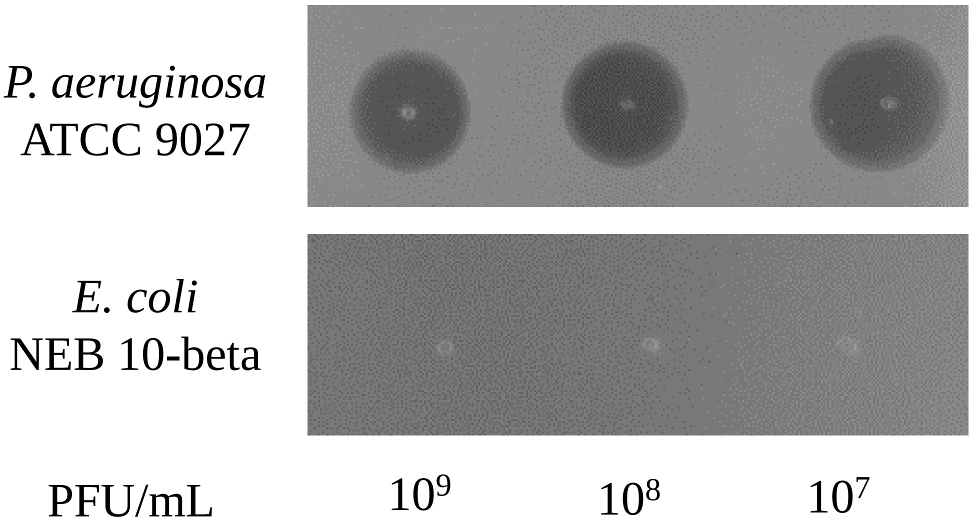

Figure S1. Rebooting of phage S4 genomic DNA.(a) Rebooting of phage S4 genomic DNA in E. coli NEB 10-beta. 200 ng of phage S4 genomic DNA was electroporated into *E. coli* NEB 10-beta cells for rebooting. The supernatants of the chloroform-treated *E. coli* were plated on a double layer agar plate with *P. aeruginosa* ATCC 9027 to observe plaque formation. (b) Infection of bacteria by phage S4. 10 μL of phage S4 lysates (10^7~10^9 PFU/mL) were spotted onto *P. aeruginosa* ATCC 9027 and *E. coli* NEB 10-beta, followed by overnight incubation at 37°C to assess phage infectivity.

Figure S2

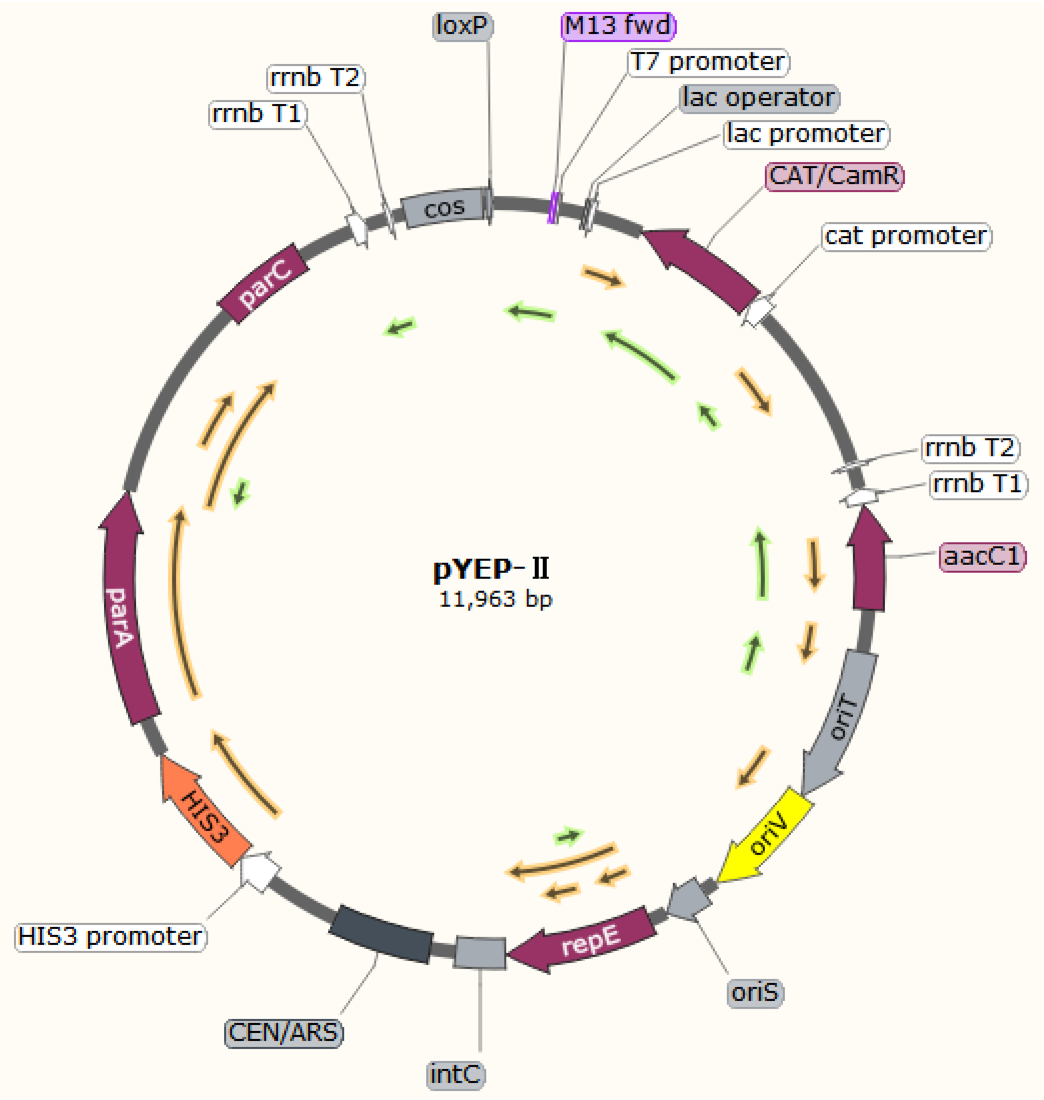

Figure S2. Gene map of vector pYEP-II.

Figure S3

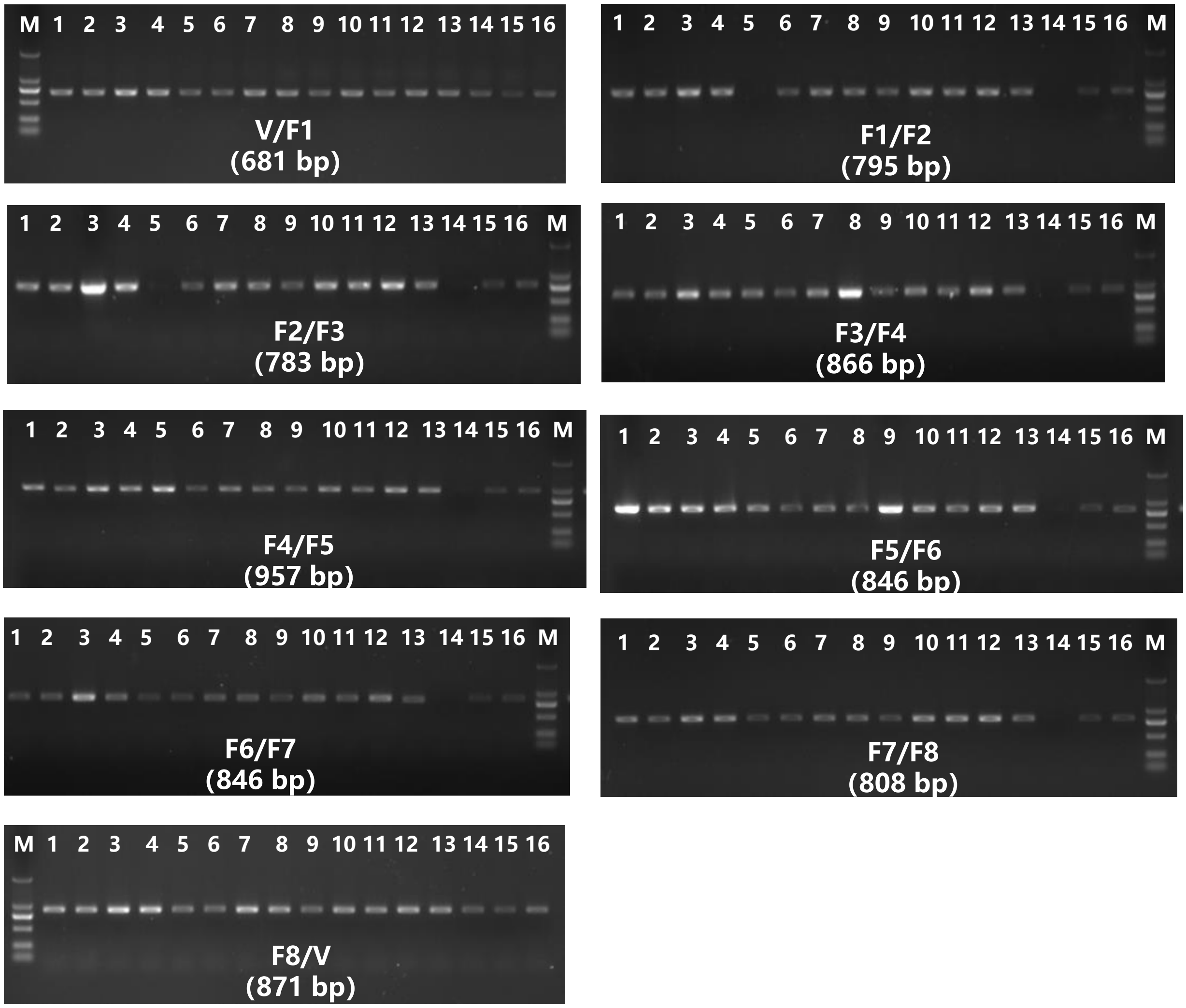

Figure S3. Colony PCR verification of the plasmid pRSII313-S4-a0 assembled through yeast TAR. Lane "M" indicates DL 2,000 DNA marker; lane numbers correspond to yeast transformant colonies.

Figure S4

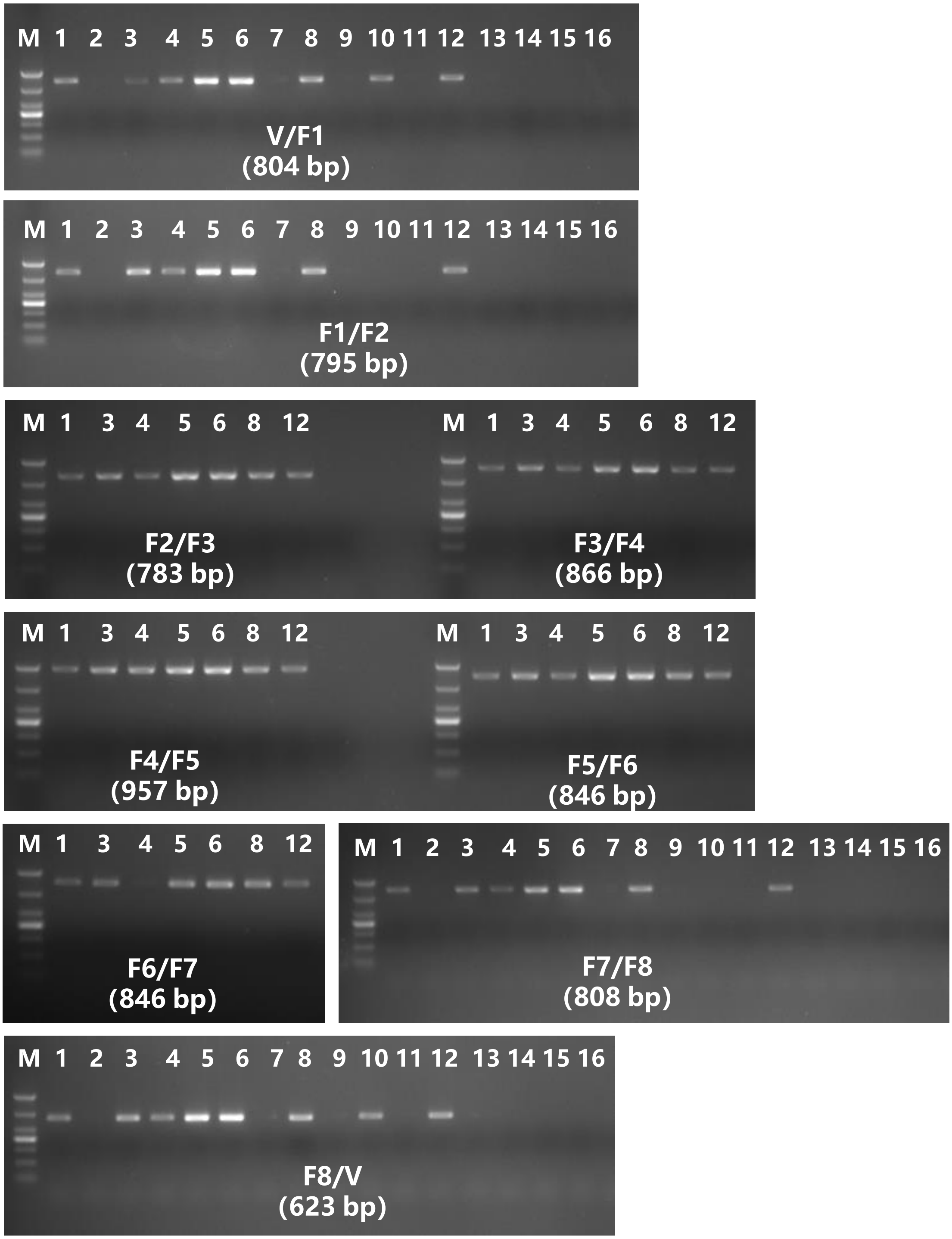

Figure S4. Colony PCR verification of the plasmid pYEP-II-S4-b0 assembled through yeast TAR. Lane "M" indicates DL 1,000 DNA marker; lane numbers correspond to yeast transformant colonies.

Figure S5

a

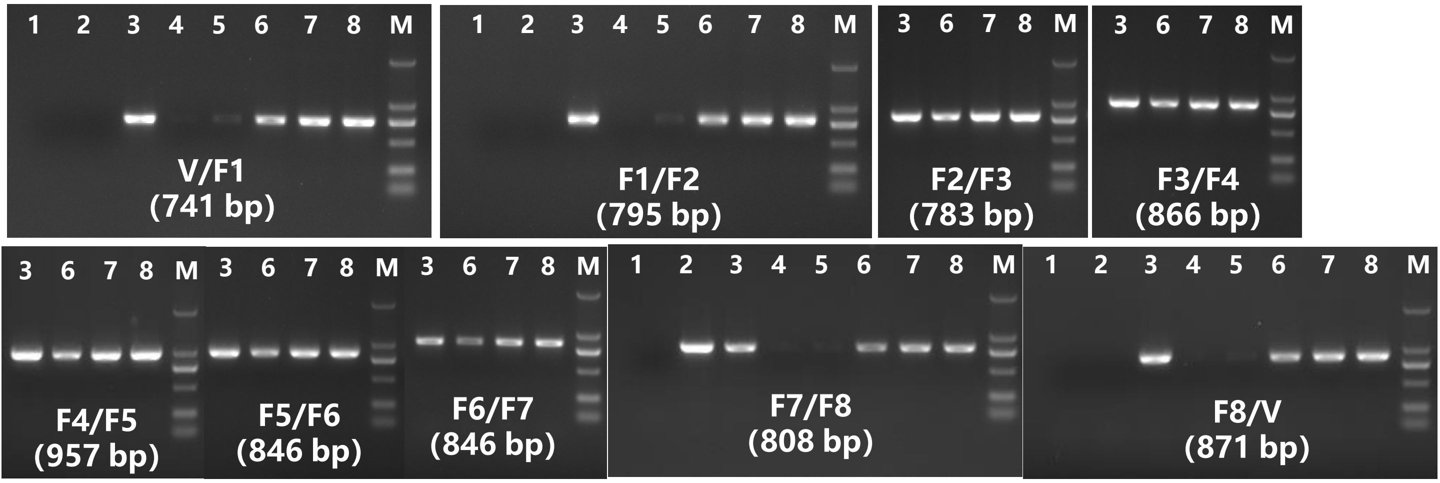

b

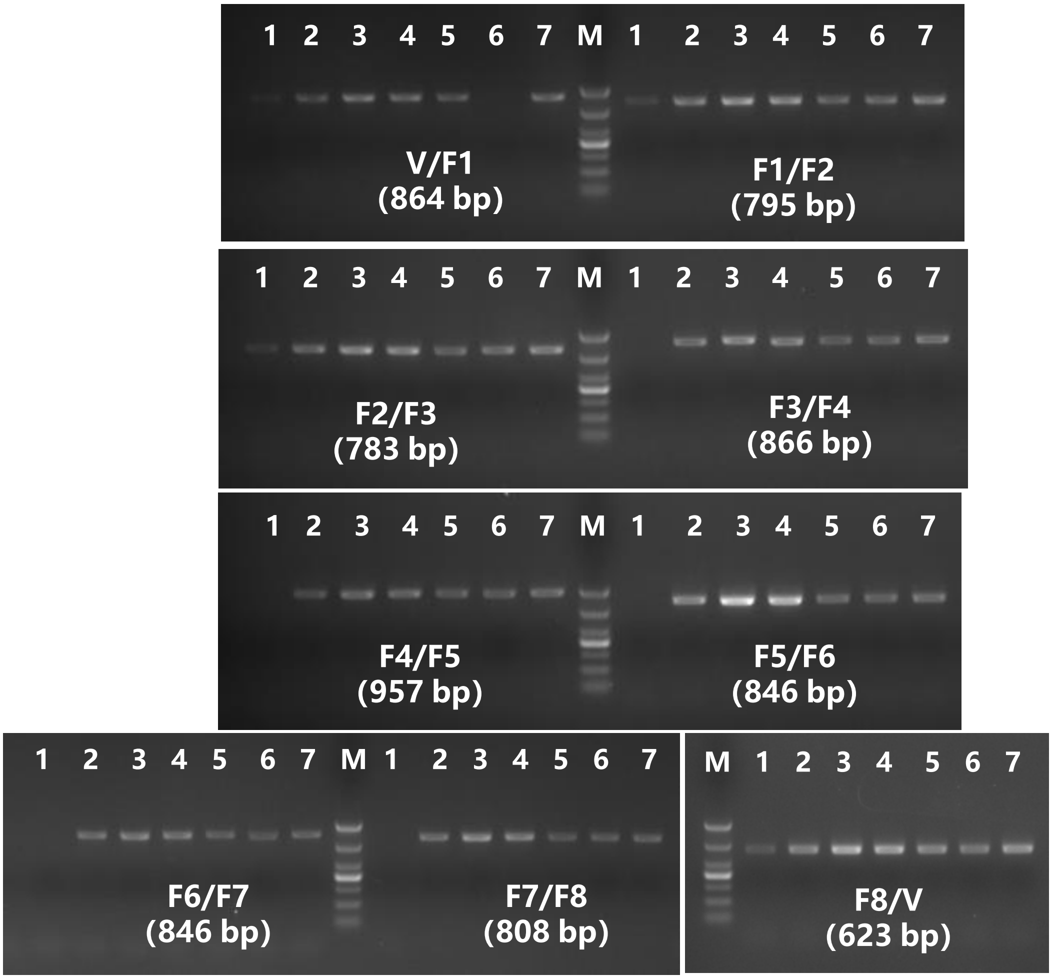

Figure S5. Colony PCR verification of the plasmids. (a) pRSII313-S4-a1 and (b) pYEP-II-S4-b1 assembled through yeast TAR. Lane "M" indicates DL 1,000 DNA marker; lane numbers correspond to yeast transformant colonies.

Figure S6

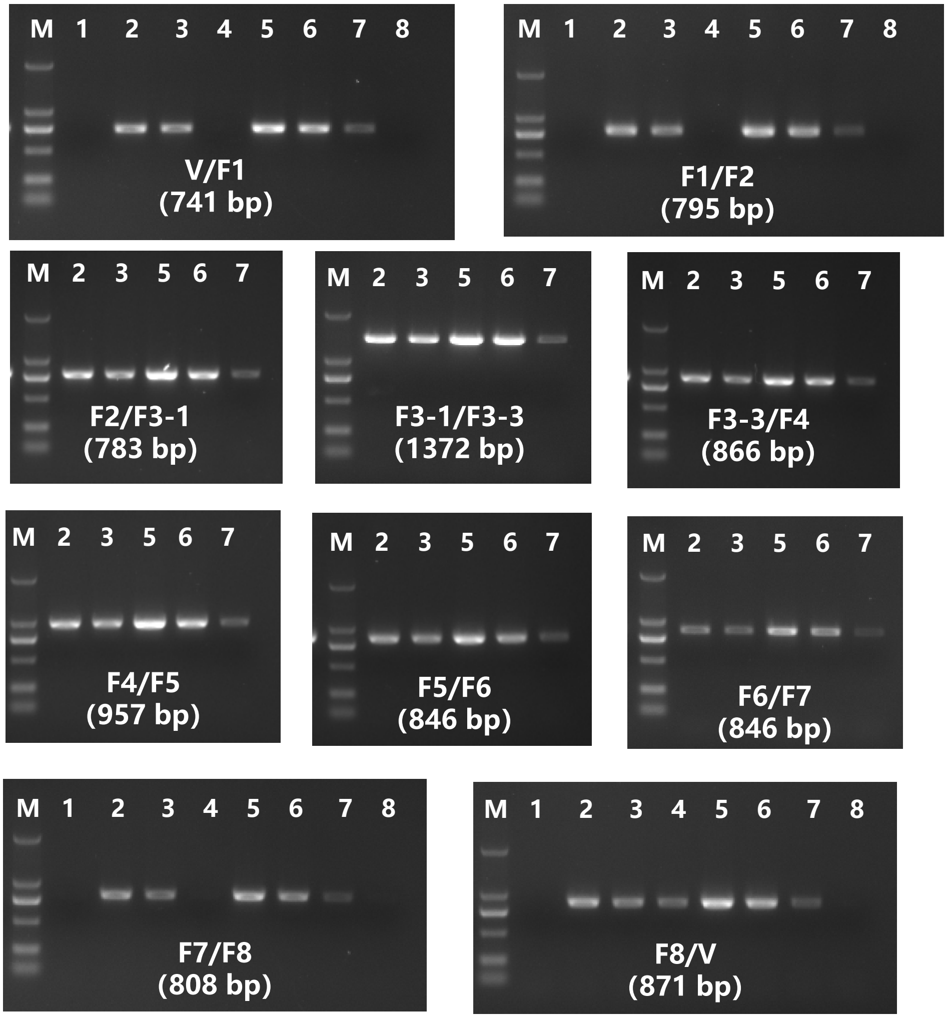

Figure S6. Colony PCR verification of the plasmid pRSII313-S4-a1-RFP assembled through yeast TAR. Lane "M" indicates DL 2,000 DNA marker; lane numbers correspond to yeast transformant colonies.

Figure S7

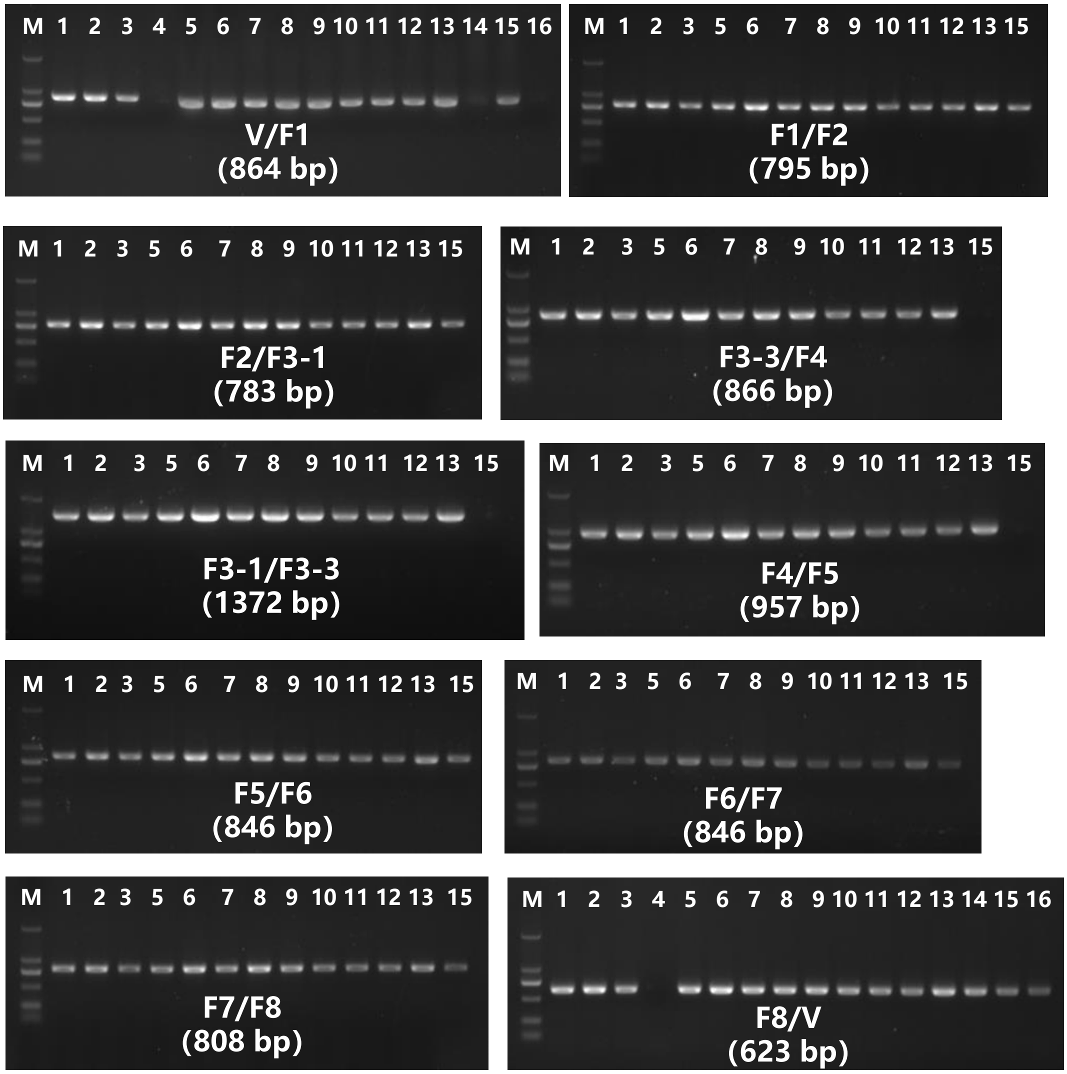

Figure S7. Colony PCR verification of the plasmid pYEP-II-S4-b1-RFP assembled through yeast TAR. Lane "M" indicates DL 2,000 DNA marker; lane numbers correspond to yeast transformant colonies.

Figure S8

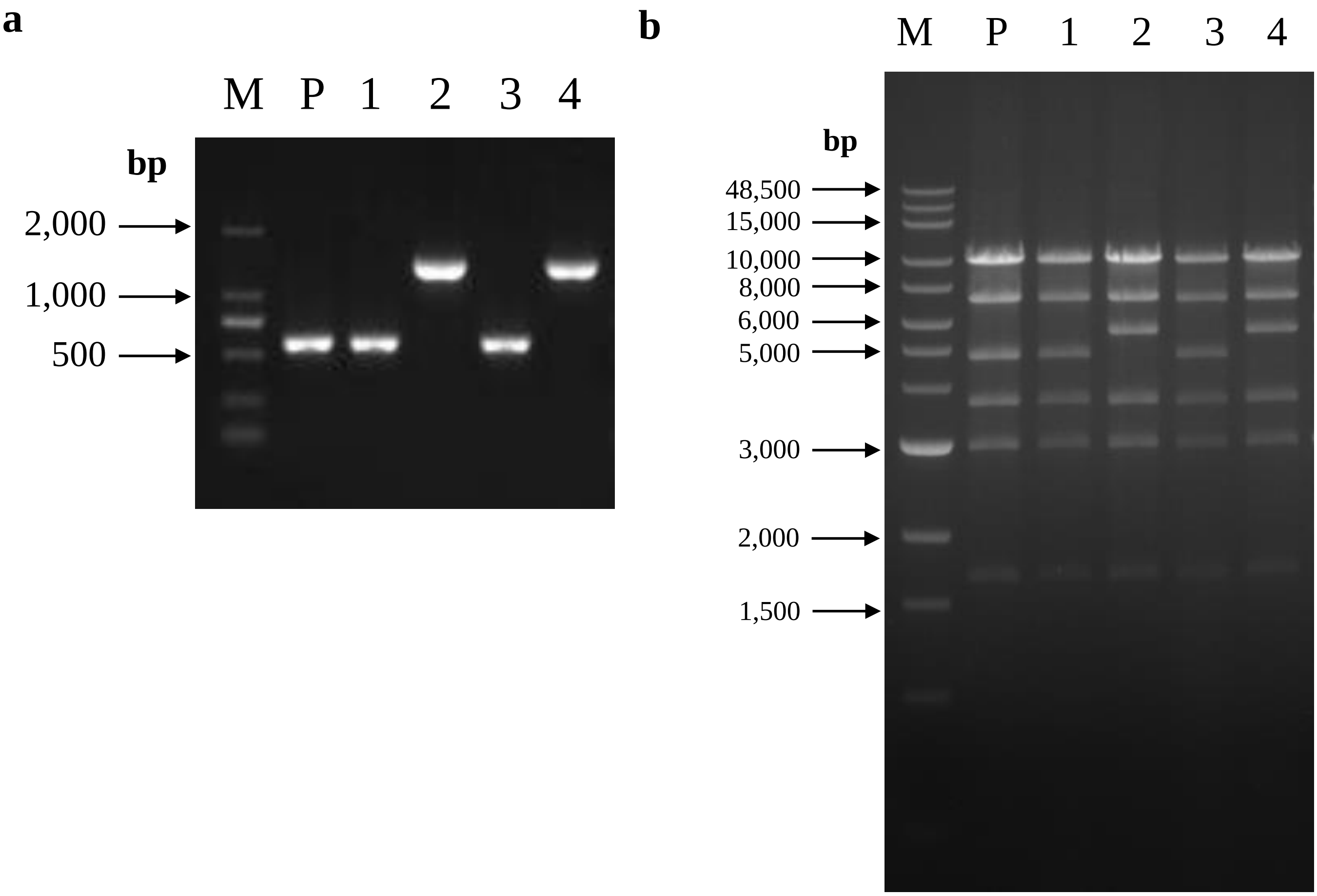

Figure S8. Verification of the synthetic phages. (a) PCR verification of the RFP cassette. Lane "M" indicates DL 2,000 DNA marker; and lane "P" shows the PCR products amplified from the phage S4 genomic DNA; Lane numbers correspond to PCR products amplified from the genomic DNA of either wild-type phage S4 or synthetic phages: 1: S4-a1, 2: S4-a1-RFP, 3: S4-b1, 4: S4-b1-RFP. (b) Restriction endonucleases digestion of phage DNA. SnaBI digestion of genomic DNA from the synthetic phages. Lane "M" contains the 1kb Extend DNA ladder, and lane "P" shows the digestion of wild-type phage S4 genomic DNA. Lane numbers correspond to the digestion products of synthetic phages: 1: S4-a1, 2: S4-a1-RFP, 3: S4-b1, 4: S4-b1-RFP.

Figure S9

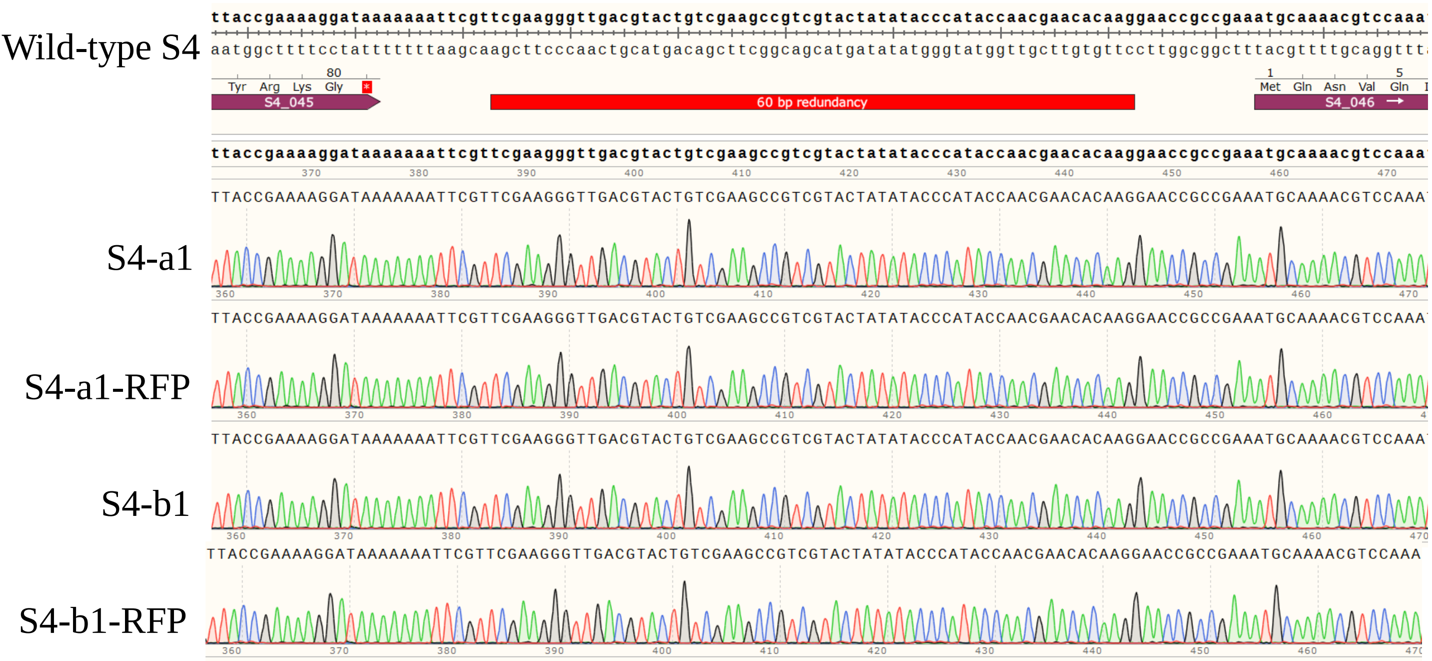

Figure S9. Sequencing results of junction PCR products between the first and last fragments in the synthetic phage genomes.

Figure S10

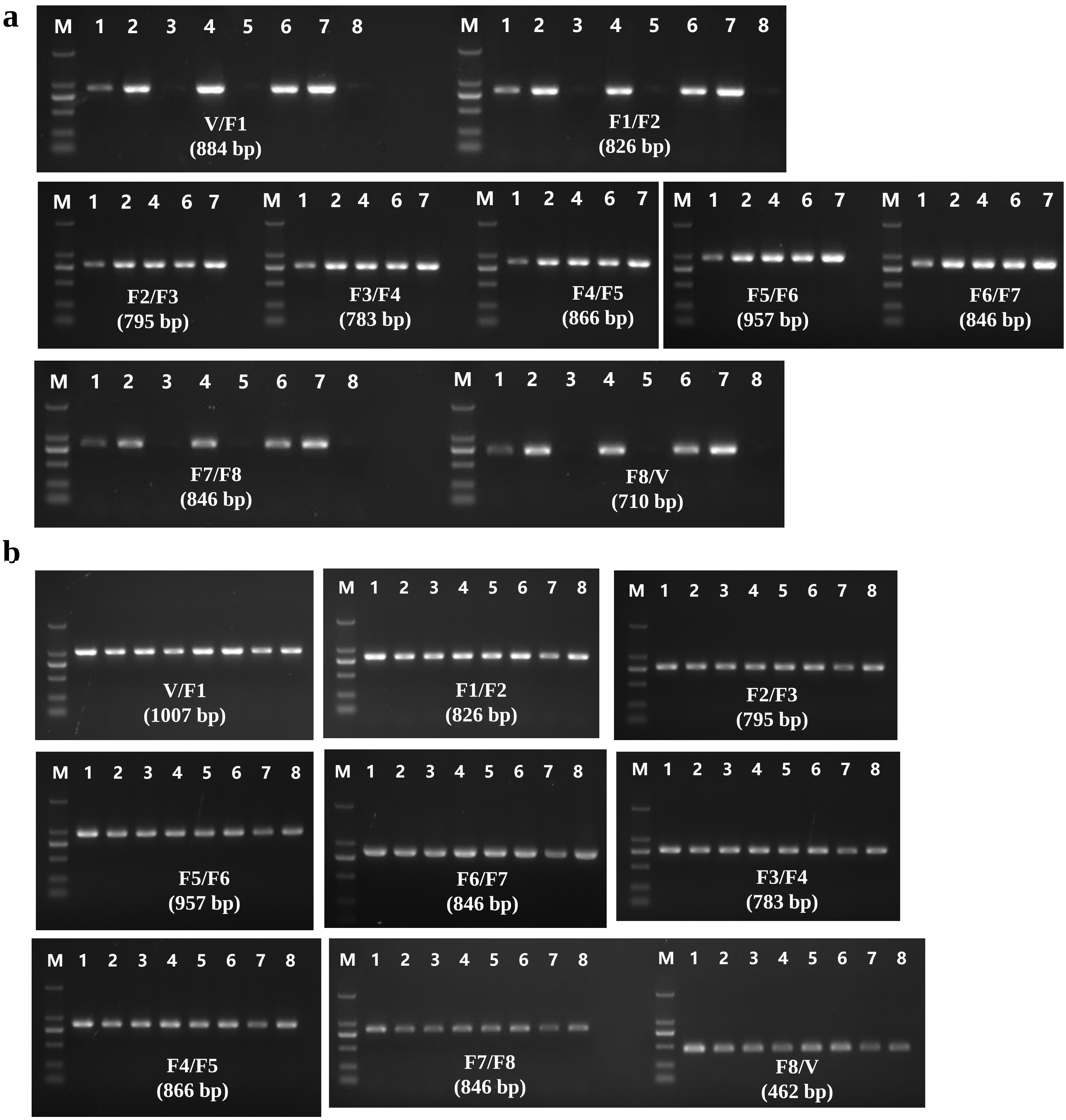

Figure S10. Colony PCR verification of the plasmids (a) pRSII313-S4-a2 and (b) pYEP-II-S4-b2 assembled through yeast TAR. Lane "M" indicates DL 2,000 DNA marker; lane numbers correspond to yeast transformant colonies.

Figure S11

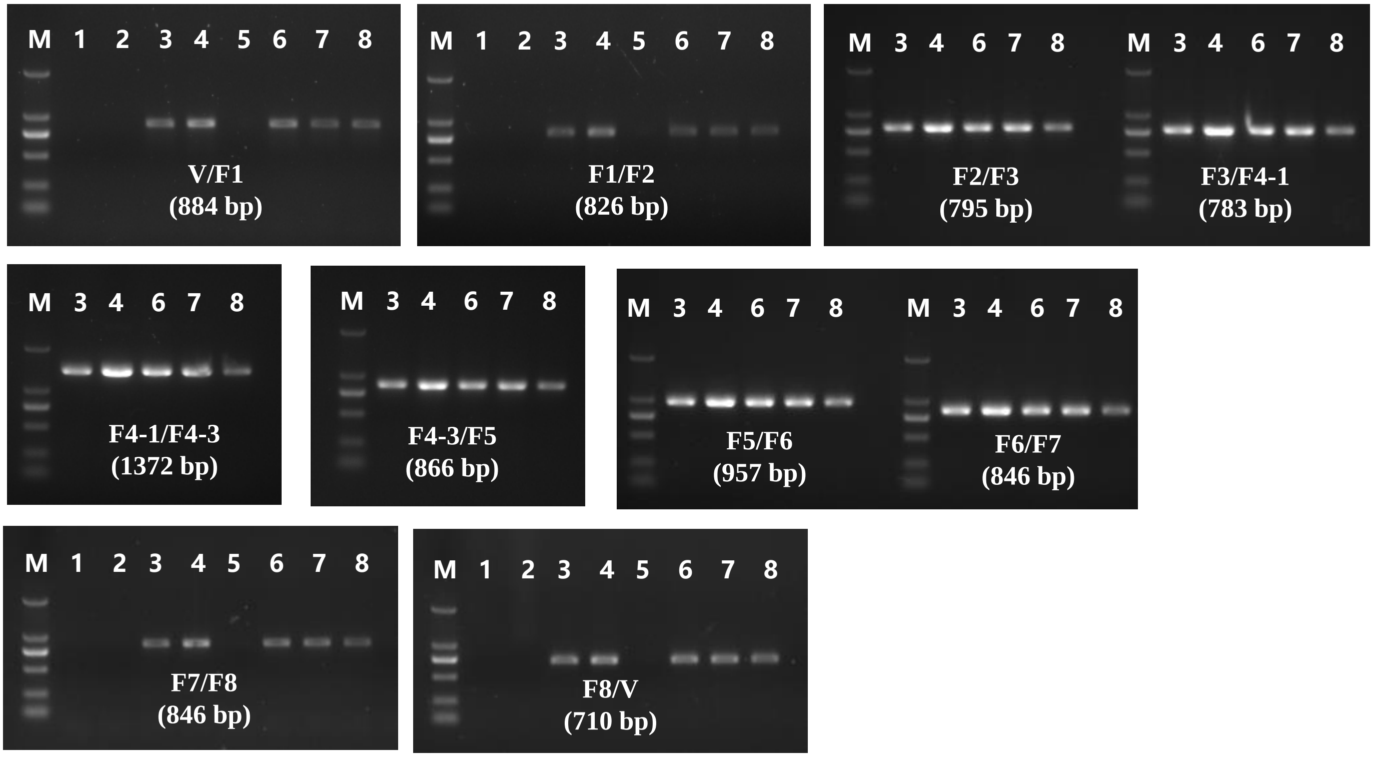

Figure S11. Colony PCR verification of the plasmid pRSII313-S4-a2-RFP assembled through yeast TAR. Lane "M" indicates DL 2,000 DNA marker; lane numbers correspond to yeast transformant colonies.

Figure S12

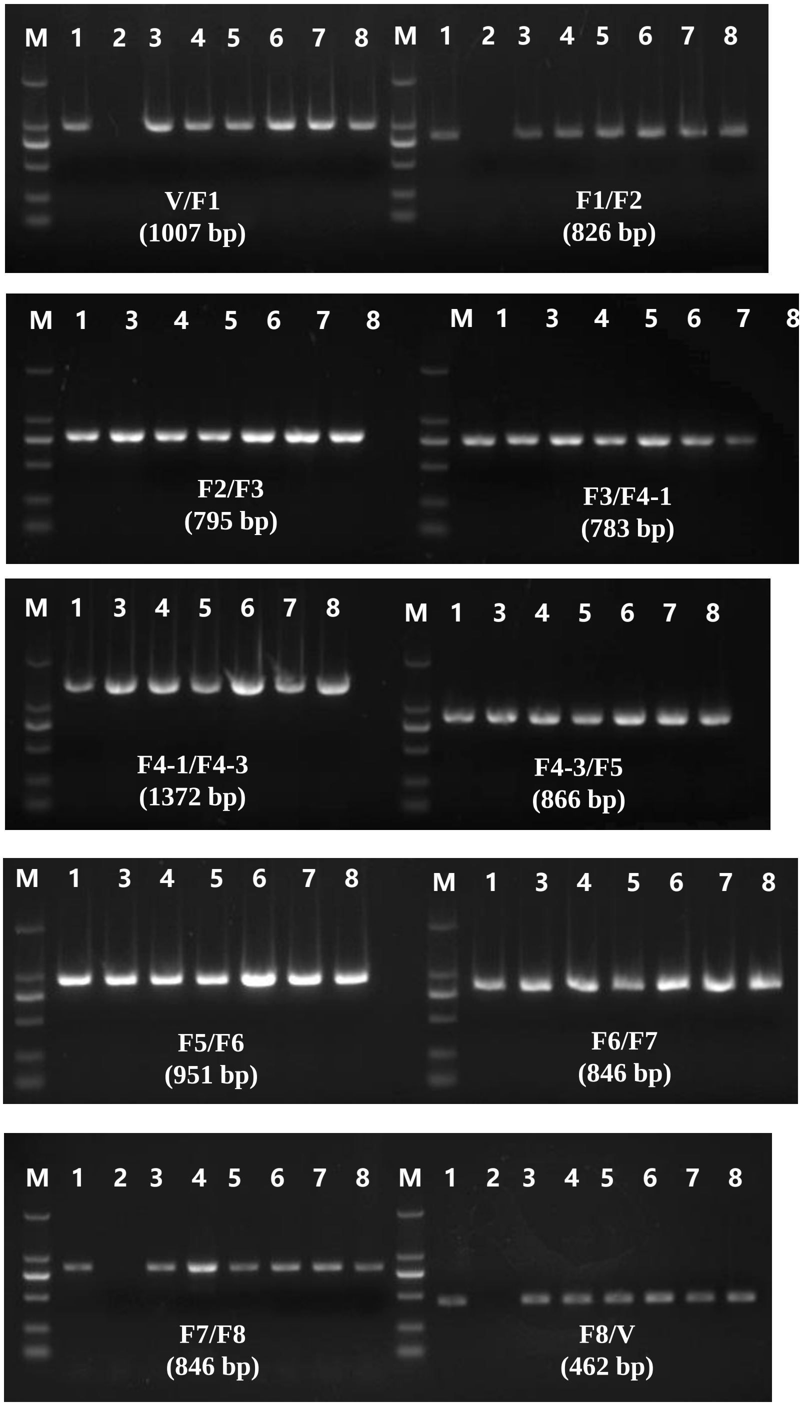

Figure S12. Colony PCR verification of the plasmid pYEP-II-S4-b2-RFP assembled through yeast TAR. Lane "M" indicates DL 2,000 DNA marker; lane numbers correspond to yeast transformant colonies.

Figure S13

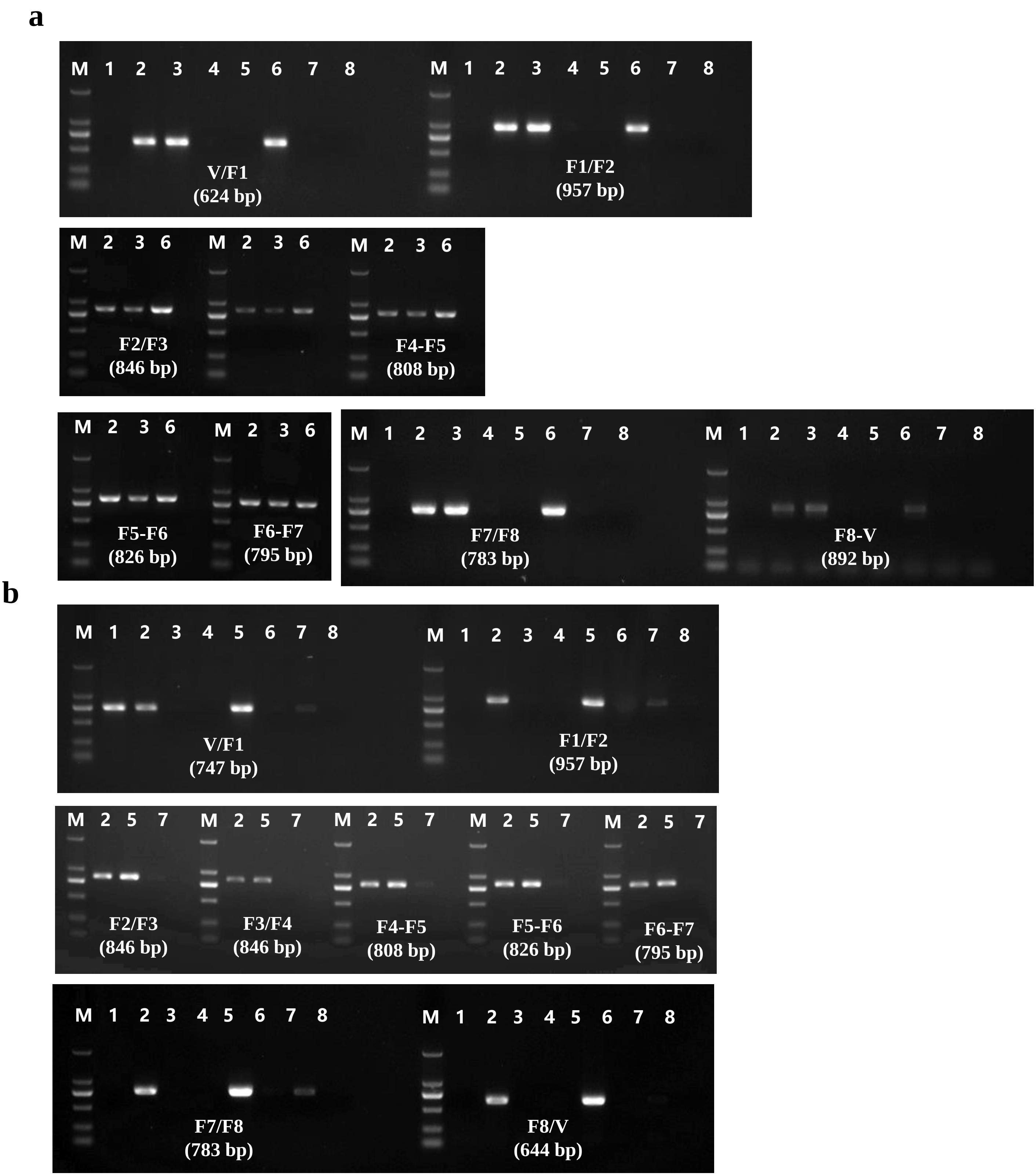

Figure S13. Colony PCR verification of the plasmids (a) pRSII313-S4-a3 and (b) pYEP-II-S4-b3 assembled through yeast TAR. "M" indicates DL 2,000 DNA marker; lane numbers correspond to yeast transformant colonies.

Figure S14

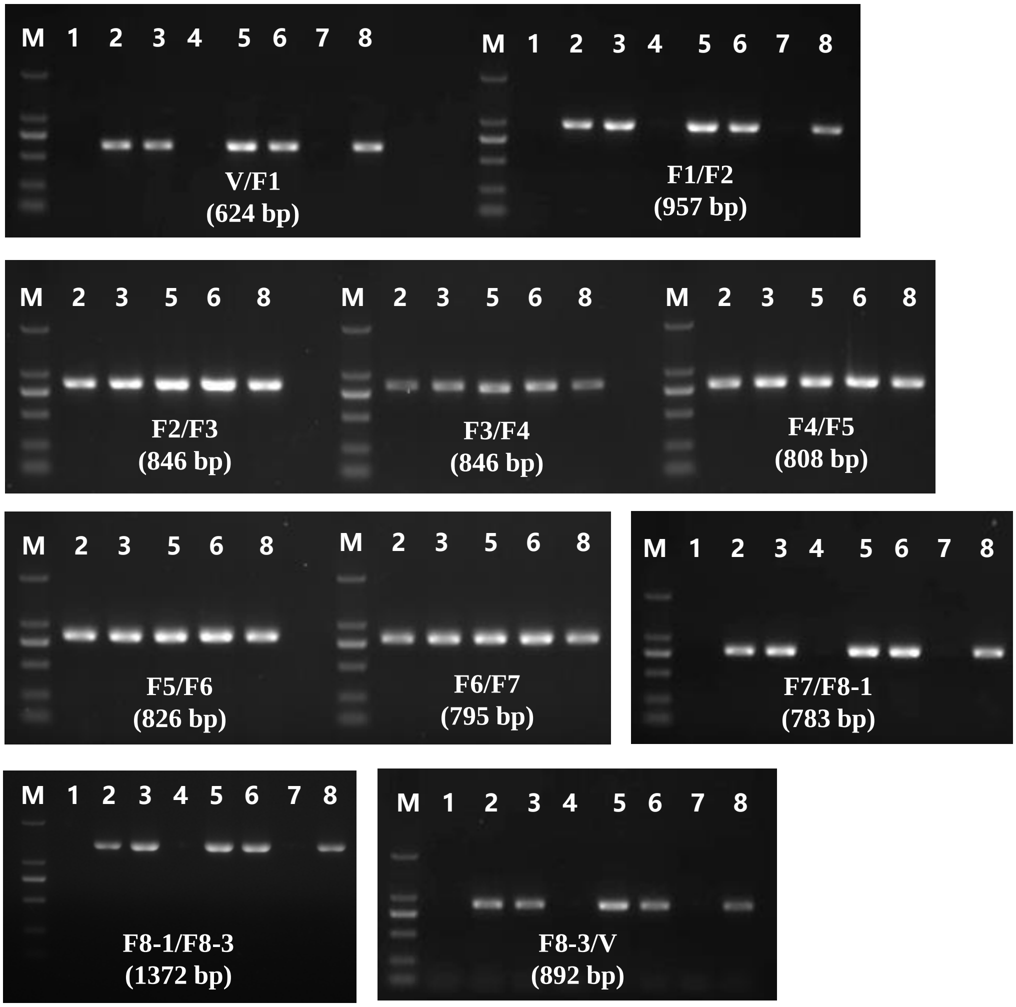

Figure S14. Colony PCR verification of the plasmid pRSII313-S4-a3-RFP assembled through yeast TAR. "M" indicates DL 2,000 DNA marker; lane numbers correspond to yeast transformant colonies.

Figure S15

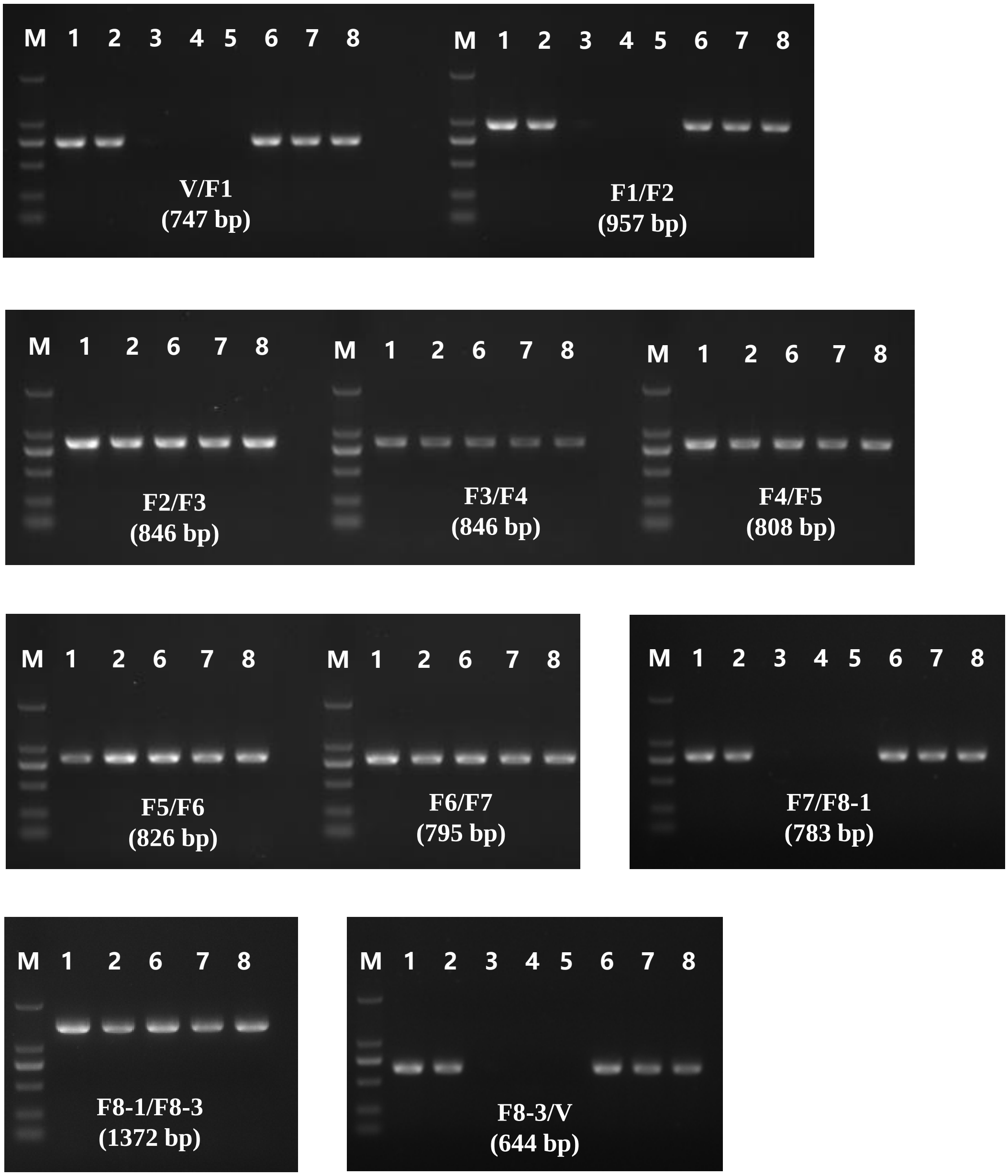

Figure S15. Colony PCR verification of the plasmid pYEP-II-S4-b3-RFP assembled through yeast TAR. "M" indicates DL 2,000 DNA marker; lane numbers correspond to yeast transformant colonies.

Figure S16

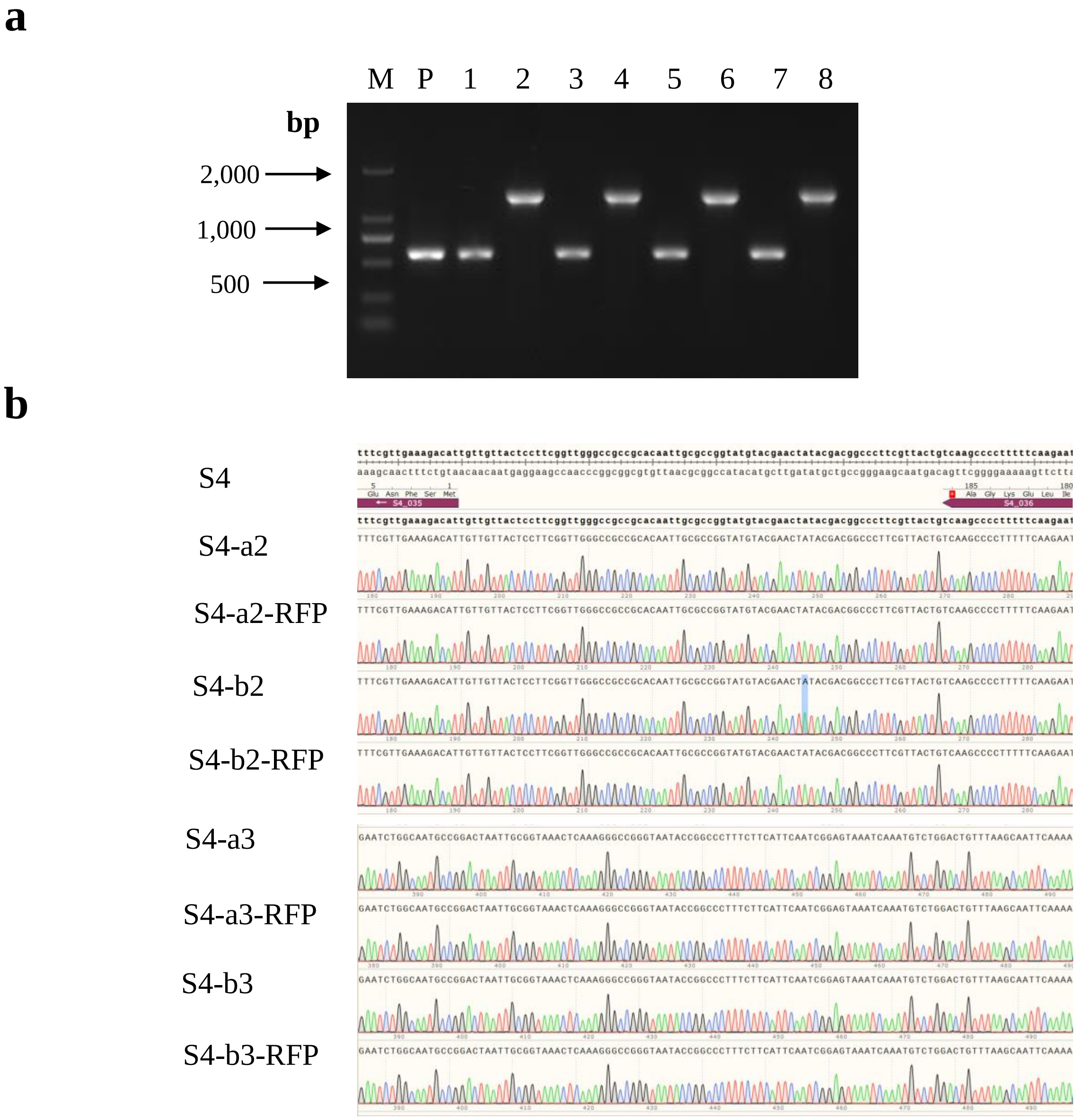

Figure S16. PCR verification and Sequencing analysis of the RFP-incorporated phages. (a) PCR verification the RFP expression cassette. Lane "M" indicates the DL 2,000 DNA marker, and lane "P" shows the PCR products amplified from the phage S4 genomic DNA. Lane numbers correspond to PCR products amplified from the genomic DNA of either wild-type phage S4 or synthetic phages: 1: S4-a2, 2: S4-a2-RFP, 3: S4-b2, 4: S4-b2-RFP, 5: S4-a3, 6: S4-a3-RFP, 7: S4-b3, 8: S4-b3-RFP. (b) Sequencing results of the junction PCR products between the first and last fragments in the synthetic phage genomes.

Figure S17

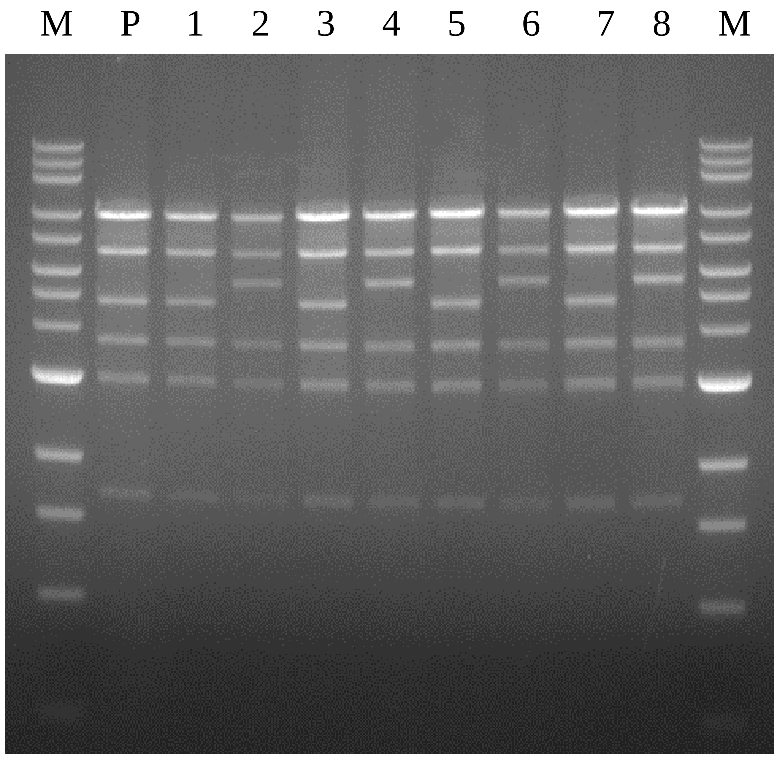

Figure S17. Restriction endonucleases digestion of phage DNA. EcoRI restriction digestion of phage DNA. Lane "M" contains the 1kb Extend DNA ladder, and lane "P" shows the template DNA from wild-type phage S4. Lane numbers correspond to template DNA from synthetic phages: 1: S4-a2, 2: S4-a2-RFP, 3: S4-b2, 4: S4-b2-RFP, 5: S4-a3, 6: S4-a3-RFP, 7: S4-b3, 8: S4-b3-RFP. The sizes of the DNA fragments are indicated.

Figure S18

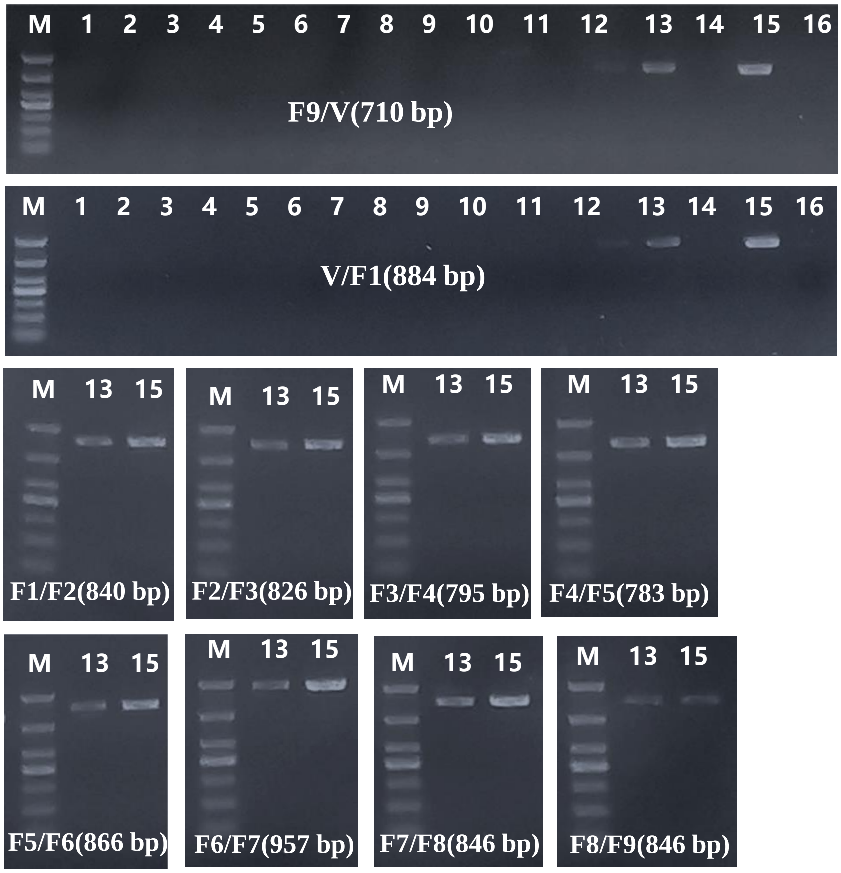

Figure S18. Colony PCR verification of the plasmid pRSII313-S4-Δgp39-43 assembled through yeast TAR. Lane "M" indicates DL 1,000 DNA marker; lane numbers correspond to yeast transformant colonies.

Figure S19

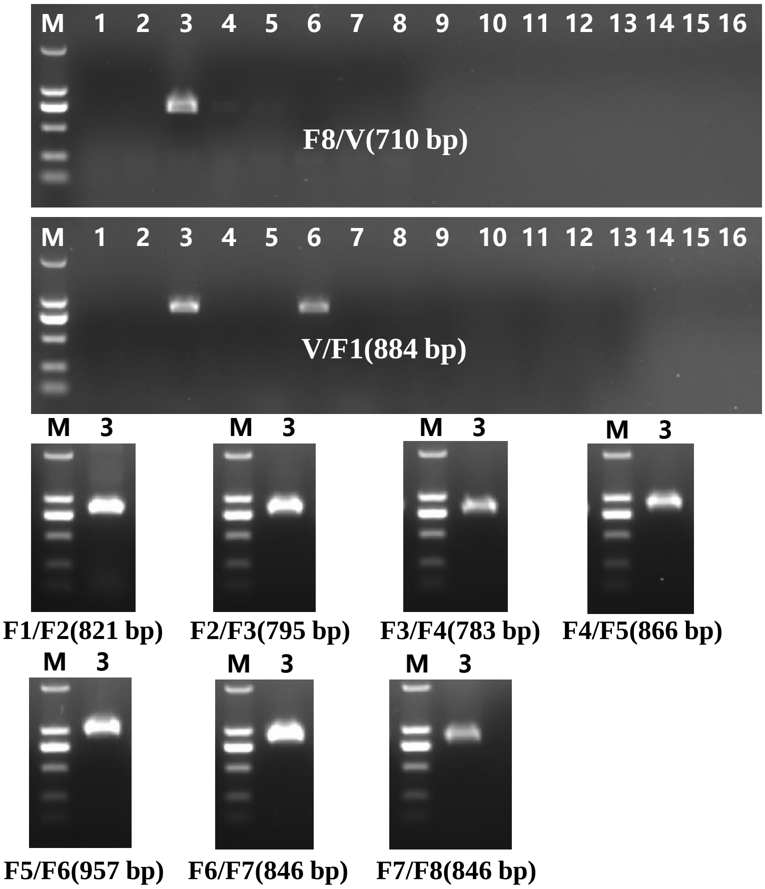

Figure S19. Colony PCR verification of the plasmid pRSII313-S4-Δgp44-48 assembled through yeast TAR. Lane "M" indicates DL 2,000 DNA marker; lane numbers correspond to yeast transformant colonies.

Figure S20

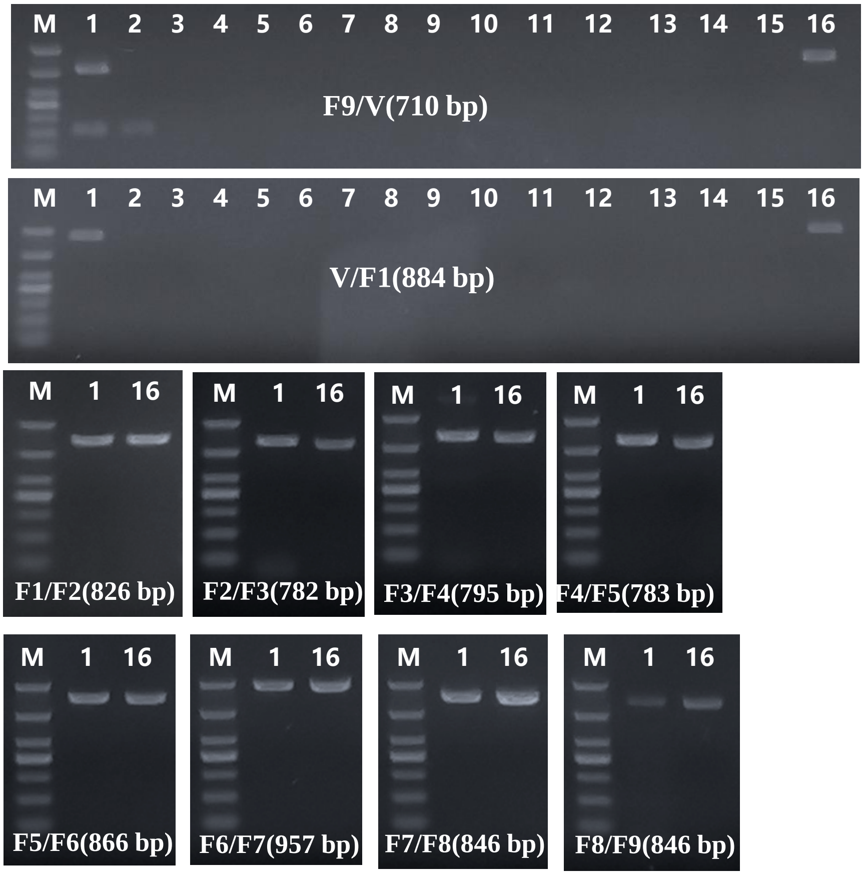

Figure S20. Colony PCR verification of the plasmid pRSII313-S4-Δgp50-55 assembled through yeast TAR. Lane "M" indicates DL 1,000 DNA marker; lane numbers correspond to yeast transformant colonies.

Figure S21

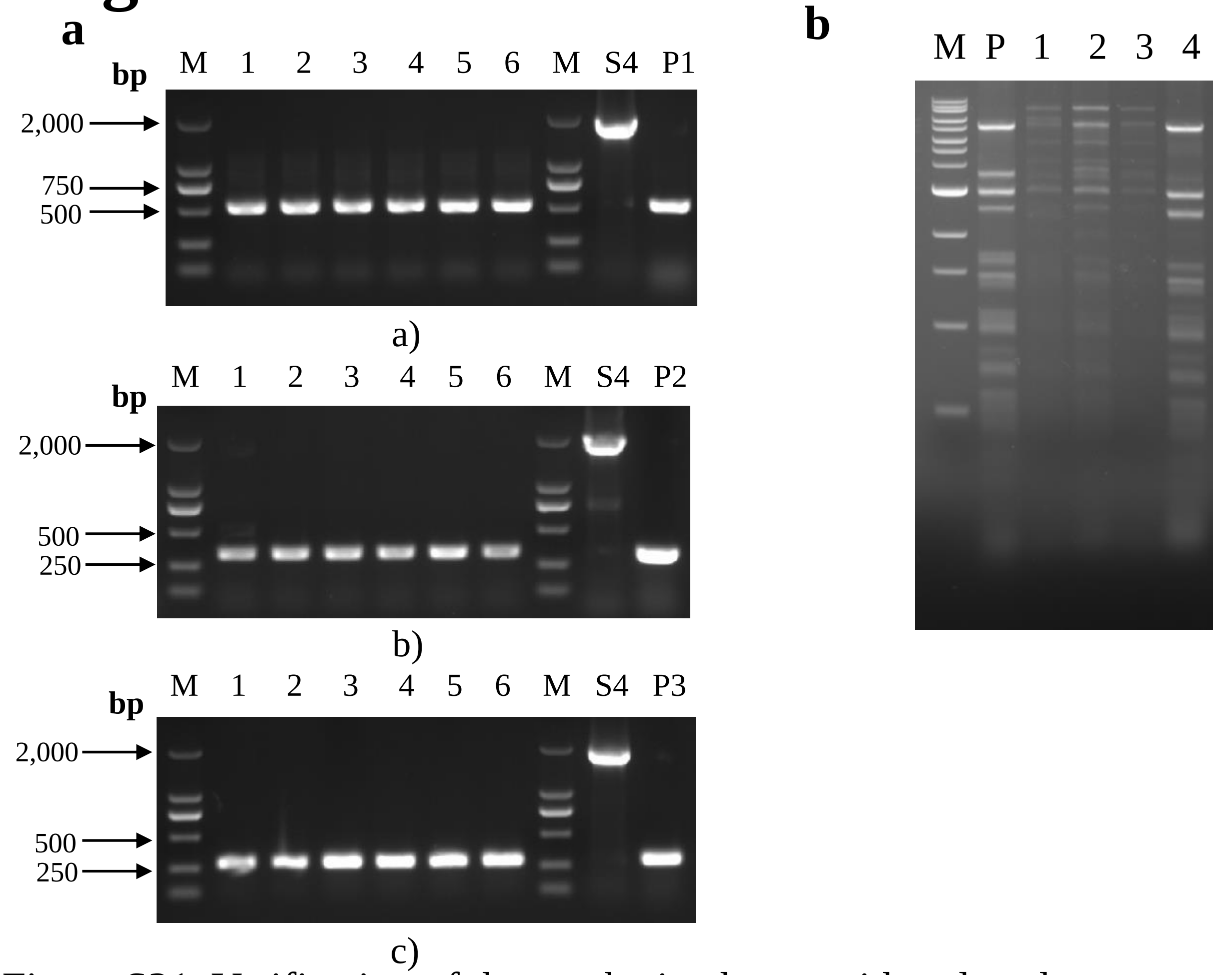

Figure S21. Verification of the synthetic phages with reduced genomes. (a) PCR verification of the synthetic phages with reduced genomes. Lane "M" indicates the DL 2,000 DNA marker. Lane "S4" shows PCR products amplified from wild-type phage S4 genomic DNA. a) Lane "P1" shows PCR products amplified from the yeast plasmid pRSII313-S4-Δgp39-43; lane numbers correspond to PCR products amplified from the genomic DNA of the synthetic phage S4-Δgp39-43. b) Lane "P2" shows PCR products amplified from the yeast plasmid pRSII313-S4-Δgp44-48; lane numbers correspond to PCR products amplified from the genomic DNA of the synthetic phage -S4-Δgp44-48. c) Lane "P3" shows PCR products amplified from the yeast plasmid pRSII313-S4-Δgp50-55; lane numbers correspond to PCR products amplified from the genomic DNA of the synthetic phage S4-Δgp50-55.(b) Restriction endonucleases digestion of phage DNA. EcoRI restriction digestion of phage DNA. Lane "M" contains the 1kb Extend DNA ladder. Lane "P" shows template DNA from wild-type phage S4. Lane numbers correspond to template DNA from synthetic phages:1: S4-Δgp39-43, 2: S4-Δgp44-48, 3: S4-Δgp50-55, 4: S4-Δgp39-48. The sizes of the DNA fragments are indicated.

Figure S22

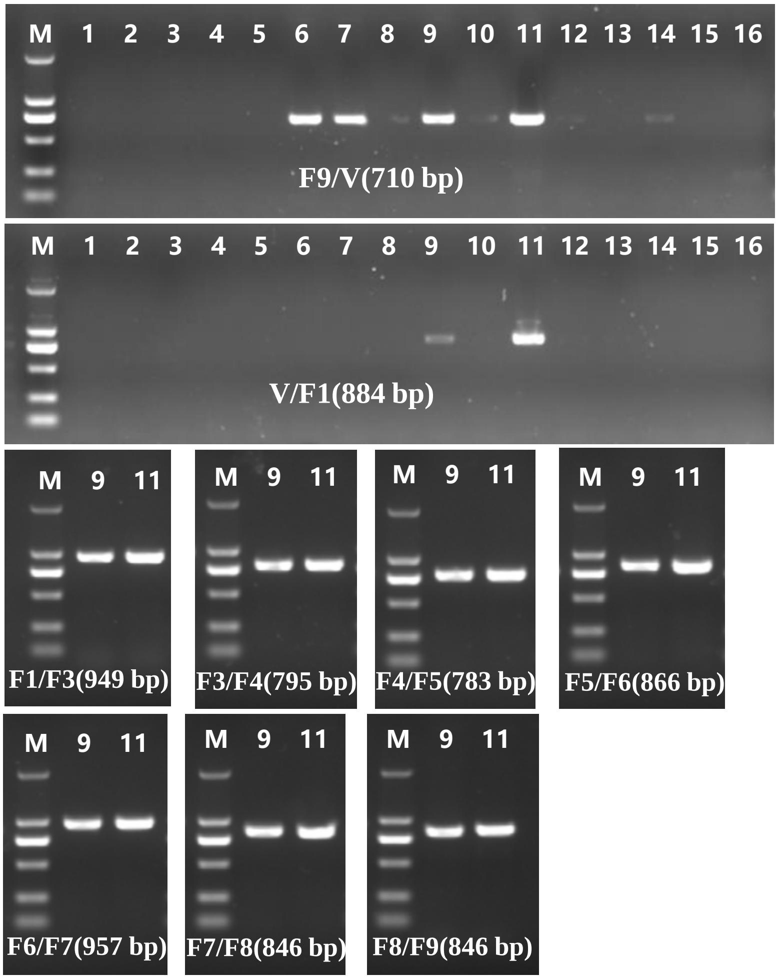

Figure S22. Colony PCR verification of the plasmid pRSII313-S4-△gp39-48 assembled through yeast TAR. Lane "M" indicates DL 2,000 DNA marker; lane numbers correspond to yeast transformant colonies.

Figure S23

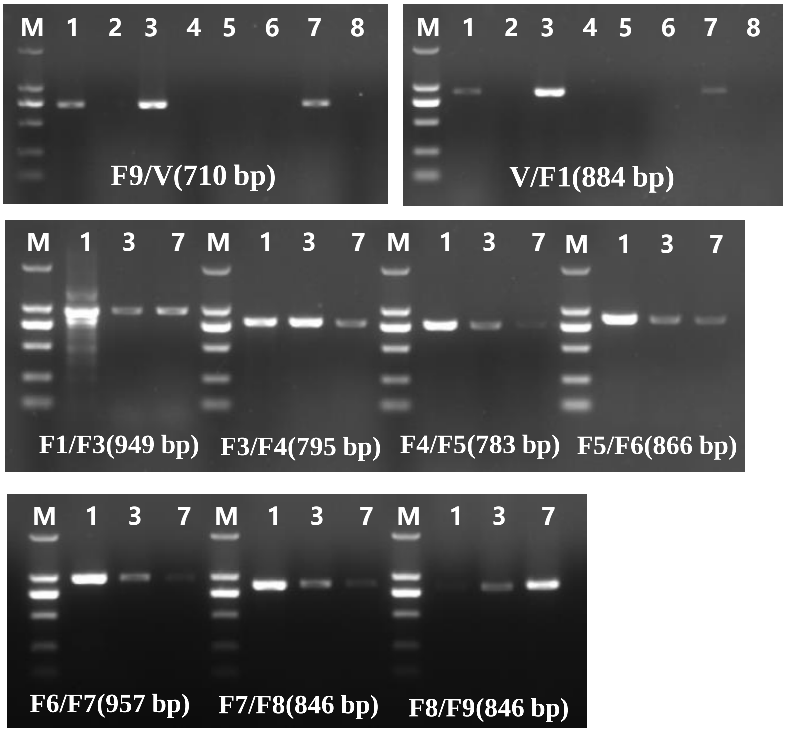

Figure S23. Colony PCR verification of the plasmid pRSII313-S4-Δgp39-55 assembled through yeast TAR. Lane "M" indicates DL 2,000 DNA marker; lane numbers correspond to yeast transformant colonies.

Figure S24

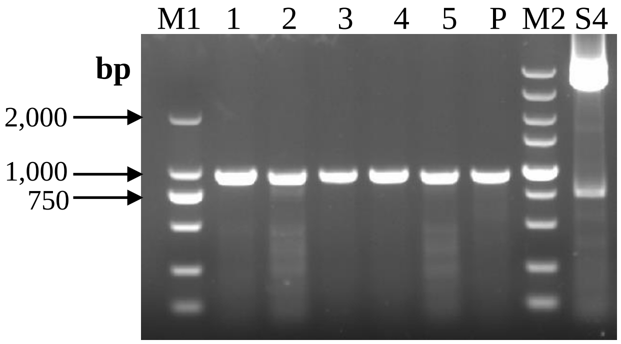

Figure S24. PCR verification of the synthetic phages with reduced genomes. Lane "M1" indicates the DL 2,000 DNA marker, and lane "M2" indicates the DL 5,000 DNA marker. Lane "S4" shows PCR products amplified from wild-type phage S4 genomic DNA. Lane "P" contains PCR products amplified from the yeast plasmid pRSII313-S4-Δgp39-48. Lane numbers correspond to PCR products amplified from the genomic DNA of the synthetic phage S4-Δgp39-48.
