# Supplementary material for: Synthesis of Headful Packaging Phages Through Yeast Transformation-Associated Recombination
Source: Viruses. 2024 Dec 31;17(1):45. doi: 10.3390/v17010045 (PMC11769102; doi:10.3390/v17010045)
Supplement: Supplementary file 1 [file viruses-17-00045-s001.zip › Supplementary Tables.pdf]

Table S1 Summary of *Pseudomonas* phages based on DNA packaging modes

Phage type	Percentage (%)
headful packaging	32.8%
DTR	35.8%
cos	2%
unknown	29.4%

Table S2 Primers uesd for yeast TAR cloning

Yeast plasmid	DNA fragments	Length of PCR products (bp)	Primer	5' → 3' sequence*	Description of yeast plasmid
pRSII313-S4-a0	Vector	4,981	HL20102-pRS313-F-S4	gtactatatacccataccaacgaacacaagGATATCAAGCTTATCGATACCGTCG	The plasmid contains unit-length genome from phage S4. The first and last genome fragments for TAR cloning are amplified with primers that carry ‘‘arms’’ that have homology with vector, and the first genome fragment has no homologous sequence to the last genome fragment. The first nucleotide of first genome fragment was located between ORF45 and ORF46. The plasmid pRSII313 was linearized with primer pairs HL20102-pRS313-F-S4 and HL20103-pRS313-R-S4 for TAR cloning vector.<br>Primer pairs:<br>HL20102-pRS313-F-S4 and HL20103-pRS313-R-S4<br>HL20100-S4-F1-313 and HL20052-TAR-S4-R<br>HL20051-TAR-S4-F and HL19194-S4-R1<br>HL20030-S4-F2 and HL20056-TAR-S4-R<br>HL20055-TAR-S4-F and HL19196-S4-R2<br>HL20031-S4-F3 and HL20058-TAR-S4-R<br>HL20057-TAR-S4-F and HL19198-S4-R3<br>HL20032-S4-F4 and HL20054-TAR-S4-R<br>HL20053-TAR-S4-F and HL20101-S4-R8-313
			HL20103-pRS313-R-S4	tgatttggacgttttgcatttcggcggttcCACCGCGGTGGAGCTCCAATTC	
	F1	5,416	HL20100-S4-F1-313	TATAGGGCGAATTGGAGCTCCACCGCGGTGgaaccgccgaaatgcaaaacg	
			HL20052-TAR-S4-R	acgatggaaagcgcgaaaaca	
	F2	4,837	HL20051-TAR-S4-F	gtcgattccgccgcagtatatg	
			HL19194-S4-R1	gcttattgtccgatagtgccatgat	
	F3	6,007	HL20030-S4-F2	ggcgataatgtgggcaacaacg	
			HL20056-TAR-S4-R	tgatacgaacgacgcttgataagtg	
	F4	5,751	HL20055-TAR-S4-F	cgtcggaactgttatcaagaacg	
			HL19196-S4-R2	aatcaattcgcggtcaagtcc	
	F5	5,206	HL20031-S4-F3	gaagcgaacggatggacgac	
			HL20058-TAR-S4-R	cggtaatcgcagattgaacaataagc	
	F6	5,138	HL20057-TAR-S4-F	cgcaaacgctgtataacgaagg	
			HL19198-S4-R3	agtatcccgaagttctttccgc	
	F7	5,600	HL20032-S4-F4	tttatcgggcgatgtttcggc	
			HL20054-TAR-S4-R	gctttcgacgctgttaacgc	
	F8	5,457	HL20053-TAR-S4-F	aagcgcatcgtaccaaaagc	
			HL20101-S4-R8-313	GAGGTCGACGGTATCGATAAGCTTGATATCcttgtgttcgttggtatgggtat	
pYEP-II-S4-b0	F1	5,452	HL20069-TAR-S4-F1-1	CGGGGATCCTCTAGAGTCGACCTGCAGCTCGAGgaaccgccgaaatgcaaaacg	The plasmid contains unit-length genome from phage S4. The first and last genome

			HL20070-S4-TAR-F1-2	GACTCACTATAGGGCGAATTCGAGCTCGGTACCCGGGGATCCTCTAGAGTCGAC	fragments for TAR cloning are amplified with primers that carry ‘‘arms’’ that have homology with vector, and the first genome fragment has no homologous sequence to the last genome fragment. The first nucleotide of first genome fragment was located between ORF45 and ORF46. The plasmid pYEP-II was digested by SphI and HindIII for TAR cloning vector.<br>Primer pairs:<br>HL20069-TAR-S4-F1-1 and HL20052-TAR-S4-R<br>HL20070-S4-TAR-F1-2 and HL20052-TAR-S4-R<br>HL20051-TAR-S4-F and HL19194-S4-R1<br>HL20030-S4-F2 and HL20056-TAR-S4-R<br>HL20055-TAR-S4-F and HL19196-S4-R2<br>HL20031-S4-F3 and HL20058-TAR-S4-R<br>HL20057-TAR-S4-F and HL19198-S4-R3<br>HL20032-S4-F4 and HL20054-TAR-S4-R<br>HL20053-TAR-S4-F and HL20035-S4-F4-R1<br>HL20053-TAR-S4-F and HL20036-S4-F4-R2
			HL20052-TAR-S4-R	acgatggaaagcgcgaaaaca	
	F2	4,837	HL20051-TAR-S4-F	gtcgattccgccgcagtatatg	
			HL19194-S4-R1	gcttattgtccgatagtgccatgat	
	F3	6,007	HL20030-S4-F2	ggcgataatgtgggcaacaacg	
			HL20056-TAR-S4-R	tgatacgaacgacgcttgataagtg	
	F4	5,751	HL20055-TAR-S4-F	cgtcggaactgttatcaagaacg	
			HL19196-S4-R2	aatcaattcgcggtcaagtcc	
	F5	5,206	HL20031-S4-F3	gaagcgaacggatggacgac	
			HL20058-TAR-S4-R	cggtaatcgcagattgaacaataagc	
	F6	5,138	HL20057-TAR-S4-F	cgcaaacgctgtataacgaagg	
			HL19198-S4-R3	agtatcccgaagttctttccgc	
	F7	5,600	HL20032-S4-F4	tttatcgggcgatgtttcggc	
			HL20054-TAR-S4-R	gctttcgacgctgttaacgc	
	F8	5,493	HL20053-TAR-S4-F	aagcgcatcgtaccaaaagc	
			HL20035-S4-F4-R1	AGCTATTTAGGTGACACTATAGAATACTCCTCGAGcttgtgttcgttggtatgggtata	
			HL20036-S4-F4-R2	ACAGGAAACAGCTATGACCATGATTACGCCAAGCTATTTAGGTGACACTATAGAATACT	
pRSII313-S4-a1	Vector	4,981	HL20102-pRS313-F-S4	gtactatatacccataccaacgaacacaagGATATCAAGCTTATCGATACCGTCG	The plasmid contains unit-length genome and 60bp circularly permuted sequence from phage S4. The first and last genome fragments for TAR cloning are amplified with primers that carry ‘‘arms’’ that have homology with vector, and the first genome fragment has 60bp homologous sequence to the last genome fragment. The first nucleotide of first genome fragment was
			HL20132-pRS313-S4-R	gacggcttcgacagtacgtcaacccttcgaCACCGCGGTGGAGCTCCAATTC	
	F1	5,476	HL20131-S4-F1-313	TATAGGGCGAATTGGAGCTCCACCGCGGTGtcgaagggttgacgtactgtcg	
			HL20052-TAR-S4-R	acgatggaaagcgcgaaaaca	
	F2	4,837	HL20051-TAR-S4-F	gtcgattccgccgcagtatatg	
			HL19194-S4-R1	gcttattgtccgatagtgccatgat	

	F3	6,007	HL20030-S4-F2	ggcgataatgtgggcaacaacg	located between ORF45 and ORF46. The plasmid pRSII313 was linearized with primer pairs HL20102-pRS313-F-S4 and HL20132-pRS313-S4-R for TAR cloning vector.<br>Primer pairs:<br>HL20102-pRS313-F-S4 and HL20132-pRS313-S4-R<br>HL20131-S4-F1-313 and HL20052-TAR-S4-R<br>HL20051-TAR-S4-F and HL19194-S4-R1<br>HL20030-S4-F2 and HL20056-TAR-S4-R<br>HL20055-TAR-S4-F and HL19196-S4-R2<br>HL20031-S4-F3 and HL20058-TAR-S4-R<br>HL20057-TAR-S4-F and HL19198-S4-R3<br>HL20032-S4-F4 and HL20054-TAR-S4-R<br>HL20053-TAR-S4-F and HL20101-S4-R8-313
			HL20056-TAR-S4-R	tgatacgaacgacgcttgataagtg	
	F4	5,751	HL20055-TAR-S4-F	cgtcggaactgttatcaagaacg	
			HL19196-S4-R2	aatcaattcgcggtcaagtcc	
	F5	5,206	HL20031-S4-F3	gaagcgaacggatggacgac	
			HL20058-TAR-S4-R	cggtaatcgcagattgaacaataagc	
	F6	5,138	HL20057-TAR-S4-F	cgcaaacgctgtataacgaagg	
			HL19198-S4-R3	agtatcccgaagttctttccgc	
	F7	5,600	HL20032-S4-F4	tttatcgggcgatgtttcggc	
			HL20054-TAR-S4-R	gctttcgacgctgttaacgc	
	F8	5,457	HL20053-TAR-S4-F	aagcgcatcgtaccaaaagc	
			HL20101-S4-R8-313	GAGGTCGACGGTATCGATAAGCTTGATATC cttgtgttcgttggtatgggtat	
pYEP-II-S4-b1	F1	5,512	HL20033-S4-F1-F1	GGGGATCCTCTAGAGTCGACCTGCAGCTCG AGtcgaagggttgacgtactgtcg	The plasmid contains unit-length genome and 60bp circularly permuted sequence from phage S4. The first and last genome fragments for TAR cloning are amplified with primers that carry ‘‘arms’’ that have homology with vector, and the first genome fragment has 60 bp homologous sequence to the last genome fragment. The first nucleotide of first genome fragment was located between ORF45 and ORF46. The plasmid pYEP-II was digested by SphI and HindIII for TAR cloning vector.<br>Primer pairs:<br>HL20069-TAR-S4-F1-1 and
			HL20034-S4-F1-F2	GACTCACTATAGGGCGAATTCGAGCTCGGT ACCCGGGGATCCTCTAGAGTCGACCTG	
			HL20052-TAR-S4-R	acgatggaaagcgcgaaaaca	
	F2	4,837	HL20051-TAR-S4-F	gtcgattccgccgcagtatatg	
			HL19194-S4-R1	gcttattgtccgatagtgccatgat	
	F3	6,007	HL20030-S4-F2	ggcgataatgtgggcaacaacg	
			HL20056-TAR-S4-R	tgatacgaacgacgcttgataagtg	
	F4	5,751	HL20055-TAR-S4-F	cgtcggaactgttatcaagaacg	
			HL19196-S4-R2	aatcaattcgcggtcaagtcc	
	F5	5,206	HL20031-S4-F3	gaagcgaacggatggacgac	
			HL20058-TAR-S4-R	cggtaatcgcagattgaacaataagc	
	F6	5,138	HL20057-TAR-S4-F	cgcaaacgctgtataacgaagg	

			HL19198-S4-R3	agtatcccgaagttctttccgc	HL20052-TAR-S4-R
	F7	5,600	HL20032-S4-F4	tttatcgggcgatgtttcggc	HL20070-S4-TAR-F1-2 and HL20052-TAR-S4-R
			HL20054-TAR-S4-R	gctttcgacgctgttaacgc	
			HL20053-TAR-S4-F	aagcgcatcgtaccaaaagc	HL20051-TAR-S4-F and HL19194-S4-R1
			HL20035-S4-F4-R1	AGCTATTTAGGTGACACTATAGAATACTCCTCGAGcttgtgttcgttggtatgggtata	HL20030-S4-F2 and HL20056-TAR-S4-R<br>HL20055-TAR-S4-F and HL19196-S4-R2<br>HL20031-S4-F3 and HL20058-TAR-S4-R
	F8	5,493			HL20057-TAR-S4-F and HL19198-S4-R3<br>HL20032-S4-F4 and HL20054-TAR-S4-R
			HL20036-S4-F4-R2	ACAGGAAACAGCTATGACCATGATTACGCCAAGCTATTTAGGTGACACTATAGAATACT	HL20053-TAR-S4-F and HL20035-S4-F4-R1<br>HL20053-TAR-S4-F and HL20036-S4-F4-R2
pRSII313-S4-a1-RFP	Vector	4,981	HL20102-pRS313-F-S4	gtactatatacccataccaacgaacacaagGATATCAAGCTTATCGATACCGTCG	The plasmid contains unit-length genome and 60bp circularly permuted sequence from phage S4 and the RFP label which located at the downstream of ORF11. The first and last genome fragments for TAR cloning are amplified with primers that carry ‘‘arms’’ that have homology with vector, and the first genome fragment has 60bp homologous sequence to the last genome fragment. The first nucleotide of first genome fragment was located between ORF45 and ORF46. The plasmid pRSII313 was linearized with primer pairs HL20102-pRS313-F-S4 and HL20132-pRS313-S4-R for TAR cloning vector.
			HL20132-pRS313-S4-R	gacggcttcgacagtacgtcaacccttcgaCACCGCGGTGGAGCTCCAATTC	
	F1	5,476	HL20131-S4-F1-313	TATAGGGCGAATTGGAGCTCCACCGCGGTGtcgaagggttgacgtactgtcg	
			HL20052-TAR-S4-R	acgatggaaagcgcgaaaaca	
	F2	4,837	HL20051-TAR-S4-F	gtcgattccgccgcagtatatg	
			HL19194-S4-R1	gcttattgtccgatagtgccatgat	
	F3-1	2,896	HL20030-S4-F2	ggcgataatgtgggcaacaacg	
			HL20104-S4-F3-RFP-R	acattatacgagccgatgattaattgtcaattagtgaacttcgacgacaacgc	
	F3-2	843	HL20105-RFP-S4-F	ttggcgggcgttgtcgtcgaagttcactaattgacaattaatcatcggctcgt	
			HL20106-RFP-S4-R	ggggccgaagccccgtccgtctgccgtccgttatttatacagttcgtccataccgc	Primer pairs:
	F3-3	3,137	HL20107-S4-F4-RFP-F	accggcggtatggacgaactgtataaataacggacggcagacggacggg	HL20102-pRS313-F-S4 and HL20132-pRS313-S4-RHL20131-S4-F1-313 and HL20052-TAR-S4-R
			HL20056-TAR-S4-R	tgatacgaacgacgcttgataagtg	
	F4	5,751	HL20055-TAR-S4-F	cgtcggaactgttatcaagaacg	HL20051-TAR-S4-F and HL19194-S4-R1

			HL19196-S4-R2	aatcaattcgcggtcaagtcc	HL20030-S4-F2 and HL20104-S4-F3-RFP-R
	F5	5,206	HL20031-S4-F3	gaagcgaacggatggacgac	HL20105-RFP-S4-F and
			HL20058-TAR-S4-R	cggtaatcgcagattgaacaataagc	HL20106-RFP-S4-R
	F6	5,138	HL20057-TAR-S4-F	cgcaaacgctgtataacgaagg	HL20107-S4-F4-RFP-F and
			HL19198-S4-R3	agtatcccgaagttctttccgc	HL20056-TAR-S4-R
	F7	5,600	HL20032-S4-F4	tttatcgggcgatgtttcggc	HL20055-TAR-S4-F and HL19196-S4-R2
			HL20054-TAR-S4-R	gctttcgacgctgttaacgc	HL20031-S4-F3 and HL20058-TAR-S4-R
	F8	5,457	HL20053-TAR-S4-F	aagcgcatcgtaccaaaagc	HL20057-TAR-S4-F and HL19198-S4-R3<br>HL20032-S4-F4 and HL20054-TAR-S4-R
			HL20101-S4-R8-313	GAGGTCGACGGTATCGATAAGCTTGATATC cttgtgttcgttggtatgggtat	HL20053-TAR-S4-F and HL20101-S4-R8-313
pYEP-II-S4-b1-RFP	F1	5,512	HL20033-S4-F1-F1	GGGGATCCTCTAGAGTCGACCTGCAGCTCGAGtcgaagggttgacgtactgtcg	The plasmid contains unit-length genome and 60bp circularly permuted sequence from phage S4 and the RFP label which located at the downstream of ORF11. The first and last genome fragments for TAR cloning are amplified with primers that carry ''arms'' that have homology with vector, and the first genome fragment has 60 bp homologous sequence to the last genome fragment. The first nucleotide of first genome fragments was located between ORF45 and ORF46<br>The plasmid pYEP-II was digested by SphI and HindIII for TAR cloning vector<br>Primer pairs:<br>HL20033-S4-F1-F1 and HL20052-TAR-S4-R<br>HL20034-S4-F1-F2 and HL20052-TAR-S4-R<br>HL20051-TAR-S4-F and HL19194-S4-R1<br>HL20030-S4-F2 and HL20104-S4-F3-RFP-R<br>HL20105-RFP-S4-F and
			HL20034-S4-F1-F2	GACTCACTATAGGGCGAATTCGAGCTCGGTACCCGGGGATCCTCTAGAGTCGACCTG	
			HL20052-TAR-S4-R	acgatggaaagcgcgaaaaca	
	F2	4,837	HL20051-TAR-S4-F	gtcgattccgccgcagtatatg	
			HL19194-S4-R1	gcttattgtccgatagtgccatgat	
	F3-1	2,896	HL20030-S4-F2	ggcgataatgtgggcaacaacg	
			HL20104-S4-F3-RFP-R	acattatacgagccgatgattaattgtcaattagtgaacttcgacgacaacgc	
	F3-2	843	HL20105-RFP-S4-F	ttggcgggcgttgtcgtcgaagttcactaattgacaattaatcatcggctcgt	
			HL20106-RFP-S4-R	ggggccgaagccccgtccgtctgccgtccgttatttatacagttcgtccataccgc	
	F3-3	3,137	HL20107-S4-F4-RFP-F	accggcggtatggacgaactgtataaataacggacggcagacggacggg	
			HL20056-TAR-S4-R	tgatacgaacgacgcttgataagtg	
	F4	5,751	HL20055-TAR-S4-F	cgtcggaactgttatcaagaacg	
			HL19196-S4-R2	aatcaattcgcggtcaagtcc	
	F5	5,206	HL20031-S4-F3	gaagcgaacggatggacgac	
			HL20058-TAR-S4-R	cggtaatcgcagattgaacaataagc	

	F6	5,138	HL20057-TAR-S4-F	cgcaaacgctgtataacgaagg	HL20106-RFP-S4-R
			HL19198-S4-R3	agtatcccgaagttctttccgc	HL20107-S4-F4-RFP-F and
	F7	5,600	HL20032-S4-F4	tttatcgggcgatgtttcggc	HL20056-TAR-S4-R
			HL20054-TAR-S4-R	gctttcgacgctgttaacgc	HL20055-TAR-S4-F and HL19196-S4-R2
	F8	5,493	HL20053-TAR-S4-F	aagcgcatcgtaccaaaagc	HL20031-S4-F3 and HL20058-TAR-S4-R
			HL20035-S4-F4-R1	AGCTATTTAGGTGACACTATAGAATACTCC TCGAGcttgtgttcgttggtatgggtata	HL20057-TAR-S4-F and HL19198-S4-R3<br>HL20032-S4-F4 and HL20054-TAR-S4-R
			HL20036-S4-F4-R2	ACAGGAAACAGCTATGACCATGATTACGCC AAGCTATTTAGGTGACACTATAGAATACT	HL20053-TAR-S4-F and HL20035-S4-F4-R1<br>HL20053-TAR-S4-F and HL20036-S4-F4-R2
pRSII313 -S4-a2	Vector	4,981	HL20184-pRS313-F	acgacggcccttcgttactgtcaagccccctGATATCAAGCTT ATCGATACCGTCGAC	The plasmid contains unit-length genome and 60bp circularly permuted sequence from phage S4. The first and last genome fragments for TAR cloning are amplified with primers that carry ''arms'' that have homology with vector, and the first genome fragment has 60 bp homologous sequence to the last genome fragment. The first nucleotide of first genome fragment was located between ORF35 and ORF36
			HL20185-pRS313-R	atagttcgtacataccggcgcaattgtgcgCACCGCGGTGGA GCTCCAATT	
	F1	5,657	HL20186-F1	GGATCCTCTAGAGTCGACCTGCAGCTCGAG cgcacaattgcgccggtatgta	
			HL20181-R1	cttgtgttcgttggtatgggtat	
	F2	5,446	HL20182-F2	tcgaagggttgacgtactgtcg	
			HL20052-TAR-S4-R	acgatggaaagcgcgaaaaca	
	F3	4,837	HL20051-TAR-S4-F	gtcgattccgccgcagtatatg	
			HL19194-S4-R1	gcttattgtccgatagtgccatgat	The plasmid pRSII313 was linearized with primer pairs HL20184-pRS313-F and HL20185-pRS313-R for TAR cloning vector
	F4	6,007	HL20030-S4-F2	ggcgataatgtgggcaacaacg	
			HL20056-TAR-S4-R	tgatacgaacgacgcttgataagtg	
	F5	5,751	HL20055-TAR-S4-F	cgtcggaactgttatcaagaacg	Primer pairs:<br>HL20184-pRS313-F and
			HL19196-S4-R2	aatcaattcgcggtcaagtcc	HL20185-pRS313-R
	F6	5,206	HL20031-S4-F3	gaagcgaacggatggacgac	HL20180-F1 and HL20181-R1
			HL20058-TAR-S4-R	cggtaatcgcagattgaacaataagc	HL20182-F2 and HL20052-TAR-S4-R
	F7	5,138	HL20057-TAR-S4-F	cgcaaacgctgtataacgaagg	HL20051-TAR-S4-F and HL19194-S4-R1
			HL19198-S4-R3	agtatcccgaagttctttccgc	HL20030-S4-F2 and HL20056-TAR-S4-R
	F8	5,430	HL20032-S4-F4	tttatcgggcgatgtttcggc	HL20055-TAR-S4-F and HL19196-S4-R2
			HL20183-R8	GAGGTCGACGGTATCGATAAGCTTGATATC	HL20031-S4-F3 and HL20058-TAR-S4-R

				aggggcttgacagtaacgaagg	HL20057-TAR-S4-F and HL19198-S4-R3<br>HL20032-S4-F4 and HL20183-R8
pYEP-II-S4-b2	Vector	12,023	HL20188-pYEP-F	acgacggcccttcgttactgtcaagcccctCTCGAGGAGTATTCTATAGTGTCACCT	The plasmid contains unit-length genome and 60bp circularly permuted sequence from phage S4. The first and last genome fragments for TAR cloning are amplified with primers that carry ‘‘arms’’ that have homology with vector, and the first genome fragment has 60 bp homologous sequence to the last genome fragment. The first nucleotide of first genome fragment was located between ORF35 and ORF36. The plasmid pYEP-Ⅱ was linearized with primer pairs HL20188-pYEP-F and HL20189-pYEP-R for TAR cloning vector.<br>Primer pairs:<br>HL20188-pYEP-F and HL20189-pYEP-R<br>HL20186-F1 and HL20181-R1<br>HL20182-F2 and HL20052-TAR-S4-R<br>HL20051-TAR-S4-F and HL19194-S4-R1<br>HL20030-S4-F2 and HL20056-TAR-S4-R<br>HL20055-TAR-S4-F and HL19196-S4-R2<br>HL20031-S4-F3 and HL20058-TAR-S4-R<br>HL20057-TAR-S4-F and HL19198-S4-R3<br>HL20032-S4-F4 and HL20187-R8
			HL20189-pYEP-R	atagttcgtacataccggcgcaattgtgcgCTCGAGCTGCAGGTCGACTCTA	
	F1	5,657	HL20186-F1	GGATCCTCTAGAGTCGACCTGCAGCTCGAGcgcacaattgcgccggtatgta	
			HL20181-R1	cttgtgttcgttggtatgggtat	
	F2	5,446	HL20182-F2	tcgaagggttgacgtactgtcg	
			HL20052-TAR-S4-R	acgatggaaagcgcgaaaaca	
	F3	4,837	HL20051-TAR-S4-F	gtcgattccgccgcagtatatg	
			HL19194-S4-R1	gcttattgtccgatagtgccatgat	
	F4	6,007	HL20030-S4-F2	ggcgataatgtgggcaacaacg	
			HL20056-TAR-S4-R	tgatacgaacgacgcttgataagtg	
	F5	5,751	HL20055-TAR-S4-F	cgtcggaactgttatcaagaacg	
			HL19196-S4-R2	aatcaattcgcggtcaagtcc	
	F6	5,206	HL20031-S4-F3	gaagcgaacggatggacgac	
			HL20058-TAR-S4-R	cggtaatcgcagattgaacaataagc	
	F7	5,138	HL20057-TAR-S4-F	cgcaaacgctgtataacgaagg	
			HL19198-S4-R3	agtatcccgaagttctttccgc	
	F8	5,430	HL20032-S4-F4	tttatcgggcgatgtttcggc	
			HL20187-R8	TTTAGGTGACACTATAGAATACTCCTCGAGaggggcttgacagtaacgaagg	
pRSII313-S4-a2-RFP	Vector	4,981	HL20184-pRS313-F	acgacggcccttcgttactgtcaagcccctGATATCAAGCTTATCGATACCGTCGAC	The plasmid contains unit-length genome and 60bp circularly permuted sequence from phage S4 and RFP label which located at the downstream of ORF11. The first and last genome fragments for TAR cloning are amplified with primers that carry ‘‘arms’’ that have homology with vector, and the first genome fragment has 60 bp homologous
			HL20185-pRS313-R	atagttcgtacataccggcgcaattgtgcgCACCGCGGTGGAGCTCCAATT	
	F1	5,657	HL20180-F1	TATAGGGCGAATTGGAGCTCCACCGCGGTGcgcacaattgcgccggtatgta	
			HL20181-R1	cttgtgttcgttggtatgggtat	
	F2	5,446	HL20182-F2	tcgaagggttgacgtactgtcg	

			HL20052-TAR-S4-R	acgatggaaagcgcgaaaaca	sequence to the last genome fragment. The first nucleotide of first genome fragment was located between ORF35 and ORF36. The plasmid pRSII313 was linearized with primer pairs HL20184-pRS313-F and HL20185-pRS313-R for TAR cloning vector Primer pairs:<br>HL20184-pRS313-F and HL20185-pRS313-R<br>HL20180-F1 and HL20181-R1<br>HL20182-F2 and HL20052-TAR-S4-R<br>HL20051-TAR-S4-F and HL19194-S4-R1<br>HL20030-S4-F2 and HL20104-S4-F3-RFP-R<br>HL20105-RFP-S4-F and HL20106-RFP-S4-R<br>HL20107-S4-F4-RFP-F and HL20056-TAR-S4-R<br>HL20055-TAR-S4-F and HL19196-S4-R2<br>HL20031-S4-F3 and HL20058-TAR-S4-R<br>HL20057-TAR-S4-F and HL19198-S4-R3<br>HL20032-S4-F4 and HL20183-R8
	F3	4,837	HL20051-TAR-S4-F	gtcgattccgccgcagtatatg	
			HL19194-S4-R1	gcttattgtccgatagtgccatgat	
	F4-1	2,896	HL20030-S4-F2	ggcgataatgtgggcaacaacg	
			HL20104-S4-F3-RFP-R	acattatacgagccgatgattaattgtcaattagtgaacttcgacgacaacgc	
	F4-2	843	HL20105-RFP-S4-F	ttggcgggcgttgtcgtcgaagttcactaattgacaattaatcatcggctcgt	
			HL20106-RFP-S4-R	ggggccgaagccccgtccgtctgccgtccgttatttatacagttcgtccataccgc	
	F4-3	3,171	HL20107-S4-F4-RFP-F	accggcggtatggacgaactgtataaataacggacggcagacggacggg	
			HL20056-TAR-S4-R	tgatacgaacgacgcttgataagtg	
	F5	5,751	HL20055-TAR-S4-F	cgtcggaactgttatcaagaacg	
			HL19196-S4-R2	aatcaattcgcggtcaagtcc	
	F6	5,206	HL20031-S4-F3	gaagcgaacggatggacgac	
			HL20058-TAR-S4-R	cggtaatcgcagattgaacaataagc	
	F7	5,138	HL20057-TAR-S4-F	cgcaaacgctgtataacgaagg	
			HL19198-S4-R3	agtatcccgaagttctttccgc	
	F8	5,430	HL20032-S4-F4	tttatcgggcgatgtttcggc	
			HL20183-R8	GAGGTCGACGGTATCGATAAGCTTGATATCaggggcttgacagtaacgaagg	
pYEP--S4-b2-RFP	Vector	12,023	HL20188-pYEP-F	acgacggcccttcgttactgtcaagcccctCTCGAGGAGTATTCTATAGTGTCACCT	The plasmid contains unit-length genome and 60bp circularly permuted sequence from phage S4 and RFP label which located at the downstream of ORF11. The first and last genome fragments for TAR cloning are amplified with primers that carry ‘‘arms’’ that have homology with vector, and the first genome fragment has 60 bp homologous sequence to the last genome fragment. The first nucleotide of first genome fragment was
			HL20189-pYEP-R	atagttcgtacataccggcgcaattgtgcgCTCGAGCTGCAGGTCGACTCTA	
	F1	5,657	HL20186-F1	GGATCCTCTAGAGTCGACCTGCAGCTCGAGcgcacaattgcgccggtatgta	
			HL20181-R1	cttgtgttcgttggtatgggtat	
	F2	5,446	HL20182-F2	tcgaagggttgacgtactgtcg	
			HL20052-TAR-S4-R	acgatggaaagcgcgaaaaca	
	F3	4,837	HL20051-TAR-S4-F	gtcgattccgccgcagtatatg	

			HL19194-S4-R1	gcttattgtccgatagtgccatgat	located between ORF35 and ORF36. The plasmid pYEP-Ⅱ was linearized with primer pairs HL20188-pYEP-F and HL20189-pYEP-R for TAR cloning vector. Primer pairs: HL20188-pYEP-F and HL20189-pYEP-R HL20186-F1 and HL20181-R1 HL20182-F2 and HL20052-TAR-S4-R HL20051-TAR-S4-F and HL19194-S4-R1 HL20030-S4-F2 and HL20104-S4-F3-RFP-R HL20105-RFP-S4-F and HL20106-RFP-S4-R HL20107-S4-F4-RFP-F and HL20056-TAR-S4-R HL20055-TAR-S4-F and HL19196-S4-R2 HL20031-S4-F3 and HL20058-TAR-S4-R HL20057-TAR-S4-F and HL19198-S4-R3 HL20032-S4-F4 and HL20187-R8
	F4-1	2,896	HL20030-S4-F2	ggcgataatgtgggcaacaacg	
			HL20104-S4-F3-RFP-R	acattatacgagccgatgattaattgtcaattagtgaacttcgacgacaacgc	
	F4-2	843	HL20105-RFP-S4-F	ttggcgggcgttgtcgtcgaagttcactaattgacaattaatcatcggctcgt	
			HL20106-RFP-S4-R	ggggccgaagccccgtccgtctgccgtccgttatttatacagttcgtccataccgc	
	F4-3	3,171	HL20107-S4-F4-RFP-F	accggcggtatggacgaactgtataaataacggacggcagacggacggg	
			HL20056-TAR-S4-R	tgatacgaacgacgcttgataagtg	
	F5	5,751	HL20055-TAR-S4-F	cgtcggaactgttatcaagaacg	
			HL19196-S4-R2	aatcaattcgcggtcaagtcc	
	F6	5,206	HL20031-S4-F3	gaagcgaacggatggacgac	
			HL20058-TAR-S4-R	cggtaatcgcagattgaacaataagc	
	F7	5,138	HL20057-TAR-S4-F	cgcaaacgctgtataacgaagg	
			HL19198-S4-R3	agtatcccgaagttctttccgc	
	F8	5,430	HL20032-S4-F4	tttatcgggcgatgtttcggc	
			HL20187-R8	TTTAGGTGACACTATAGAATACTCCTCGAGaggggcttgacagtaacgaagg	
pRSII313-S4-a3	Vector	4,981	HL20192-pRS313-F	cggccctttcttcattcaatcggagtaaatGATATCAAGCTTATCGATACCGTCGAC	The plasmid contains unit-length genome and 60bp circularly permuted sequence from phage S4. The first and last genome fragments for TAR cloning are amplified with primers that carry ‘‘arms’’ that have homology with vector, and the first genome fragment has 60 bp homologous sequence to the last genome fragment. The first nucleotide of first genome fragment was located between ORF17 and ORF18 The plasmid pRSII313 was linearized with primer pairs HL20192-pRS313-F and
			HL20193-pRS313-R	gtattacccggccctttgagtttaccgcaaCACCGCGGTGGAGCTCCAATT	
	F1	5,054	HL20190-F1	TATAGGGCGAATTGGAGCTCCACCGCGGTGttgcggtaaactcaaagggccg	
			HL19196-S4-R2	aatcaattcgcggtcaagtcc	
	F2	5,206	HL20031-S4-F3	gaagcgaacggatggacgac	
			HL20058-TAR-S4-R	cggtaatcgcagattgaacaataagc	
	F3	5,138	HL20057-TAR-S4-F	cgcaaacgctgtataacgaagg	
			HL19198-S4-R3	agtatcccgaagttctttccgc	
	F4	5,600	HL20032-S4-F4	tttatcgggcgatgtttcggc	

			HL20054-TAR-S4-R	gctttcgacgctgttaacgc	HL20193-pRS313-R for TAR cloning vector.<br>Primer pairs:<br>HL20192-pRS313-F and<br>HL20193-pRS313-R<br>HL20190-F1 and HL19196-S4-R2<br>HL20031-S4-F3 and HL20058-TAR-S4-R<br>HL20057-TAR-S4-F and HL19198-S4-R3<br>HL20032-S4-F4 and HL20054-TAR-S4-R<br>HL20053-TAR-S4-F and HL20181-R1<br>HL20182-F2 and HL20052-TAR-S4-R<br>HL20051-TAR-S4-F and HL19194-S4-R1<br>HL20030-S4-F2 and HL20191-R8
	F5	5,247	HL20053-TAR-S4-F	aagcgcatcgtaccaaaagc	
			HL20181-R1	cttgtgttcgttggtatgggtat	
	F6	5,446	HL20182-F2	tcgaagggttgacgtactgtcg	
			HL20052-TAR-S4-R	acgatggaaagcgcgaaaaca	
	F7	4,837	HL20051-TAR-S4-F	gtcgattccgccgcagtatatg	
			HL19194-S4-R1	gcttattgtccgatagtgccatgat	
	F8	6,664	HL20030-S4-F2	ggcgataatgtgggcaacaacg	
			HL20191-R8	GAGGTCGACGGTATCGATAAGCTTGATATC atttactccgattgaatgaagaaagggc	
pYEP-II-S4-b3	Vector	12,023	HL20196-pYEP-F	cggccctttcttcattcaatcggagtaaatCTCGAGGAGTATT CTATAGTGTCACCT	The plasmid contains unit-length genome and 60bp circularly permuted sequence from phage S4. The first and last genome fragments for TAR cloning are amplified with primers that carry ‘‘arms’’ that have homology with vector, and the first genome fragment has 60 bp homologous sequence to the last genome fragment. The first nucleotide of first genome fragment was located between ORF17 and ORF18<br>The plasmid pYEP-Ⅱ was linearized with primer pairs HL20188-pYEP-F and HL20197-pYEP-R for TAR cloning vector.<br>Primer pairs:<br>HL20188-pYEP-F and HL20197-pYEP-R<br>HL20194-F1 and HL19196-S4-R2<br>HL20031-S4-F3 and HL20058-TAR-S4-R<br>HL20057-TAR-S4-F and HL19198-S4-R3<br>HL20032-S4-F4 and HL20054-TAR-S4-R<br>HL20053-TAR-S4-F and HL20181-R1<br>HL20182-F2 and HL20052-TAR-S4-R
			HL20197-pYEP-R	gtattacccggccctttgagtttaccgcaaCTCGAGCTGCAG GTCGACTCTA	
	F1	5,054	HL20194-F1	GGATCCTCTAGAGTCGACCTGCAGCTCGAGt tgcggtaaactcaaagggccg	
			HL19196-S4-R2	aatcaattcgcggtcaagtcc	
	F2	5,206	HL20031-S4-F3	gaagcgaacggatggacgac	
			HL20058-TAR-S4-R	cggtaatcgcagattgaacaataagc	
	F3	5,138	HL20057-TAR-S4-F	cgcaaacgctgtataacgaagg	
			HL19198-S4-R3	agtatcccgaagttctttccgc	
	F4	5,600	HL20032-S4-F4	tttatcgggcgatgtttcggc	
			HL20054-TAR-S4-R	gctttcgacgctgttaacgc	
	F5	5,247	HL20053-TAR-S4-F	aagcgcatcgtaccaaaagc	
			HL20181-R1	cttgtgttcgttggtatgggtat	
	F6	5,446	HL20182-F2	tcgaagggttgacgtactgtcg	
			HL20052-TAR-S4-R	acgatggaaagcgcgaaaaca	
	F7	4,837	HL20051-TAR-S4-F	gtcgattccgccgcagtatatg	
			HL19194-S4-R1	gcttattgtccgatagtgccatgat	
	F8	6,664	HL20030-S4-F2	ggcgataatgtgggcaacaacg	

			HL20195-R8	TTTAGGTGACACTATAGAATACTCCTCGAGatttactccgattgaatgaagaaagggc	HL20051-TAR-S4-F and HL19194-S4-R1<br>HL20030-S4-F2 and HL20195-R8
pRSII313-S4-a3-RFP	Vector	4,981	HL20192-pRS313-F	cggccctttcttcattcaatcggagtaaatGATATCAAGCTTATCGATACCGTCGAC	The plasmid contains unit-length genome and 60bp circularly permuted sequence from phage S4 and RFP label which located at the downstream of ORF11. The first and last genome fragments for TAR cloning are amplified with primers that carry ‘‘arms’’ that have homology with vector, and the first genome fragment has 60 bp homologous sequence to the last genome fragment. The first nucleotide of first genome fragment was located between ORF17 and ORF18. The plasmid pRSII313 was linearized with primer pairs HL20192-pRS313-F and HL20193-pRS313-R for TAR cloning vector.<br>Primer pairs:<br>HL20192-pRS313-F and HL20193-pRS313-R<br>HL20190-F1 and HL19196-S4-R2<br>HL20031-S4-F3 and HL20058-TAR-S4-R<br>HL20057-TAR-S4-F and HL19198-S4-R3<br>HL20032-S4-F4 and HL20054-TAR-S4-R<br>HL20053-TAR-S4-F and HL20181-R1<br>HL20182-F2 and HL20052-TAR-S4-R<br>HL20051-TAR-S4-F and HL19194-S4-R1<br>HL20030-S4-F2 and HL20104-S4-F3-RFP-R<br>HL20105-RFP-S4-F and HL20106-RFP-S4-R<br>HL20107-S4-F4-RFP-F and HL20191-R8
			HL20193-pRS313-R	gtattacccggccctttgagtttaccgcaaCACCGCGGTGGAGCTCCAATT	
	F1	5,054	HL20190-F1	TATAGGGCGAATTGGAGCTCCACCGCGGTGttgcggtaaactcaaagggccg	
			HL19196-S4-R2	aatcaattcgcggtcaagtcc	
	F2	5,206	HL20031-S4-F3	gaagcgaacggatggacgac	
			HL20058-TAR-S4-R	cggtaatcgcagattgaacaataagc	
	F3	5,138	HL20057-TAR-S4-F	cgcaaacgctgtataacgaagg	
			HL19198-S4-R3	agtatcccgaagttctttccgc	
	F4	5,600	HL20032-S4-F4	tttatcgggcgatgtttcggc	
			HL20054-TAR-S4-R	gctttcgacgctgttaacgc	
	F5	5,247	HL20053-TAR-S4-F	aagcgcatcgtaccaaaagc	
			HL20181-R1	cttgtgttcgttggtatgggtat	
	F6	5,446	HL20182-F2	tcgaagggttgacgtactgtcg	
			HL20052-TAR-S4-R	acgatggaaagcgcgaaaaca	
	F7	4,837	HL20051-TAR-S4-F	gtcgattccgccgcagtatatg	
			HL19194-S4-R1	gcttattgtccgatagtgccatgat	
	F8-1	2,896	HL20030-S4-F2	ggcgataatgtgggcaacaacg	
			HL20104-S4-F3-RFP-R	acattatacgagccgatgattaattgtcaattagtgaacttcgacgacaacgc	
	F8-2	843	HL20105-RFP-S4-F	ttggcgggcgttgtcgtcgaagttcactaattgacaattaatcatcggctcgt	
			HL20106-RFP-S4-R	ggggccgaagccccgtccgtctgccgtccgttatttatacagttcgtccataccgc	
	F8-3	3,928	HL20107-S4-F4-RFP-F	accggcggtatggacgaactgtataaataacggacggcagacggacggg	
			HL20191-R8	GAGGTCGACGGTATCGATAAGCTTGATATCatttactccgattgaatgaagaaagggc	

pYEP-II-S4-b3-RFP	Vector	12,023	HL20196-pYEP-F	cggccctttcttcattcaatcggagtaaatCTCGAGGAGTATTCTATAGTGTCACCT	The plasmid contains unit-length genome and 60bp circularly permuted sequence from phage S4 and RFP label which located at the downstream of ORF11. The first and last genome fragments for TAR cloning are amplified with primers that carry ''arms'' that have homology with vector, and the first genome fragment has 60 bp homologous sequence to the last genome fragment. The first nucleotide of first genome fragment was located between ORF17 and ORF18. The plasmid pYEP-Ⅱ was linearized with primer pairs HL20188-pYEP-F and HL20197-pYEP-R for TAR cloning vector<br>Primer pairs:<br>HL20188-pYEP-F and HL20197-pYEP-R<br>HL20194-F1 and HL19196-S4-R2<br>HL20031-S4-F3 and HL20058-TAR-S4-R<br>HL20057-TAR-S4-F and HL19198-S4-R3<br>HL20032-S4-F4 and HL20054-TAR-S4-R<br>HL20053-TAR-S4-F and HL20181-R1<br>HL20182-F2 and HL20052-TAR-S4-R<br>HL20051-TAR-S4-F and HL19194-S4-R1<br>HL20030-S4-F2 and HL20104-S4-F3-RFP-R<br>HL20105-RFP-S4-F and HL20106-RFP-S4-R<br>HL20107-S4-F4-RFP-F and HL20195-R8
			HL20197-pYEP-R	gtattacccggccctttgagtttaccgcaaCTCGAGCTGCAGGTCGACTCTA	
	F1	5,054	HL20194-F1	GGATCCTCTAGAGTCGACCTGCAGCTCGAGttgcggtaaactcaaagggccg	
			HL19196-S4-R2	aatcaattcgcggtcaagtcc	
	F2	5,206	HL20031-S4-F3	gaagcgaacggatggacgac	
			HL20058-TAR-S4-R	cggtaatcgcagattgaacaataagc	
	F3	5,138	HL20057-TAR-S4-F	cgcaaacgctgtataacgaagg	
			HL19198-S4-R3	agtatcccgaagttctttccgc	
	F4	5,600	HL20032-S4-F4	tttatcgggcgatgtttcggc	
			HL20054-TAR-S4-R	gctttcgacgctgttaacgc	
	F5	5,247	HL20053-TAR-S4-F	aagcgcatcgtaccaaaagc	
			HL20181-R1	cttgtgttcgttggtatgggtat	
	F6	5,446	HL20182-F2	tcgaagggttgacgtactgtcg	
			HL20052-TAR-S4-R	acgatggaaagcgcgaaaaca	
	F7	4,837	HL20051-TAR-S4-F	gtcgattccgccgcagtatatg	
			HL19194-S4-R1	gcttattgtccgatagtgccatgat	
	F8-1	2,896	HL20030-S4-F2	ggcgataatgtgggcaacaacg	
			HL20104-S4-F3-RFP-R	acattatacgagccgatgattaattgtcaattagtgaacttcgacgacaacgc	
	F8-2	843	HL20105-RFP-S4-F	ttggcgggcgttgtcgtcgaagttcactaattgacaattaatcatcggctcgt	
			HL20106-RFP-S4-R	ggggccgaagccccgtccgtctgccgtccgttatttatacagttcgtccataccgc	
	F8-3	3,928	HL20107-S4-F4-RFP-F	accggcggtatggacgaactgtataaataacggacggcagacggacggg	
			HL20195-R8	TTTAGGTGACACTATAGAATACTCCTCGAGatttactccgattgaatgaagaaagggc	
pRSII313-S4-△	Vector	4,981	HL20184-pRS313-F	acgacggcccttcgttactgtcaagcccctGATATCAAGCTTATCGATACCGTCGAC	The plasmid contains unit-length genome (except ORF39-ORF43 region) and 60bp

gp39-43			HL20185-pRS313-R	atagttcgtacataccggcgcaattgtgcgCACCGCGGTGGAGCTCCAATT	circularly permuted sequence from phage S4. The first and last genome fragments for TAR cloning are amplified with primers that carry ‘‘arms’’ that have homology with vector, and the first genome fragment has 60 bp homologous sequence to the last genome fragment. The first nucleotide of first genome fragment was located between ORF35 and ORF36
	F1	3,456	HL20180-F1	TATAGGGCGAATTGGAGCTCCACCGCGGTGcgcacaattgcgccggtatgta	
			LC21125-43-R	atacgtgcccattatacgacccgtcggctaggcttcggttccttggtttgt	
	F2	977	LC21126-44-F	agcaacgaaacaaaccaaggaaccgaagcctagccgacgggtcgtataatggg	
			HL20181-R1	cttgtgttcgttggtatgggtat	
	F3	5,446	HL20182-F2	tcgaagggttgacgtactgtcg	The plasmid pRSII313 was linearized with primer pairs HL20184-pRS313-F and HL20185-pRS313-R for TAR cloning vector.
			HL20052-TAR-S4-R	acgatggaaagcgcgaaaaca	
	F4	4,837	HL20051-TAR-S4-F	gtcgattccgccgcagtatatg	
			HL19194-S4-R1	gcttattgtccgatagtgccatgat	Primer pairs:
	F5	6,007	HL20030-S4-F2	ggcgataatgtgggcaacaacg	HL20184-pRS313-F and HL20185-pRS313-R
			HL20056-TAR-S4-R	tgatacgaacgacgcttgataagtg	HL20180-F1 and LC21125-43-R
	F6	5,751	HL20055-TAR-S4-F	cgtcggaactgttatcaagaacg	LC21126-44-F and HL20181-R1
			HL19196-S4-R2	aatcaattcgcggtcaagtcc	HL20182-F2 and HL20052-TAR-S4-R
	F7	5,206	HL20031-S4-F3	gaagcgaacggatggacgac	HL20051-TAR-S4-F and HL19194-S4-R1
			HL20058-TAR-S4-R	cggtaatcgcagattgaacaataagc	HL20030-S4-F2 and HL20056-TAR-S4-R
	F8	5,138	HL20057-TAR-S4-F	cgcaaacgctgtataacgaagg	HL20055-TAR-S4-F and HL19196-S4-R2
			HL19198-S4-R3	agtatcccgaagttctttccgc	HL20031-S4-F3 and HL20058-TAR-S4-R
	F9	5,430	HL20032-S4-F4	tttatcgggcgatgtttcggc	HL20057-TAR-S4-F and HL19198-S4-R3
			HL20183-R8	GAGGTCGACGGTATCGATAAGCTTGATATCaggggcttgacagtaacgaagg	HL20032-S4-F4 and HL20183-R8
pRSII313-S4-△gp44-48	Vector	4,981	HL20184-pRS313-F	acgacggcccttcgttactgtcaagcccctGATATCAAGCTTATCGATACCGTCGAC	The plasmid contains unit-length genome (except ORF44-ORF48 region) and 60bp circularly permuted sequence from phage S4. The first and last genome fragments for TAR cloning are amplified with primers that carry ‘‘arms’’ that have homology with vector, and the first genome fragment has 60 bp homologous sequence to the last genome fragment. The first nucleotide of first genome
			HL20185-pRS313-R	atagttcgtacataccggcgcaattgtgcgCACCGCGGTGGAGCTCCAATT	
	F1	5,060	HL20180-F1	TATAGGGCGAATTGGAGCTCCACCGCGGTGcgcacaattgcgccggtatgta	
			LC21127-48-R	gccagacttggcaagggttgcgggcgtcattgcaagcccctttcgttagtgcg	
	F2	4,444	LC21128-49-F	tccggcacgcactaacgaaaggggcttgcaatgacgcccgcaacccctt	

				gccaa	fragment was located between ORF35 and
			HL20052-TAR-S4-R	acgatggaaagcgcgaaaaca	ORF36
	F3	4,837	HL20051-TAR-S4-F	gtcgattccgccgcagtatatg	The plasmid pRSII313 was linearized with
			HL19194-S4-R1	gcttattgtccgatagtgccatgat	primer pairs HL20184-pRS313-F and
	F4	6,007	HL20030-S4-F2	ggcgataatgtgggcaacaacg	HL20185-pRS313-R for TAR cloning vector.
			HL20056-TAR-S4-R	tgatacgaacgacgcttgataagtg	Primer pairs:
	F5	5,751	HL20055-TAR-S4-F	cgtcggaactgttatcaagaacg	HL20184-pRS313-F and
			HL19196-S4-R2	aatcaattcgcggtcaagtcc	HL20185-pRS313-R
	F6	5,206	HL20031-S4-F3	gaagcgaacggatggacgac	HL20180-F1 and LC21127-48-R
			HL20058-TAR-S4-R	cggtaatcgcagattgaacaataagc	LC21128-49-F and HL20052-TAR-S4-R
	F7	5,138	HL20057-TAR-S4-F	cgcaaacgctgtataacgaagg	HL20051-TAR-S4-F and HL19194-S4-R1
			HL19198-S4-R3	agtatcccgaagttctttccgc	HL20030-S4-F2 and HL20056-TAR-S4-R
	F8	5,430	HL20032-S4-F4	tttatcgggcgatgtttcggc	HL20055-TAR-S4-F and HL19196-S4-R2
			HL20183-R8	GAGGTCGACGGTATCGATAAGCTTGATATC aggggcttgacagtaacgaagg	HL20031-S4-F3 and HL20058-TAR-S4-R HL20057-TAR-S4-F and HL19198-S4-R3 HL20032-S4-F4 and HL20183-R8
	Vector	4,981	HL20184-pRS313-F	acgacggcccttcgttactgtcaagcccctGATATCAAGCTT ATCGATACCGTCGAC	The plasmid contains unit-length genome (except ORF50-ORF55 region) and 60bp circularly permuted sequence from phage S4
			HL20185-pRS313-R	AATTGGAGCTCCACCGCGGTGcgcacaattgcgcc ggtatgtacgaactat	
pRSII313 -S4-△ gp50-55	F1	5,657	HL20180-F1	TATAGGGCGAATTGGAGCTCCACCGCGGTG cgcacaattgcgccggtatgta	The first and last genome fragments for TAR cloning are amplified with primers that carry ‘‘arms’’ that have homology with vector, and the first genome fragment has 60 bp homologous sequence to the last genome fragment. The first nucleotide of first genome fragment was located between ORF35 and ORF36. The plasmid pRSII313 was linearized with primer pairs HL20184-pRS313-F and HL20185-pRS313-R for TAR cloning vector. Primer pairs: HL20184-pRS313-F and HL20185-pRS313-R
			HL20181-R1	cttgtgttcgttggtatgggtat	
	F2	1,598	HL20182-F2	tcgaagggttgacgtactgtcg	
			LC21129-55-R	ggcgtcgcgcggtgcgtagattatcgacactacgaaccccattcgatat agga	
	F3	2,404	LC21130-56-F	ccttcgttcctatatcgaatggggttcgtagtgtcgataatctacgcaccg cg	
			HL20052-TAR-S4-R	acgatggaaagcgcgaaaaca	
	F4	4,837	HL20051-TAR-S4-F	gtcgattccgccgcagtatatg	
			HL19194-S4-R1	gcttattgtccgatagtgccatgat	
	F5	6,007	HL20030-S4-F2	ggcgataatgtgggcaacaacg	
			HL20056-TAR-S4-R	tgatacgaacgacgcttgataagtg	

	F6	5,751	HL20055-TAR-S4-F	cgtcggaactgttatcaagaacg	HL20180-F1 and HL20181-R1
			HL19196-S4-R2	aatcaattcgcggtcaagtcc	HL20182-F2 and LC21129-55-R
	F7	5,206	HL20031-S4-F3	gaagcgaacggatggacgac	LC21130-56-F and HL20052-TAR-S4-R
			HL20058-TAR-S4-R	cggtaatcgcagattgaacaataagc	HL20051-TAR-S4-F and HL19194-S4-R1
	F8	5,138	HL20057-TAR-S4-F	cgcaaacgctgtataacgaagg	HL20030-S4-F2 and HL20056-TAR-S4-R
			HL19198-S4-R3	agtatcccgaagttctttccgc	HL20055-TAR-S4-F and HL19196-S4-R2
	F9	5,430	HL20032-S4-F4	tttatcgggcgatgtttcggc	HL20031-S4-F3 and HL20058-TAR-S4-R
			HL20183-R8	GAGGTCGACGGTATCGATAAGCTTGATATC aggggcttgacagtaacgaagg	HL20057-TAR-S4-F and HL19198-S4-R3<br>HL20032-S4-F4 and HL20183-R8
pRSII313-S4-△gp39-48	Vector	4,981	HL20184-pRS313-F	acgacggcccttcgttactgtcaagcccctGATATCAAGCTTATCGATACCGTCGAC	The plasmid contains unit-length genome (except ORF39-ORF43 region and ORF44-ORF48 region) and 60bp circularly permuted sequence from phage S4. The first and last genome fragments for TAR cloning are amplified with primers that carry ''arms'' that have homology with vector, and the first genome fragment has 60 bp homologous sequence to the last genome fragment. The first nucleotide of first genome fragment was located between ORF35 and ORF36. The plasmid pRSII313 was linearized with primer pairs HL20184-pRS313-F and HL20185-pRS313-R for TAR cloning vector.
			HL20185-pRS313-R	AATTGGAGCTCCACCGCGGTGcgcacaattgcgccggtatgtacgaactat	
	F1	3,456	HL20180-F1	TATAGGGCGAATTGGAGCTCCACCGCGGTGcgcacaattgcgccggtatgta	
			LC21125-43-R	atacgtgcccattatacgacccgtcggctaggcttcggttccttggtttgt	
	F2	385	LC21126-44-F	agcaacgaaacaaaccaaggaaccgaagcctagccgacgggtcgtataatggg	
			LC22078-48-R	tcggtgccagacttggcaagggttgcgggcgtcattgcaagcccctttcgttag	
	F3	4,439	LC22079-49-F	cacgcactaacgaaaggggcttgcaatgacgcccgcaacccctt	
			HL20052-TAR-S4-R	acgatggaaagcgcgaaaaca	
	F4	4,837	HL20051-TAR-S4-F	gtcgattccgccgcagtatatg	
			HL19194-S4-R1	gcttattgtccgatagtgccatgat	Primer pairs:
	F5	6,007	HL20030-S4-F2	ggcgataatgtgggcaacaacg	HL20184-pRS313-F and HL20185-pRS313-R
			HL20056-TAR-S4-R	tgatacgaacgacgcttgataagtg	
	F6	5,751	HL20055-TAR-S4-F	cgtcggaactgttatcaagaacg	HL20180-F1 and LC21125-43-R
			HL19196-S4-R2	aatcaattcgcggtcaagtcc	LC21126-44-F and LC22078-48-R
	F7	5,206	HL20031-S4-F3	gaagcgaacggatggacgac	LC22079-49-F and HL20052-TAR-S4-R
			HL20058-TAR-S4-R	cggtaatcgcagattgaacaataagc	HL20051-TAR-S4-F and HL19194-S4-R1
	F8	5,138	HL20057-TAR-S4-F	cgcaaacgctgtataacgaagg	HL20030-S4-F2 and HL20056-TAR-S4-R
			HL19198-S4-R3	agtatcccgaagttctttccgc	HL20055-TAR-S4-F and HL19196-S4-R2
	F9	5,430	HL20032-S4-F4	tttatcgggcgatgtttcggc	HL20031-S4-F3 and HL20058-TAR-S4-R

			HL20183-R8	GAGGTCGACGGTATCGATAAGCTTGATATC aggggcttgacagtaacgaagg	HL20057-TAR-S4-F and HL19198-S4-R3<br>HL20032-S4-F4 and HL20183-R8
pRSII313-S4-△gp39-55	Vector	4,981	HL20184-pRS313-F	acgacggcccttcgttactgtcaagcccctGATATCAAGCTTATCGATACCGTCGAC	The plasmid contains unit-length genome (except ORF39-ORF43 region, ORF44-ORF48 region and ORF50-ORF55 region) and 60bp circularly permuted sequence from phage S4. The first and last genome fragments for TAR cloning are amplified with primers that carry ''arms'' that have homology with vector, and the first genome fragment has 60 bp homologous sequence to the last genome fragment. The first nucleotide of first genome fragment was located between ORF35 and ORF36. The plasmid pRSII313 was linearized with primer pairs HL20184-pRS313-F and HL20185-pRS313-R for TAR cloning vector.<br>Primer pairs:<br>HL20184-pRS313-F and HL20185-pRS313-R<br>HL20180-F1 and LC21125-43-R<br>LC21126-44-F and LC22078-48-R<br>LC22079-49-F and HL20052-TAR-S4-R<br>HL20051-TAR-S4-F and HL19194-S4-R1<br>HL20030-S4-F2 and HL20056-TAR-S4-R<br>HL20055-TAR-S4-F and HL19196-S4-R2<br>HL20031-S4-F3 and HL20058-TAR-S4-R<br>HL20057-TAR-S4-F and HL19198-S4-R3<br>HL20032-S4-F4 and HL20183-R8
			HL20185-pRS313-R	AATTGGAGCTCCACCGCGGTGcgcacaattgcgccggtatgtacgaactat	
	F1	3,456	HL20180-F1	TATAGGGCGAATTGGAGCTCCACCGCGGTGcgcacaattgcgccggtatgta	
			LC21125-43-R	atacgtgcccattatacgacccgtcggctaggcttcggttccttggtttgt	
	F2	385	LC21126-44-F	agcaacgaaacaaaccaaggaaccgaagcctagccgacgggtcgtataatggg	
			LC22078-48-R	tcggtgccagacttggcaagggttgcgggcgtcattgcaagccccctttcgttag	
	F3	2,935	LC22079-49-F	cacgcactaacgaaagggggcttgcaatgacgcccgcaacccctt	
			HL20052-TAR-S4-R	acgatggaaagcgcgaaaaca	
	F4	4,837	HL20051-TAR-S4-F	gtcgattccgccgcagtatatg	
			HL19194-S4-R1	gcttattgtccgatagtgccatgat	
	F5	6,007	HL20030-S4-F2	ggcgataatgtgggcaacaacg	
			HL20056-TAR-S4-R	tgatacgaacgacgcttgataagtg	
	F6	5,751	HL20055-TAR-S4-F	cgtcggaactgttatcaagaacg	
			HL19196-S4-R2	aatcaattcgcggtcaagtcc	
	F7	5,206	HL20031-S4-F3	gaagcgaacggatggacgac	
			HL20058-TAR-S4-R	cggtaatcgcagattgaacaataagc	
	F8	5,138	HL20057-TAR-S4-F	cgcaaacgctgtataacgaagg	
			HL19198-S4-R3	agtatcccgaagttctttccgc	
	F9	5,430	HL20032-S4-F4	tttatcgggcgatgtttcggc	
			HL20183-R8	GAGGTCGACGGTATCGATAAGCTTGATATC aggggcttgacagtaacgaagg	

*Sequences from the vectors are indicated with capital letters.

Table S3 Primers uesd for verification

Yeast plasmid	Junction	Length of PCR products (bp)	Primer	5' → 3' sequence
pRSII313-S4-a0	V/F1	701	LH19005-pRSII313-F	GTGGCGAGAAAGGAAGGGAA
			HL20037-R	TCGACGGCCTGTTTAATATCGC
	F1/F2	795	HL20059-F2	GTTGGTGGATGCGCGTTATG
			HL20060-R1	TGCCCACAATAAAACCGACGT
	F2/F3	783	HL20038-F	TTGAATGGGCGTTGAAATGGGC
			HL20039-R	ATGCGTTCGTCGCTTCCTGTA
	F3/F4	866	HL20061-F4	GCGAACAACGGCCTTAAAACC
			HL20062-R3	GCTGCGACGGTCGTAAAGTAAG
	F4/F5	957	HL20040-F	CAATGGGCGTAAACGAAGTCG
			HL20041-R	TGGTAAATGGCCAACACCAAGG
	F5/F6	846	HL20063-F6	GCATTGATTGCATTTTGTGCCT
			HL20064-R5	GCGTCGAAACTTCCGTAGAC
	F6/F7	846	HL20042-F	ATTGTGGGCAATATGCCCGAA
			HL20043-R	GCAGTGTATCAACTGCGGCAC
	F7/F8	808	HL20065-F8	TGCGAAGCTGCGGTTAATCTT
			HL20066-R7	GCGTCGGATAAGTATTACGGCG
	F8/V	859	HL20044-F	CGAAGGTAACGAAGCATCGTTG
			LH19006-pRSII313-R	TACCGCCTTTGAGTGAGCTG
pYEP-II-S4-b0	V/F1	804	HL19052-V-F	ACAATGGAAGTCCGAGCTCATC

			HL20037-R	TCGACGGCCTGTTTAATATCGC
	F1/F2	795	HL20059-F2	GTTGGTGGATGCGCGTTATG
			HL20060-R1	TGCCCACAATAAAACCGACGT
	F2/F3	783	HL20038-F	TTGAATGGGCGTTGAAATGGGC
			HL20039-R	ATGCGTTCGTCGCTTCCTGTA
	F3/F4	866	HL20061-F4	GCGAACAACGGCCTTAAAACC
			HL20062-R3	GCTGCGACGGTCGTAAAGTAAG
	F4/F5	957	HL20040-F	CAATGGGCGTAAACGAAGTCG
			HL20041-R	TGGTAAATGGCCAACACCAAGG
	F5/F6	846	HL20063-F6	GCATTGATTGCATTTTGTGCCT
			HL20064-R5	GCGTCGAAACTTCCGTAGAC
	F6/F7	846	HL20042-F	ATTGTGGGCAATATGCCCGAA
			HL20043-R	GCAGTGTATCAACTGCGGCAC
	F7/F8	808	HL20065-F8	TGCGAAGCTGCGGTTAATCTT
			HL20066-R7	GCGTCGGATAAGTATTACGGCG
	F8/V	623	HL20044-F	CGAAGGTAACGAAGCATCGTTG
			HL19053-V-R	GCTCACTCATTAGGCACCCCA
	V/F1	761	LH19005-pRSII313-F	GTGGCGAGAAAGGAAGGGAA
			HL20037-R	TCGACGGCCTGTTTAATATCGC
pRSII313-S4-a1	F1/F2	795	HL20059-F2	GTTGGTGGATGCGCGTTATG
			HL20060-R1	TGCCCACAATAAAACCGACGT
	F2/F3	783	HL20038-F	TTGAATGGGCGTTGAAATGGGC

			HL20039-R	ATGCGTTCGTCGCTTCCTGTA
	F3/F4	866	HL20061-F4	GCGAACAACGGCCTTAAAACC
			HL20062-R3	GCTGCGACGGTCGTAAAGTAAG
	F4/F5	957	HL20040-F	CAATGGGCGTAAACGAAGTCG
			HL20041-R	TGGTAAATGGCCAACACCAAGG
	F5/F6	846	HL20063-F6	GCATTGATTGCATTTTGTGCCT
			HL20064-R5	GCGTCGAAACTTCCGTAGAC
	F6/F7	846	HL20042-F	ATTGTGGGCAATATGCCCGAA
			HL20043-R	GCAGTGTATCAACTGCGGCAC
	F7/F8	808	HL20065-F8	TGCGAAGCTGCGGTTAATCTT
			HL20066-R7	GCGTCGGATAAGTATTACGGCG
	F8/V	859	HL20044-F	CGAAGGTAACGAAGCATCGTTG
			LH19006-pRSII313-R	TACCGCCTTTGAGTGAGCTG
	V/F1	864	HL19052-V-F	ACAATGGAAGTCCGAGCTCATC
			HL20037-R	TCGACGGCCTGTTTAATATCGC
	F1/F2	795	HL20059-F2	GTTGGTGGATGCGCGTTATG
			HL20060-R1	TGCCCACAATAAAACCGACGT
pYEP-II-S4-b1	F2/F3	783	HL20038-F	TTGAATGGGCGTTGAAATGGGC
			HL20039-R	ATGCGTTCGTCGCTTCCTGTA
	F3/F4	866	HL20061-F4	GCGAACAACGGCCTTAAAACC
			HL20062-R3	GCTGCGACGGTCGTAAAGTAAG
	F4/F5	957	HL20040-F	CAATGGGCGTAAACGAAGTCG

			HL20041-R	TGGTAAATGGCCAACACCAAGG
	F5/F6	846	HL20063-F6	GCATTGATTGCATTTTGTGCCT
			HL20064-R5	GCGTCGAAACTTCCGTAGAC
	F6/F7	846	HL20042-F	ATTGTGGGCAATATGCCCGAA
			HL20043-R	GCAGTGTATCAACTGCGGCAC
	F7/F8	808	HL20065-F8	TGCGAAGCTGCGGTTAATCTT
			HL20066-R7	GCGTCGGATAAGTATTACGGCG
	F8/V	623	HL20044-F	CGAAGGTAACGAAGCATCGTTG
			HL19053-V-R	GCTCACTCATTAGGCACCCCA
	V/F1	761	LH19005-pRSII313-F	GTGGCGAGAAAGGAAGGGAA
			HL20037-R	TCGACGGCCTGTTTAATATCGC
	F1/F2	795	HL20059-F2	GTTGGTGGATGCGCGTTATG
			HL20060-R1	TGCCCACAATAAAACCGACGT
	F2/F3-1	783	HL20038-F	TTGAATGGGCGTTGAAATGGGC
			HL20039-R	ATGCGTTCGTCGCTTCCTGTA
pRSII313-S4-a1-RFP	F3-1/F3-3	1,372	HL20108-V-RFP-F	CGCCTGTTTACTTACGGCACC
			HL20109-V-RFP-R	CGCAGATTTCCAGCGAATGC
	F3-3/F4	866	HL20061-F4	GCGAACAACGGCCTTAAAACC
			HL20062-R3	GCTGCGACGGTCGTAAAGTAAG
	F4/F5	957	HL20040-F	CAATGGGCGTAAACGAAGTCG
			HL20041-R	TGGTAAATGGCCAACACCAAGG
	F5/F6	846	HL20063-F6	GCATTGATTGCATTTTGTGCCT

			HL20064-R5	GCGTCGAAACTTCCGTAGAC
	F6/F7	846	HL20042-F	ATTGTGGGCAATATGCCCGAA
			HL20043-R	GCAGTGTATCAACTGCGGCAC
	F7/F8	808	HL20065-F8	TGCGAAGCTGCGGTTAATCTT
			HL20066-R7	GCGTCGGATAAGTATTACGGCG
	F8/V	859	HL20044-F	CGAAGGTAACGAAGCATCGTTG
			LH19006-pRSII313-R	TACCGCCTTTGAGTGAGCTG
	V/F1	864	HL19052-V-F	ACAATGGAAGTCCGAGCTCATC
			HL20037-R	TCGACGGCCTGTTTAATATCGC
	F1/F2	795	HL20059-F2	GTTGGTGGATGCGCGTTATG
			HL20060-R1	TGCCCACAATAAAACCGACGT
	F2/F3-1	783	HL20038-F	TTGAATGGGCGTTGAAATGGGC
			HL20039-R	ATGCGTTCGTCGCTTCCTGTA
	F3-1/F3-3	1,372	HL20108-V-RFP-F	CGCCTGTTTACTTACGGCACC
pYEP-II-S4-b1-RFP			HL20109-V-RFP-R	CGCAGATTTCCAGCGAATGC
	F3-3/F4	866	HL20061-F4	GCGAACAACGGCCTTAAAACC
			HL20062-R3	GCTGCGACGGTCGTAAAGTAAG
	F4/F5	957	HL20040-F	CAATGGGCGTAAACGAAGTCG
			HL20041-R	TGGTAAATGGCCAACACCAAGG
	F5/F6	846	HL20063-F6	GCATTGATTGCATTTTGTGCCT
			HL20064-R5	GCGTCGAAACTTCCGTAGAC
	F6/F7	846	HL20042-F	ATTGTGGGCAATATGCCCGAA

			HL20043-R	GCAGTGTATCAACTGCGGCAC
	F7/F8	808	HL20065-F8	TGCGAAGCTGCGGTTAATCTT
			HL20066-R7	GCGTCGGATAAGTATTACGGCG
	F8/V	623	HL20044-F	CGAAGGTAACGAAGCATCGTTG
			HL19053-V-R	GCTCACTCATTAGGCACCCCA
	V/F1	884	LH19005-pRSII313-F	GTGGCGAGAAAGGAAGGGAA
			HL20066-R7	GCGTCGGATAAGTATTACGGCG
	F1/F2	826	HL20044-F	CGAAGGTAACGAAGCATCGTTG
			HL20037-R	TCGACGGCCTGTTTAATATCGC
	F2/F3	795	HL20059-F2	GTTGGTGGATGCGCGTTATG
			HL20060-R1	TGCCCACAATAAAACCGACGT
	F3/F4	783	HL20038-F	TTGAATGGGCGTTGAAATGGGC
			HL20039-R	ATGCGTTCGTCGCTTCCTGTA
pRSII313-S4-a2	F4/F5	866	HL20061-F4	GCGAACAACGGCCTTAAAACC
			HL20062-R3	GCTGCGACGGTCGTAAAGTAAG
	F5/F6	957	HL20040-F	CAATGGGCGTAAACGAAGTCG
			HL20041-R	TGGTAAATGGCCAACACCAAGG
	F6/F7	846	HL20063-F6	GCATTGATTGCATTTTGTGCCT
			HL20064-R5	GCGTCGAAACTTCCGTAGAC
	F7/F8	846	HL20042-F	ATTGTGGGCAATATGCCCGAA
			HL20043-R	GCAGTGTATCAACTGCGGCAC
	F8/V	710	HL20065-F8	TGCGAAGCTGCGGTTAATCTT

			LH19006-pRSII313-R	TACCGCCTTTGAGTGAGCTG
	V/F1	1,007	HL19052-V-F	ACAATGGAAGTCCGAGCTCATC
			HL20066-R7	GCGTCGGATAAGTATTACGGCG
	F1/F2	826	HL20044-F	CGAAGGTAACGAAGCATCGTTG
			HL20037-R	TCGACGGCCTGTTTAATATCGC
	F2/F3	795	HL20059-F2	GTTGGTGGATGCGCGTTATG
			HL20060-R1	TGCCCACAATAAAACCGACGT
	F3/F4	783	HL20038-F	TTGAATGGGCGTTGAAATGGGC
			HL20039-R	ATGCGTTCGTCGCTTCCTGTA
pYEP-II-S4-b2	F4/F5	866	HL20061-F4	GCGAACAACGGCCTTAAAACC
			HL20062-R3	GCTGCGACGGTCGTAAAGTAAG
	F5/F6	957	HL20040-F	CAATGGGCGTAAACGAAGTCG
			HL20041-R	TGGTAAATGGCCAACACCAAGG
	F6/F7	846	HL20063-F6	GCATTGATTGCATTTTGTGCCT
			HL20064-R5	GCGTCGAAACTTCCGTAGAC
	F7/F8	846	HL20042-F	ATTGTGGGCAATATGCCCGAA
			HL20043-R	GCAGTGTATCAACTGCGGCAC
	F8/V	462	HL20065-F8	TGCGAAGCTGCGGTTAATCTT
			HL19053-V-R	GCTCACTCATTAGGCACCCCA
	V/F1	884	LH19005-pRSII313-F	GTGGCGAGAAAGGAAGGGAA
pRSII313-S4-a2-RFP			HL20066-R7	GCGTCGGATAAGTATTACGGCG
	F1/F2	826	HL20044-F	CGAAGGTAACGAAGCATCGTTG

			HL20037-R	TCGACGGCCTGTTTAATATCGC
	F2/F3	795	HL20059-F2	GTTGGTGGATGCGCGTTATG
			HL20060-R1	TGCCCACAATAAAACCGACGT
	F3/F4-1	783	HL20038-F	TTGAATGGGCGTTGAAATGGGC
			HL20039-R	ATGCGTTCGTCGCTTCCTGTA
	F4-1/F4-3	1,372	HL20108-V-RFP-F	CGCCTGTTTACTTACGGCACC
			HL20109-V-RFP-R	CGCAGATTTCCAGCGAATGC
	F4-3/F5	866	HL20061-F4	GCGAACAACGGCCTTAAAACC
			HL20062-R3	GCTGCGACGGTCGTAAAGTAAG
	F5/F6	957	HL20040-F	CAATGGGCGTAAACGAAGTCG
			HL20041-R	TGGTAAATGGCCAACACCAAGG
	F6/F7	846	HL20063-F6	GCATTGATTGCATTTTGTGCCT
			HL20064-R5	GCGTCGAAACTTCCGTAGAC
	F7/F8	846	HL20042-F	ATTGTGGGCAATATGCCCGAA
			HL20043-R	GCAGTGTATCAACTGCGGCAC
	F8/V	710	HL20065-F8	TGCGAAGCTGCGGTTAATCTT
			LH19006-pRSII313-R	TACCGCCTTTGAGTGAGCTG
	V/F1	1,007	HL19052-V-F	ACAATGGAAGTCCGAGCTCATC
			HL20066-R7	GCGTCGGATAAGTATTACGGCG
pYEP-II-S4-b2-RFP	F1/F2	826	HL20044-F	CGAAGGTAACGAAGCATCGTTG
			HL20037-R	TCGACGGCCTGTTTAATATCGC
	F2/F3	795	HL20059-F2	GTTGGTGGATGCGCGTTATG

			HL20060-R1	TGCCCACAATAAAACCGACGT
	F3/F4-1	783	HL20038-F	TTGAATGGGCGTTGAAATGGGC
			HL20039-R	ATGCGTTCGTCGCTTCCTGTA
	F4-1/F4-3	1,372	HL20108-V-RFP-F	CGCCTGTTTACTTACGGCACC
			HL20109-V-RFP-R	CGCAGATTTCCAGCGAATGC
	F4-3/F5	866	HL20061-F4	GCGAACAACGGCCTTAAAACC
			HL20062-R3	GCTGCGACGGTCGTAAAGTAAG
	F5/F6	957	HL20040-F	CAATGGGCGTAAACGAAGTCG
			HL20041-R	TGGTAAATGGCCAACACCAAGG
	F6/F7	846	HL20063-F6	GCATTGATTGCATTTTGTGCCT
			HL20064-R5	GCGTCGAAACTTCCGTAGAC
	F7/F8	846	HL20042-F	ATTGTGGGCAATATGCCCGAA
			HL20043-R	GCAGTGTATCAACTGCGGCAC
	F8/V	462	HL20065-F8	TGCGAAGCTGCGGTTAATCTT
			HL19053-V-R	GCTCACTCATTAGGCACCCCA
	V/F1	624	LH19005-pRSII313-F	GTGGCGAGAAAGGAAGGGAA
			HL20198-V-R1	CATAAACAGCGATTCGGCGAC
	F1/F2	957	HL20040-F	CAATGGGCGTAAACGAAGTCG
pRSII313-S4-a3			HL20041-R	TGGTAAATGGCCAACACCAAGG
	F2/F3	846	HL20063-F6	GCATTGATTGCATTTTGTGCCT
			HL20064-R5	GCGTCGAAACTTCCGTAGAC
	F3/F4	846	HL20042-F	ATTGTGGGCAATATGCCCGAA

			HL20043-R	GCAGTGTATCAACTGCGGCAC
	F4/F5	808	HL20065-F8	TGCGAAGCTGCGGTTAATCTT
			HL20066-R7	GCGTCGGATAAGTATTACGGCG
	F5/F6	826	HL20044-F	CGAAGGTAACGAAGCATCGTTG
			HL20037-R	TCGACGGCCTGTTTAATATCGC
	F6/F7	795	HL20059-F2	GTTGGTGGATGCGCGTTATG
			HL20060-R1	TGCCCACAATAAAACCGACGT
	F7/F8	783	HL20038-F	TTGAATGGGCGTTGAAATGGGC
			HL20039-R	ATGCGTTCGTCGCTTCCTGTA
	F8/V	892	HL20199-V-F8	CCGAATCAAGATGGCCGTTCAC
			LH19006-pRSII313-R	TACCGCCTTTGAGTGAGCTG
	V/F1	747	HL19052-V-F	ACAATGGAAGTCCGAGCTCATC
			HL20198-V-R1	CATAAACAGCGATTCGGCGAC
	F1/F2	957	HL20040-F	CAATGGGCGTAAACGAAGTCG
			HL20041-R	TGGTAAATGGCCAACACCAAGG
pYEP-II-S4-b3	F2/F3	846	HL20063-F6	GCATTGATTGCATTTTGTGCCT
			HL20064-R5	GCGTCGAAACTTCCGTAGAC
	F3/F4	846	HL20042-F	ATTGTGGGCAATATGCCCGAA
			HL20043-R	GCAGTGTATCAACTGCGGCAC
	F4/F5	808	HL20065-F8	TGCGAAGCTGCGGTTAATCTT
			HL20066-R7	GCGTCGGATAAGTATTACGGCG
	F5/F6	826	HL20044-F	CGAAGGTAACGAAGCATCGTTG

			HL20037-R	TCGACGGCCTGTTTAATATCGC
	F6/F7	795	HL20059-F2	GTTGGTGGATGCGCGTTATG
			HL20060-R1	TGCCCACAATAAAACCGACGT
	F7/F8	783	HL20038-F	TTGAATGGGCGTTGAAATGGGC
			HL20039-R	ATGCGTTCGTCGCTTCCTGTA
	F8/V	644	HL20199-V-F8	CCGAATCAAGATGGCCGTTCAC
			HL19053-V-R	GCTCACTCATTAGGCACCCCA
	V/F1	624	LH19005-pRSII313-F	GTGGCGAGAAAGGAAGGGAA
			HL20198-V-R1	CATAAACAGCGATTCGGCGAC
	F1/F2	957	HL20040-F	CAATGGGCGTAAACGAAGTCG
			HL20041-R	TGGTAAATGGCCAACACCAAGG
	F2/F3	846	HL20063-F6	GCATTGATTGCATTTTGTGCCT
			HL20064-R5	GCGTCGAAACTTCCGTAGAC
	F3/F4	846	HL20042-F	ATTGTGGGCAATATGCCCGAA
pRSII313-S4-a3-RFP			HL20043-R	GCAGTGTATCAACTGCGGCAC
	F4/F5	808	HL20065-F8	TGCGAAGCTGCGGTTAATCTT
			HL20066-R7	GCGTCGGATAAGTATTACGGCG
	F5/F6	826	HL20044-F	CGAAGGTAACGAAGCATCGTTG
			HL20037-R	TCGACGGCCTGTTTAATATCGC
	F6/F7	795	HL20059-F2	GTTGGTGGATGCGCGTTATG
			HL20060-R1	TGCCCACAATAAAACCGACGT
	F7/F8-1	783	HL20038-F	TTGAATGGGCGTTGAAATGGGC

			HL20039-R	ATGCGTTCGTCGCTTCCTGTA
	F8-1/F8-3	1,372	HL20108-V-RFP-F	CGCCTGTTTACTTACGGCACC
			HL20109-V-RFP-R	CGCAGATTTCCAGCGAATGC
	F8-3/V	892	HL20199-V-F8	CCGAATCAAGATGGCCGTTCAC
			LH19006-pRSII313-R	TACCGCCTTTGAGTGAGCTG
	V/F1	747	HL19052-V-F	ACAATGGAAGTCCGAGCTCATC
			HL20198-V-R1	CATAAACAGCGATTCGGCGAC
	F1/F2	957	HL20040-F	CAATGGGCGTAAACGAAGTCG
			HL20041-R	TGGTAAATGGCCAACACCAAGG
	F2/F3	846	HL20063-F6	GCATTGATTGCATTTTGTGCCT
			HL20064-R5	GCGTCGAAACTTCCGTAGAC
	F3/F4	846	HL20042-F	ATTGTGGGCAATATGCCCGAA
			HL20043-R	GCAGTGTATCAACTGCGGCAC
pYEP-II-S4-b3-RFP	F4/F5	808	HL20065-F8	TGCGAAGCTGCGGTTAATCTT
			HL20066-R7	GCGTCGGATAAGTATTACGGCG
	F5/F6	826	HL20044-F	CGAAGGTAACGAAGCATCGTTG
			HL20037-R	TCGACGGCCTGTTTAATATCGC
	F6/F7	795	HL20059-F2	GTTGGTGGATGCGCGTTATG
			HL20060-R1	TGCCCACAATAAAACCGACGT
	F7/F8-1	783	HL20038-F	TTGAATGGGCGTTGAAATGGGC
			HL20039-R	ATGCGTTCGTCGCTTCCTGTA
	F8-1/F8-3	1,372	HL20108-V-RFP-F	CGCCTGTTTACTTACGGCACC

			HL20109-V-RFP-R	CGCAGATTTCCAGCGAATGC
	F8-3/V	644	HL20199-V-F8	CCGAATCAAGATGGCCGTTCAC
			HL19053-V-R	GCTCACTCATTAGGCACCCCA
	V/F1	884	LH19005-pRSII313-F	GTGGCGAGAAAGGAAGGGAA
			HL20066-R7	GCGTCGGATAAGTATTACGGCG
	F1/F2	840	LC21131-V-F	CGTTCGAAACTTGCTTGCCCG
			LC21132-V-R	CGTTAGTGCGTGCCGGAATCA
	F2/F3	826	HL20044-F	CGAAGGTAACGAAGCATCGTTG
			HL20037-R	TCGACGGCCTGTTTAATATCGC
	F3/F4	795	HL20059-F2	GTTGGTGGATGCGCGTTATG
			HL20060-R1	TGCCCACAATAAAACCGACGT
pRSII313-S4-△gp39-43	F4/F5	783	HL20038-F	TTGAATGGGCGTTGAAATGGGC
			HL20039-R	ATGCGTTCGTCGCTTCCTGTA
	F5/F6	866	HL20061-F4	GCGAACAACGGCCTTAAAACC
			HL20062-R3	GCTGCGACGGTCGTAAAGTAAG
	F6/F7	957	HL20040-F	CAATGGGCGTAAACGAAGTCG
			HL20041-R	TGGTAAATGGCCAACACCAAGG
	F7/F8	846	HL20063-F6	GCATTGATTGCATTTTGTGCCT
			HL20064-R5	GCGTCGAAACTTCCGTAGAC
	F8/F9	846	HL20042-F	ATTGTGGGCAATATGCCCGAA
			HL20043-R	GCAGTGTATCAACTGCGGCAC
	F9/V	710	HL20065-F8	TGCGAAGCTGCGGTTAATCTT

			LH19006-pRSII313-R	TACCGCCTTTGAGTGAGCTG
	V/F1	884	LH19005-pRSII313-F	GTGGCGAGAAAGGAAGGGAA
			HL20066-R7	GCGTCGGATAAGTATTACGGCG
	F1/F2	821	LC21133-V-F	GGCATTCCGGGCAACGATGCA
			LC21134-V-R	AGACCGGCCCATTTCATCATC
	F2/F3	795	HL20059-F2	GTTGGTGGATGCGCGTTATG
			HL20060-R1	TGCCCACAATAAAACCGACGT
	F3/F4	783	HL20038-F	TTGAATGGGCGTTGAAATGGGC
			HL20039-R	ATGCGTTCGTCGCTTCCTGTA
pRSII313-S4-△gp44-48	F4/F5	866	HL20061-F4	GCGAACAACGGCCTTAAAACC
			HL20062-R3	GCTGCGACGGTCGTAAAGTAAG
	F5/F6	957	HL20040-F	CAATGGGCGTAAACGAAGTCG
			HL20041-R	TGGTAAATGGCCAACACCAAGG
	F6/F7	846	HL20063-F6	GCATTGATTGCATTTTGTGCCT
			HL20064-R5	GCGTCGAAACTTCCGTAGAC
	F7/F8	846	HL20042-F	ATTGTGGGCAATATGCCCGAA
			HL20043-R	GCAGTGTATCAACTGCGGCAC
	F8/V	710	HL20065-F8	TGCGAAGCTGCGGTTAATCTT
			LH19006-pRSII313-R	TACCGCCTTTGAGTGAGCTG
pRSII313-S4-△gp50-55	V/F1	884	LH19005-pRSII313-F	GTGGCGAGAAAGGAAGGGAA
			HL20066-R7	GCGTCGGATAAGTATTACGGCG
	F1/F2	826	HL20044-F	CGAAGGTAACGAAGCATCGTTG

			HL20037-R	TCGACGGCCTGTTTAATATCGC
	F2/F3	782	LC21135-V-F	CCCGCAACCCTTGCCAAGTCT
			LC21136-V-R	AATTGCGTTGTCTTCGGCATG
	F3/F4	795	HL20059-F2	GTTGGTGGATGCGCGTTATG
			HL20060-R1	TGCCCACAATAAAACCGACGT
	F4/F5	783	HL20038-F	TTGAATGGGCGTTGAAATGGGC
			HL20039-R	ATGCGTTCGTCGCTTCCTGTA
	F5/F6	866	HL20061-F4	GCGAACAACGGCCTTAAAACC
			HL20062-R3	GCTGCGACGGTCGTAAAGTAAG
	F6/F7	957	HL20040-F	CAATGGGCGTAAACGAAGTCG
			HL20041-R	TGGTAAATGGCCAACACCAAGG
	F7/F8	846	HL20063-F6	GCATTGATTGCATTTTGTGCCT
			HL20064-R5	GCGTCGAAACTTCCGTAGAC
	F8/F9	846	HL20042-F	ATTGTGGGCAATATGCCCGAA
			HL20043-R	GCAGTGTATCAACTGCGGCAC
	F9/V	710	HL20065-F8	TGCGAAGCTGCGGTTAATCTT
			LH19006-pRSII313-R	TACCGCCTTTGAGTGAGCTG
	V/F1	884	LH19005-pRSII313-F	GTGGCGAGAAAGGAAGGGAA
			HL20066-R7	GCGTCGGATAAGTATTACGGCG
pRSII313-S4-△gp39-48	F1/F3	949	LC21131-V-F	CGTTCGAAACTTGCTTGCCCG
			LC22080-V-R	GTCGAAGCCGTGTAAGTATGCG
	F3/F4	795	HL20059-F2	GTTGGTGGATGCGCGTTATG

			HL20060-R1	TGCCCACAATAAAACCGACGT
	F4/F5	783	HL20038-F	TTGAATGGGCGTTGAAATGGGC
			HL20039-R	ATGCGTTCGTCGCTTCCTGTA
	F5/F6	866	HL20061-F4	GCGAACAACGGCCTTAAAACC
			HL20062-R3	GCTGCGACGGTCGTAAAGTAAG
	F6/F7	957	HL20040-F	CAATGGGCGTAAACGAAGTCG
			HL20041-R	TGGTAAATGGCCAACACCAAGG
	F7/F8	846	HL20063-F6	GCATTGATTGCATTTTGTGCCT
			HL20064-R5	GCGTCGAAACTTCCGTAGAC
	F8/F9	846	HL20042-F	ATTGTGGGCAATATGCCCGAA
			HL20043-R	GCAGTGTATCAACTGCGGCAC
	F9/V	710	HL20065-F8	TGCGAAGCTGCGGTTAATCTT
			LH19006-pRSII313-R	TACCGCCTTTGAGTGAGCTG
	V/F1	884	LH19005-pRSII313-F	GTGGCGAGAAAGGAAGGGAA
			HL20066-R7	GCGTCGGATAAGTATTACGGCG
	F1/F3	949	LC21131-V-F	CGTTCGAAACTTGCTTGCCCG
			LC22080-V-R	GTCGAAGCCGTGTAAGTATGCG
pRSII313-S4-△gp39-55	F3/F4	795	HL20059-F2	GTTGGTGGATGCGCGTTATG
			HL20060-R1	TGCCCACAATAAAACCGACGT
	F4/F5	783	HL20038-F	TTGAATGGGCGTTGAAATGGGC
			HL20039-R	ATGCGTTCGTCGCTTCCTGTA
	F5/F6	866	HL20061-F4	GCGAACAACGGCCTTAAAACC

		HL20062-R3	GCTGCGACGGTCGTAAAGTAAG
F6/F7	957	HL20040-F	CAATGGGCGTAAACGAAGTCG
		HL20041-R	TGGTAAATGGCCAACACCAAGG
F7/F8	846	HL20063-F6	GCATTGATTGCATTTTGTGCCT
		HL20064-R5	GCGTCGAAACTTCCGTAGAC
F8/F9	846	HL20042-F	ATTGTGGGCAATATGCCCGAA
		HL20043-R	GCAGTGTATCAACTGCGGCAC
F9/V	710	HL20065-F8	TGCGAAGCTGCGGTTAATCTT
		LH19006-pRSII313-R	TACCGCCTTTGAGTGAGCTG

Table S4 Genomic annotation of phage vB_Pae_SCUT-S4

Gene	Start	Stop	Strand	Product length (AA)	Putative function	Query coverage (%)	Identity (%)	E-value	Source*	GenBank Accession No.
1	1	255	+	84	member of phage protein family found in lysis cassettes	100	100.00	4.81E-55	vB_SmaS-DLP_2	YP_009219158.1
2	252	518	+	88	holin	100	96.59	2.32E-57	vB_Pae-Kakheti25	YP_006299866.1
3	511	1056	+	181	endolysin	100	100.00	1.25E-134	73	YP_001293410.1
4	1068	1373	+	101	Rz	100	99.01	4.39E-67	73	YP_001293411.1
5	1396	1569	+	57	Rz1	100	98.25	1.88E-36	vB_SmaS-DLP_2	YP_009219162.1
6	1627	2115	+	162	small terminase subunit	100	100.00	3.11E-118	vB_Pae-Kakheti25	YP_006299870.1
7	2096	3691	+	531	large terminase subunit	100	99.25	0.00E+00	vB_SmaS-DLP_2	YP_009219164.1
8	3705	5210	+	501	portal protein	100	99.20	0.00E+00	vB_SmaS-DLP_2	YP_009219165.1
9	5222	6316	+	364	F-like head morphogenesis protein	100	99.45	0.00E+00	vB_SmaS-DLP_2	YP_009219166.1
10	6353	7072	+	239	scaffold protein	100	99.58	2.65E-172	vB_Pae-Kakheti25	YP_006299874.1
11	7075	8049	+	324	major capsid protein	100	99.69	0.00E+00	vB_PaeS_SCH_Ab26	YP_009044345.1
12	8119	8505	+	128	hypothetical protein	100	94.03	1.67E-82	vB_Pae-Kakheti25	YP_006299876.1
13	8571	8942	+	123	virion structural protein	100	95.94	5.79E-69	vB_SmaS-DLP_2	YP_009219170.1
14	8955	9473	+	172	virion structural protein	100	100.00	8.52E-126	vB_Pae-Kakheti25	YP_006299878.1
15	9477	9857	+	126	head-tail joining protein	100	100.00	2.75E-90	vB_SmaS-DLP_2	YP_009219172.1
16	9854	10309	+	151	minor tail protein	100	100.00	6.24E-111	vB_SmaS-DLP_2	YP_009219173.1
17	10322	11857	+	511	major tail tube protein	100	99.41	0.00E+00	vB_SmaS-DLP_2	YP_009219174.1
18	11921	12349	+	142	tail chaperonin	100	100.00	7.13E-102	vB_Pae-Kakheti25	YP_006299883.1
19	12370	12714	+	114	tail chaperonin	100	99.12	1.84E-80	vB_SmaS-DLP_2	YP_009219176.1
20	12683	13117	+	144	tail completion protein	100	100.00	3.43E-103	vB_Pae-Kakheti25	YP_006299884.1
21	13123	16737	+	1204	tape measure protein	100	98.67	0.00E+00	vB_PaeS_SCH_Ab26	YP_009044355.1
22	16738	17700	+	320	virion structural protein	100	95.63	0.00E+00	vB_SmaS-DLP_2	YP_009219179.1
23	17700	18665	+	321	virion structural protein	100	95.95	0.00E+00	vB_SmaS-DLP_2	YP_009219180.1

24	18671	20383	+	570	virion structural protein	100	98.25	0.00E+00	vB_SmaS-DLP_2	YP_009219181.1
25	20383	21207	+	274	FAD/FMN-containing dehydrogenase	100	100.00	0.00E+00	vB_SmaS-DLP_2	YP_009219182.1
26	21211	23652	+	813	central tail hub	100	97.05	0.00E+00	vB_PaeS_SCH_Ab26	YP_009044360.1
27	23653	25704	-	683	DNA polymerase	100	99.27	0.00E+00	vB_SmaS-DLP_2	YP_009219184.1
28	25716	26858	-	380	replicative clamp	100	98.68	0.00E+00	vB_PaeS_SCH_Ab26	YP_009044362.1
29	26842	27069	-	75	hypothetical protein	100	100.00	1.44E-49	vB_Pae-Kakheti25	YP_006299893.1
30	27074	28729	-	551	dead box helicase	100	100.00	0.00E+00	73	YP_001293436.1
31	28722	29618	-	298	RecB exonuclease	100	98.32	0.00E+00	vB_Pae-Kakheti25	YP_006299895.1
32	29725	30252	-	175	hypothetical protein	94	88.49	1.64E-112	PaMx42	YP_009205626.1
33	30331	31089	-	252	single-stranded DNA-binding protein	100	98.41	6.88E-167	vB_SmaS-DLP_2	YP_009219190.1
34	31148	31864	-	238	RecA	100	100.00	3.77E-178	vB_SmaS-DLP_2	YP_009219191.1
35	31919	32359	-	146	hypothetical protein	100	100.00	5.34E-101	vB_SmaS-DLP_2	YP_009219192.1
36	32436	32993	-	185	MazG	100	97.30	1.14E-128	vB_PaeS_SCH_Ab26	YP_009044368.1
37	33131	33319	+	62	hypothetical protein	100	98.39	1.37E-38	vB_Pae-Kakheti25	YP_006299901.1
38	33309	35630	+	773	replicative primase/helicase	100	99.35	0.00E+00	vB_Pae-Kakheti25	YP_006299902.1
39	35782	36051	+	89	hypothetical protein	100	100.00	6.65E-59	vB_Pae-Kakheti25	YP_006299909.1
40	36155	36361	+	68	hypothetical protein	-	-	-	-	-
41	36374	36523	+	49	hypothetical protein	100	100.00	3.15E-30	PaMx42	YP_009205634.1
42	36595	36801	+	68	hypothetical protein	99	82.09	1.07E-27	vB_PaeS_SCH_Ab26	YP_009044373.1
43	36808	37065	+	85	hypothetical protein	82	45.71	8.96E-10	KL1	YP_006560791.1
44	37386	37661	+	91	hypothetical protein	100	98.90	2.14E-63	vB_Pae-Kakheti25	YP_006299906.1
45	37700	37942	+	80	hypothetical protein	100	98.75	1.58E-53	vB_SmaS-DLP_2	YP_009219201.1
46	38024	38281	+	85	hypothetical protein	-	-	-	-	-
47	38332	38706	+	124	hypothetical protein	100	97.58	5.18E-81	vB_Pae-Kakheti25	YP_006299910.1
48	38767	38988	+	73	hypothetical protein	100	100.00	2.82E-47	vB_SmaS-DLP_2	YP_009219204.1
49	38985	39521	+	178	Vsr endonuclease	100	98.88	1.11E-130	vB_Pae-Kakheti25	YP_006299912.1
50	39521	39724	+	67	hypothetical protein	100	98.51	1.62E-42	vB_PaeS_SCH_Ab26	YP_009044380.1
51	39721	39900	+	59	hypothetical protein	100	94.92	7.30E-36	vB_SmaS-DLP_2	YP_009219207.1
52	39961	40263	+	100	hypothetical protein	98	82.00	3.44E-50	73	YP_001293452.1

53	40280	40570	+	96	hypothetical protein	100	97.92	4.84E-62	vB_PaeS_SCH_Ab26	YP_009044381.1
54	40563	40796	+	77	hypothetical protein	100	100.00	2.09E-53	73	YP_001293454.1
55	40867	41034	+	55	hypothetical protein	100	98.18	8.94E-33	73	YP_001293455.1
56	41025	41492	+	155	dCMP deaminase	100	98.71	6.48E-112	73	YP_001293456.1
57	41498	41881	+	127	hypothetical protein	100	100.00	6.45E-91	73	YP_001293457.1
58	41916	42125	+	69	hypothetical protein	100	98.55	2.10E-46	73	YP_001293458.1
59	42209	42781	+	190	hypothetical protein	100	98.42	4.70E-134	vB_Pae-Kakheti25	YP_006299922.1

*These *Pseudomonas* phages belong to the *Septima3Virus* genus, along with phage vB_Pae_SCUT-S4.

Table S5 Predicted regulatory elements and the closest BLAST matches of the 16 hypothetical proteins

Gene	Start codon	Stop codon	AAs	Predicted promoter	Predicted terminator	Closest blast match (protein/phage)	Identity
39	ATG	UAA	89	+	+	hypothetical protein KAK25_00045 /*Pseudomonas* phage vB_Pae-Kakheti25	100.00%
40	ATG	UAA	68	+	-	hypothetical protein PM408_gp12 /*Pseudomonas* phage PSV3	100.00%
41	ATG	UAA	49	-	-	hypothetical protein PM395_gp51 /*Xanthomonas* phage Samson	100.00%
42	ATG	UAA	68	-	-	hypothetical protein PM402_gp07 /*Pseudomonas* phage vB_PaeS_C1	98.53%
43	ATG	UAA	85	+	+	hypothetical protein PM403_gp33 /*Pseudomonas* phage BUCT-PX-5	97.65%
44	ATG	UAG	91	+	+	hypothetical protein PM402_gp05 /*Pseudomonas* phage vB_PaeS_C1	100.00%
45	ATG	UAA	80	+	-	hypothetical protein /*Pseudomonas* phage vB_PaeS_HZ_ZJUX1	100.00%
46	ATG	UAA	85	+	+	hypothetical protein P7H99_gp48 /*Pseudomonas* phage vB_PaeS_SCUT-S3	100.00%
47	ATG	UAA	124	-	+	hypothetical protein PM408_gp04 /*Pseudomonas* phage PSV3	100.00%
48	ATG	UGA	73	+	+	hypothetical protein /*Pseudomonas* phage vB_PaeS_HZ_ZJUX1	100.00%
50	ATG	UGA	67	-	-	hypothetical protein HL17_gp45 /*Pseudomonas* phage vB_PaeS_SCH_Ab26	98.51%
51	ATG	UGA	59	-	-	hypothetical protein PM403_gp43	98.31%

						/*Pseudomonas* phage BUCT-PX-5	
52	ATG	UAA	100	+	-	hypothetical protein PM404_gp51 /*Stenotrophomonas* phage vB_SmaS-DLP_1	98.98%
53	ATG	UAA	96	+	-	hypothetical protein PSV3_00191 /*Pseudomonas* phage PSV3	98.96%
54	ATG	UAA	77	-	-	hypothetical protein ORF047 /*Pseudomonas* phage 73	100.00%
55	ATG	UAA	55	+	+	hypothetical protein ORF048 /*Pseudomonas* phage 73	98.18%

Table S6 Structural and functional predictions of the 16 hypothetical proteins using AlphaFold3

Hypothetical protein	pLDDT[1]	Tmscore[2]	Reference PDBID/annotation	Class of reference protein[3]	Source organism of reference protein[3]	Predicted function of hypothetical protein[4]
gp39	90.97	0.61	1ge5/zinc peptidase from Grifola Frondosa	Hydrolase	*Podila humilis*	Modulating host immune responses[1]
gp40	86.08	0.65	6iml/The crystal structure of AsfvLIG:CT1 complex	Ligase/DNA	African swine fever virus	DNA repair and replication[2]
gp41	91.16	0.73	8jgu/Crystal structure of N-terminal domain of exopolyphosphatase from Deinococcus radiodurans	Hydrolase	*Deinococcus radiodurans* R1	Regulating host polyP metabolism[3]
gp42	89.42	0.60	4h5b/Crystal Structure of DR_1245 from Deinococcus radiodurans	Unknown function	*Deinococcus radiodurans* R1	Unknown function
gp43	70.79	0.82	8wb7/CryoEM structure of Snf7 N-terminal domain in the inner coils of spiral	Protein transport	*Saccharomyces cerevisiae*	Membrane permeability regulation[4]
gp44	86.58	0.56	8eun/MicroED structure of an Aeropyrum pernix protoglobin metallo-carbene complex	Metal binding protein	*Aeropyrum pernix*	Regulating host metal homeostasis[5]
gp45	76.01	0.58	4adm/Crystal structure of Rv1098c in complex with meso-tartrate	Lyase	*Mycobacterium tuberculosis* H37Rv	Metabolic reprogramming[6]
gp46	78.69	0.61	7o5y/PilA minor pilin of Streptococcus sanguinis type IV pili	Structural protein	*Streptococcus sanguinis*	Attachment and invasion of phages and host[7]

gp47	64.94	0.64	8vlu/Cryo-EM structure of human HGSNAT bound with CoA	Membrane protein	*Homo sapiens*	Regulating the metabolism of host[8]
gp48	89.1	0.63	7n77/Cryo-EM structure of ATP13A2 D458N/D962N mutant in the AlF-bound E1P-like state	Transport protein	*Homo sapiens*	Transport of substances within host cells[9]
gp50	88.31	0.71	4tsm/MBP-fusion protein of PilA1 from C. difficile R20291 residues 26-166	Cell adhesion	*Clostridium difficile*	Host cell recognition and attachment[10]
gp51	93.5	0.66	3htx/Crystal structure of small RNA methyltransferase HEN1	Transferase/RNA	*Arabidopsis thaliana*	RNA modification[11]
gp52	67.32	0.63	1bp1/crystal structure of BPI, the human bactericidal permeability-increasing protein	Bactericidal	*Homo sapiens*	Interaction with the host immune system[12]
gp53	75.27	0.59	3qmz/Crystal structure of the cytoplasmic dynein heavy chain motor domain	Motor protein	*Saccharomyces cerevisiae*	Participate in the transportation of phage particles[13]
gp54	89.04	0.59	8ug4/Caenorhabditis elegans Otopetrin 8 (CeOtop8) in pH 8.0	Membrane protein	*Caenorhabditis elegans*	Interaction between phage invasion and host membrane[14]
gp55	87.1	0.91	8qi4/400A Vipp1 H1-6 helical tubes	Lipid binding protein	*Synechocystis* sp. PCC 6803	Regulating host lipid metabolism[15]

1: pLDDT:

> 90: The predicted result has a very high confidence level and is highly accurate.

90-70: The predicted result has a moderate confidence level and is acceptable for structure-related tasks. However, caution should be exercised when using it for high-precision applications.

70-50: The predicted result has a low confidence level and requires further validation through experiments.

< 50: The predicted result is unreliable and should not be used.

2: Tmscore:

< 0.2: Completely unable to match the reference structure.

> 0.5: Indicates the same fold as the reference structure.

3: From NCBI database.

4: Reference:

[1] Hori T, Kumasaka T, Yamamoto M, Nonaka N, Tanaka N, Hashimoto Y, Ueki U, Takio K. Structure of a new 'aspzincin' metalloendopeptidase from Grifola frondosa: implications for the catalytic mechanism and substrate specificity based on several different crystal forms. Acta Crystallogr D Biol Crystallogr. 2001 Mar;57(Pt 3):361-8.

[2] Chen Y, Liu H, Yang C, Gao Y, Yu X, Chen X, Cui R, Zheng L, Li S, Li X, Ma J, Huang Z, Li J, Gan J. Structure of the error-prone DNA ligase of African swine fever virus identifies critical active site residues. Nat Commun. 2019 Jan 23;10(1):387.

[3] Dai S, Wang B, Ye R, Zhang D, Xie Z, Yu N, Cai C, Huang C, Zhao J, Zhang F, Hua Y, Zhao Y, Zhou R, Tian B. Structural Evolution of Bacterial Polyphosphate Degradation Enzyme for Phosphorus Cycling. Adv Sci (Weinh). 2024 Jul;11(26): e2309602.

[4] Liu M, Liu Y, Song T, Yang L, Qi L, Zhang YZ, Wang Y, Shen QT. Three-dimensional architecture of ESCRT-III flat spirals on the membrane. Proc Natl Acad Sci U S A. 2024 May 14;121(20): e2319115121.

[5] Danelius E, Porter NJ, Unge J, Arnold FH, Gonen T. MicroED Structure of a Protoglobin Reactive Carbene Intermediate. J Am Chem Soc. 2023 Apr 5;145(13):7159-7165.

[6] Mechaly AE, Haouz A, Miras I, Barilone N, Weber P, Shepard W, Alzari PM, Bellinzoni M. Conformational changes upon ligand binding in the essential class II fumarase Rv1098c from Mycobacterium tuberculosis. FEBS Lett. 2012 Jun 4;586(11):1606-11.

[7] Shahin M, Sheppard D, Raynaud C, Berry JL, Gurung I, Silva LM, Feizi T, Liu Y, Pelicic V. Characterization of a glycan-binding complex of minor pilins completes the analysis of Streptococcus sanguinis type 4 pili subunits. Proc Natl Acad Sci U S A. 2023 Jan 17;120(3): e2216237120.

[8] Zhao B, Cao Z, Zheng Y, Nguyen P, Bowen A, Edwards RH, Stroud RM, Zhou Y, Van Lookeren Campagne M, Li F. Structural and mechanistic insights into a lysosomal membrane enzyme HGSNAT involved in Sanfilippo syndrome. Nat Commun. 2024 Jun 25;15(1):5388.

[9] Sim SI, von Bülow S, Hummer G, Park E. Structural basis of polyamine transport by human ATP13A2 (PARK9). Mol Cell. 2021 Nov 18;81(22):4635-4649.e8.

[10] Piepenbrink KH, Maldarelli GA, Martinez de la Peña CF, Dingle TC, Mulvey GL, Lee A, von Rosenvinge E, Armstrong GD, Donnenberg MS, Sundberg EJ. Structural and evolutionary analyses show unique stabilization strategies in the type IV pili of Clostridium difficile. Structure. 2015 Feb 3;23(2):385-96.

[11] Huang Y, Ji L, Huang Q, Vassylyev DG, Chen X, Ma JB. Structural insights into mechanisms of the small RNA methyltransferase HEN1. Nature. 2009 Oct 8;461(7265):823-7.

[12] Elsbach P, Weiss J. Prospects for use of recombinant BPI in the treatment of gram-negative bacterial infections. Infect Agents Dis. 1995 Jun;4(2):102-9.

[13] Carter AP, Cho C, Jin L, Vale RD. Crystal structure of the dynein motor domain. Science. 2011 Mar 4;331(6021):1159-65.

[14] Gan N, Zeng W, Han Y, Chen Q, Jiang Y. Structural mechanism of proton conduction in otopetrin proton channel. Nat Commun. 2024 Aug 23;15(1):7250.

[15] Junglas B, Kartte D, Kutzner M, Hellmann N, Ritter I, Schneider D, Sachse C. Structural basis for Vipp1 membrane binding: from loose coats and carpets to ring and rod assemblies. Nat Struct Mol Biol. 2024 Oct 8.
